# Biallelic mutations in cancer genomes reveal local mutational determinants

**DOI:** 10.1038/s41588-021-01005-8

**Published:** 2022-02-10

**Authors:** Jonas Demeulemeester, Stefan C. Dentro, Moritz Gerstung, Peter Van Loo

**Affiliations:** 1 Cancer Genomics Laboratory, The Francis Crick Institute, London, UK; 2 Department of Human Genetics, KU Leuven, Leuven, Belgium; 3 European Molecular Biology Laboratory-European Bioinformatics Institute, Hinxton, UK; 4 Wellcome Sanger Institute, Hinxton, UK

**Keywords:** Cancer, Cancer, Computational biology and bioinformatics, Genomics

## Abstract

The infinite sites model of molecular evolution posits that every position in the genome is mutated at most once^1^. By restricting the number of possible mutation histories, haplotypes and alleles, it forms a cornerstone of tumor phylogenetic analysis^2^ and is often implied when calling, phasing and interpreting variants^3,4^ or studying the mutational landscape as a whole^5^. Here we identify 18,295 biallelic mutations, where the same base is mutated independently on both parental copies, in 559 (21%) bulk sequencing samples from the Pan-Cancer Analysis of Whole Genomes study. Biallelic mutations reveal ultraviolet light damage hotspots at E26 transformation-specific (ETS) and nuclear factor of activated T cells (NFAT) binding sites, and hypermutable motifs in *POLE*-mutant and other cancers. We formulate recommendations for variant calling and provide frameworks to model and detect biallelic mutations. These results highlight the need for accurate models of mutation rates and tumor evolution, as well as their inference from sequencing data.

## Abstract (web summary)

Common mutational analyses assume that every base in the genome can only mutate once. Here, the authors report multiple violations of this infinite sites model in whole-genome data across a range of tumor types.

## Main

Recent studies have shown systematic variation in mutation rates across the genome, resulting in specific hotspots^5–7^. In addition, breakdown of the infinite sites assumption at the scale of individual single-nucleotide variants (SNVs) was inferred from single-cell tumor sequencing data and flagged as a confounder during phylogenetic reconstruction^8^. In bulk tumor data, population averaging and limited long-range information make it difficult to assess mutational recurrence and its impact on analyses.

**Fig. 1:**
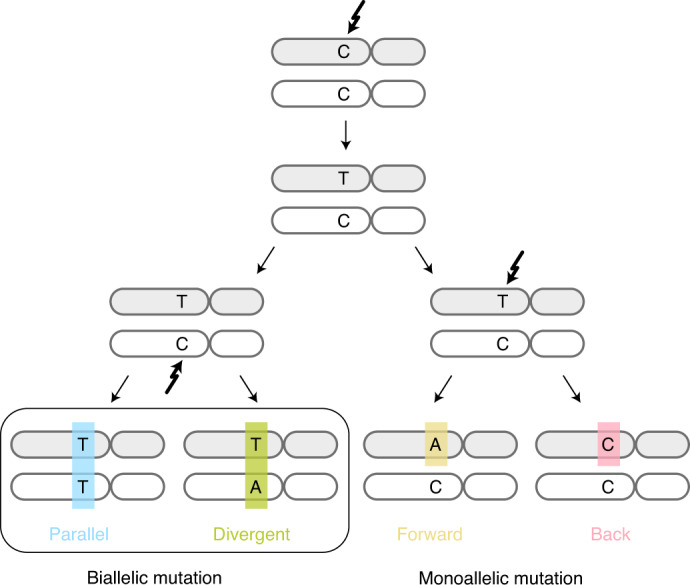
Possible violations of the infinite sites assumption in a single clonal lineage. Two subsequent mutations at a diploid locus can affect the same or alternate alleles. Depending on the base changes, there are four scenarios: biallelic parallel or divergent mutations affect separate alleles, whereas monoallelic forward and back mutations hit the same allele twice.

In a single diploid lineage, four classes of infinite sites violations may be considered (Fig. 1): (1) biallelic parallel and (2) biallelic divergent, where two alleles independently mutate to the same or different alternate bases, respectively; (3) monoallelic forward and (4) monoallelic back, where one variant is mutated to another or back to wild type (WT), respectively. We focused on biallelic mutations, which become problematic when artificially treating genomes as haploid, hypothesizing these may be observed directly in bulk tumor genome sequencing data. Loss of variants owing to large-scale genomic deletion does not strictly contradict the infinite sites assumption, yet should be accounted for in cancer genomes^2,8,9^.

**Fig. 2:**
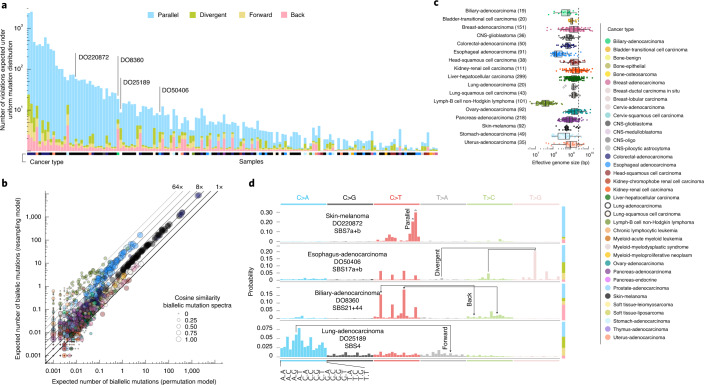
Simulated landscape of infinite sites violations in the PCAWG cohort. **a**, Number and type of infinite sites violations in 147 PCAWG samples with ≥1 expected violation under a uniform mutation distribution. The bar height indicates the expected number of violations and the colored subdivisions represent the fractions contributed by each violation type. Tumor type of the samples is color-coded below the bars. The four samples highlighted in **d** are indicated. **b**, Comparison of the expected biallelic violations from the uniform permutation and neighbor resampling models. Every dot represents a tumor simulated 1,000 times with each model. Color and size reflect, respectively, tumor type and the cosine similarity of the predicted biallelic mutation spectra. **c**, Box and scatterplot showing the effective genome size perceived by the mutational processes per cancer type, as estimated from the per-sample differences between simulation approaches. The dashed line indicates the callable genome size. The effective genome size is smallest in Lymph-BNHL (approximately 37 Mb), likely driven by recurrent focal hypermutation^13^. Center line, median; box limits, upper and lower quartiles; whiskers, 1.5× interquartile range. Only tumors with ≥10 biallelic mutations across 1,000 simulations are included and their numbers are indicated between parentheses next to the tumor type. Only tumor types with ≥10 such tumors are shown. CNS, central nervous system. **d**, Mutation spectra of four tumors with distinct violation contributions indicated in **a**. The 16 distinct trinucleotide contexts are provided on the *x* axis for C>A type substitutions and are the same for each colored block. The proportion of parallel, divergent, back and forward mutations is indicated in the stacked bar on the right. Frequent combinations of mutations leading to specific infinite site violations are highlighted as well as the signatures generating them.

**Extended Data Fig. 1:**
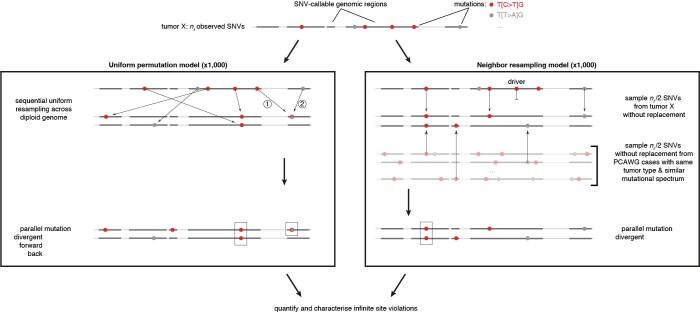
Simulation approaches for infinite sites violations. Schematic overview of the uniform permutation (left) and neighbor resampling (right) approaches to assess the number and type of infinite sites violations expected in a tumor. Numbers in the uniform permutation panel highlight the sequential nature of the sampling, which keeps track of mutated positions to consider them accordingly for biallelic, forward, and back mutation. Note that the neighbor resampling model excludes all PCAWG annotated driver mutations and allows simulation of biallelic events only.

**Extended Data Fig. 2:**
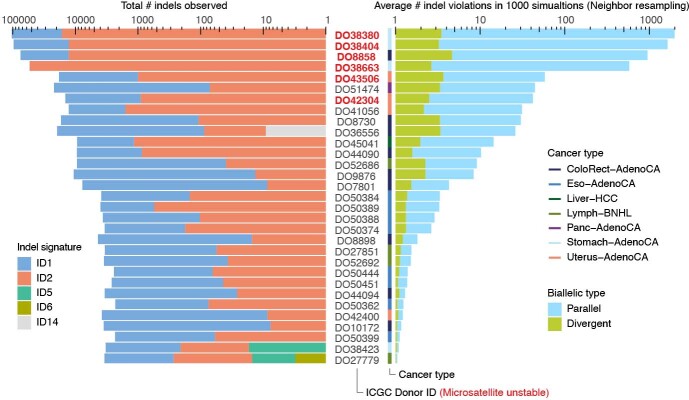
Biallelic indels are expected in a subset of microsatellite unstable tumors. Bar plots of the observed indel burden and signature (left) and the expected biallelic indels according to the neighbor resampling model (right). Bar height indicates total numbers and colored subdivisions represent fractions contributed by each indel signature (left) or biallelic indel type (right). Only PCAWG tumors with ≥1 expected biallelic indel are shown. Four microsatellite unstable tumors are predicted to boast several hundreds to over one thousand, mostly parallel, biallelic indels. These mainly originate from indel signatures 1 and 2, likely reflecting slippage during DNA replication and subsequent 1 bp T (or A) insertion and deletion in thymine (adenosine) mononucleotide repeats, respectively.

To assess the landscape of infinite sites violations, we started with a simulation approach using the Pan-Cancer Analysis of Whole Genomes (PCAWG) dataset of 2,658 whole-genome sequenced cancers. We resampled a tumor’s observed mutations, preserving mutational signature exposures^10,11^ but otherwise assuming uniform mutability across the callable diploid genome (uniform permutation model; Extended Data Fig. 1 and Supplementary Table 1). Since mutation rates are certainly not uniform and any deviation increases the number of violations^5^, this derives a lower bound of at least 1, typically parallel, violation in 147 tumors (5.5%; Fig. 2a). A second simulation approach, resampling (without replacement, nondriver) mutations from tumors of the same cancer type with similar mutational signature activities, confirms these observations (neighbor resampling model; Fig. 2b, Extended Data Fig. 1 and Supplementary Table 2). In addition, this approach indicated that four microsatellite unstable tumors harbored hundreds of parallel biallelic indels (Extended Data Fig. 2). Consistent differences between the simulators, in the number of violations per tumor type, inform on the nonuniformity of the mutational processes, that is, a reduced ‘effective genome size’ (akin to the population genetics concept of effective population size; Fig. 2c).

Distinct preferences for parallel, divergent, forward and back mutations may be understood from the active mutational processes (Fig. 2d). For instance, the dominant mutagenic activity of ultraviolet (UV) light in cutaneous melanoma (single base substitution signature 7a/b, SBS7a/b) yields almost uniquely C>T substitutions in CC and CT contexts^10,11^, which can only result in the accumulation of biallelic parallel mutations. In contrast, in esophageal adenocarcinoma DO50406, interplay between SBS17a and SBS17b^10,11^ results in various substitutions of T in a CTT context, generating both parallel and divergent variants. Back and forward mutations occur when the variant allele retains considerable mutability.

**Fig. 3:**
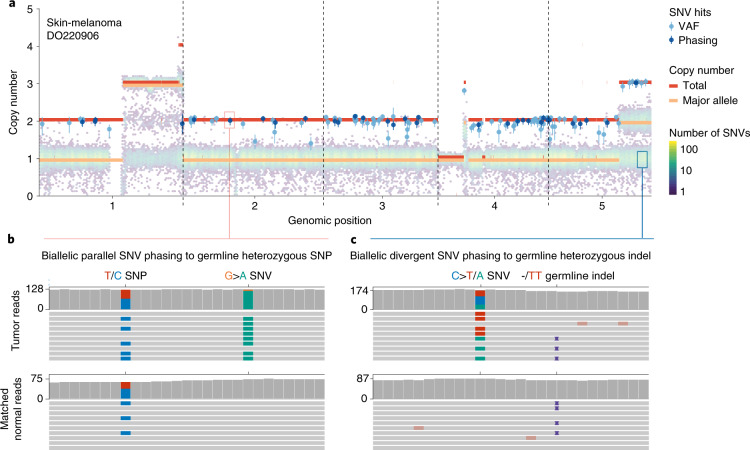
Detecting biallelic mutations in a case of melanoma. **a**, Tumor allele-specific copy number and binned mutation copy number plotted for chromosomes 1–5 of melanoma DO220906. Somatic SNVs with a mutation copy number exceeding that of the major allele (and equal to the total copy number) are evident, suggesting biallelic parallel mutation events. The error bars and their centers represent the posterior 95% highest density interval and maximum likelihood estimate, respectively obtained from a Beta-binomial model of the observed reference and alternate allele read counts with a uniform Beta(1,1) prior (Methods). **b**,**c**, Integrative Genomics Viewer visualization of DO220906 tumor (top) and matched normal (bottom) sequencing data at two loci, illustrating how read phasing information can confirm independent mutation of both parental alleles for parallel (**b**) and divergent (**c**) mutations. Reads (horizontal bars) were downsampled for clarity and local base-wise coverage is indicated to the left of the histograms. In total, we identified 373 parallel mutations (74 supported by phasing) and 8 divergent mutations in DO220906.

**Extended Data Fig. 3:**
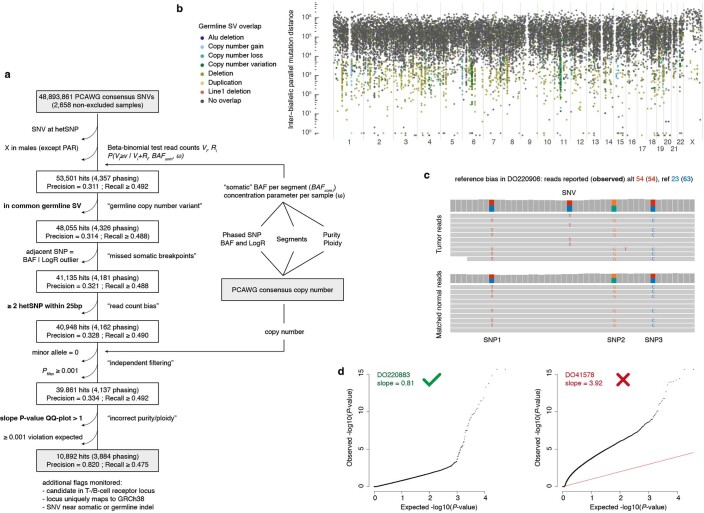
Detection of biallelic parallel mutations by allele frequency. (**a**) Flow chart showing the filtering steps, phasing-based estimates of precision and lower bound recall, as well as the input and output data for our pipeline to detect biallelic parallel mutations in PCAWG based on variant allele frequencies (VAF). Three filtering steps highlighted in bold are further illustrated in panels (b-d). (**b**) Rainfall plot of all biallelic parallel hits obtained after omitting the germline SV filter. Streaks of colored dots indicate a clustering of hits in regions with common germline structural variants. While demonstrating the ability of the pipeline to detect VAF outliers, these hits are poorly supported by phasing data and likely represent single somatic SNVs in the context of a heterozygous germline deletion. (**c**) Example of reference bias in the PCAWG consensus SNV read counts. Reads carrying the somatic variant contain alternate germline alleles at three proximal positions, resulting in an underreporting of the number of wild type reads and an overestimation of the VAF. (**d**) Diagnostic QQ-plots of the unadjusted one-sided beta-binomial read count test *P*-values for two samples (Methods). DO41578 *P*-values are overinflated (slope > 1), hinting at consensus purity/ploidy errors, and the sample is excluded as a result.

**Extended Data Fig. 4:**
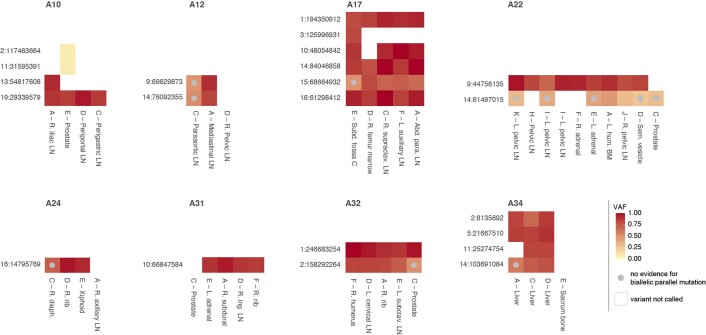
Biallelic parallel mutation during metastatic prostate cancer evolution. Heatmap showing allele frequencies of variants found to be biallelic in at least one sample of eight prostate cancers with sequencing of matched primary and metastases (A10–A34, different sites indicated as in Gundem et al.^12^). Early clonal biallelic mutations are detected in all samples of a patient (*for example*, A10 chr19:29,339,579), while late clonal and subclonal ones show no evidence of being biallelic in some samples (beta-binomial p-value > 0.05 and no discordant phasing to a heterozygous germline SNP) or are detected in only a subset of samples (*for example*, A22 chr14:61,497,015 and A10 chr2:117,463,664, respectively).

We next set out to directly detect biallelic mutations in PCAWG genomes. Parallel mutation increases the variant allele frequency (VAF) and may be distinguished from local copy number gains by comparing the VAF to the allele frequencies of neighboring heterozygous SNPs, taking tumor purity and copy number into account. Additionally, when proximal to a heterozygous germline variant, read phasing can evidence mutation of both alleles (Fig. 3a,b, Extended Data Fig. 3 and Supplementary Table 3). Without phasing information, we can only detect parallel mutations on more copies than the major allele tumor copy number. Hence, no parallel mutations are called in regions with loss of heterozygosity and late or subclonal events are likely to be underrepresented. Insights into the latter can be glimpsed from multi-sample studies. In a cohort of patients with metastatic prostate cancer with sequencing of matched primary and metastases^12,13^, we discerned early clonal (preceding the most recent common ancestor) as well as candidate late and subclonal events (Extended Data Fig. 4).

Divergent mutations can be picked up by variant callers but are traditionally filtered out^3^. Since neither the PCAWG consensus nor the four contributing pipelines reported divergent mutations, we recalled mutations with Mutect2 for 195 relevant cases, allowing 2 alternative alleles (Fig. 3c and Supplementary Tables 4 and 5). Overall, recalling identified a median 96.3% of consensus variants and added 9.5% new variants, with 0.04% of the latter contributed by divergent mutations (Supplementary Fig. 1). For 90% of divergent mutations, 1 of the alternate alleles was reported in the PCAWG consensus.

**Extended Data Fig. 5:**
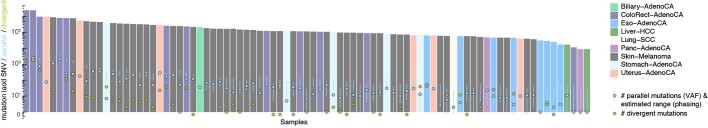
Landscape of biallelic mutations across PCAWG. Number of observed parallel (red) and divergent (blue) mutations plotted in context of the total SNV burden for 84 PCAWG samples with ≥ 1 phasing-confirmed VAF hit. The range of parallel mutations expected purely from SNV-SNP phasing is also indicated (95% confidence interval, red vertical bars) as this approach is less sensitive to purity and copy number state than the VAF-based analysis. Samples for which the number of divergent mutations is not shown were not considered for Mutect2 recalling.

In total, we identified 5,330 divergent mutations, 12,937 parallel SNVs and 14 dinucleotide variants in 559 (21%) PCAWG samples (Supplementary Tables 3–5). Parallel mutations confirmed by phasing were found in tumors with as few as 8,892 SNVs while divergent mutations were repeatedly identified in esophageal adenocarcinomas with 20,000–30,000 SNVs (Extended Data Fig. 5). At the other end of the spectrum, phasing indicated that 2 ultra-hypermutated colorectal adenocarcinomas each boasted around 8,000 parallel and 1,700 divergent mutations.

**Fig. 4:**
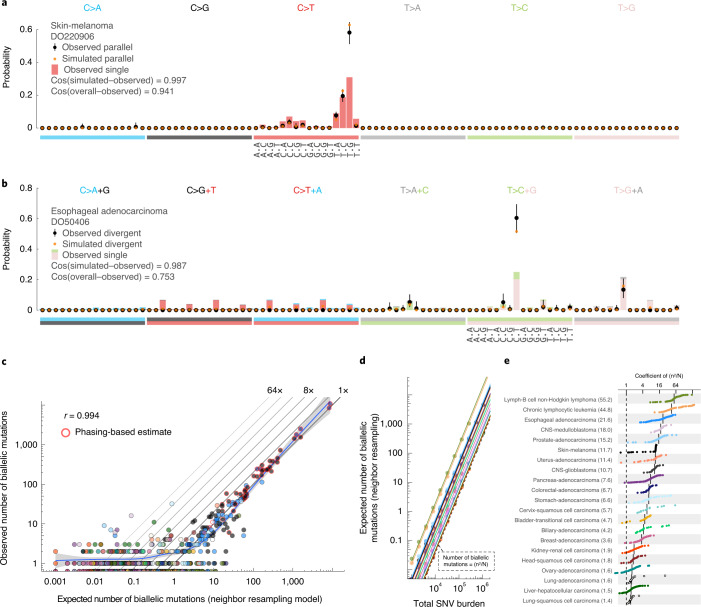
Comparison between observed and simulated biallelic mutations. **a**, Bar chart highlighting the mutation spectrum of observed and predicted parallel mutations (circles) and the background SNVs for melanoma DO220906 (bars). Cosine similarities between the spectra are indicated. The error bars represent the 95% confidence intervals obtained from a Dirichlet-multinomial model of the observed biallelic parallel mutation type counts with a uniform Dirichlet prior. **b**, Similar to **a** but showing divergent mutations for esophageal adenocarcinoma DO50406. The bars are stacked to reflect the frequency of the color-coded base changes indicated on top. **c**, Scatterplot of the observed versus neighbor resampling model-expected number of biallelic mutations (parallel + divergent) for all PCAWG tumors. For cases with ≥10,000 phaseable SNVs (red borders), the phasing-based number is provided. Colors reflect tumor type as in Fig. 2. The Pearson correlation and a spline regression fit with 95% confidence interval (shaded gray) are shown. **d**, Number of biallelic violations expected according to the neighbor resampling model for a range of mutation burdens and tumor types. The dashed line indicates the birthday problem estimate equal to the square of the mutation burden divided by the genome size (*n*^2^/*N*). The full colored lines are the linear fits per tumor type. **e**, Bar plot of the fitted coefficients of *n*^2^/*N* as derived in **d**.

Biallelic mutations carry a footprint determined by, but distinct from, the overall mutational profile. For example, since parallel mutations require two independent identical hits, they show a mutation spectrum similar to the square of that of SNVs (Fig. 4a,b). Indeed, the observed biallelic mutations were better explained by the simulated violation spectra than the overall mutation spectra (*P* = 2.83 × 10^−4^ and 1.35 × 10^−8^ for parallel and divergent, respectively; median simulated–observed cosine similarities 0.968 and 0.944; Mann–Whitney *U*-test, samples with ≥10 violations). This further supports the accuracy of our biallelic mutation calls, excluding major contributions from sequencing and alignment artifacts, germline variants, focal tandem duplicator phenotypes, precursor lesions or somatic gene conversion.

**Extended Data Fig. 6:**
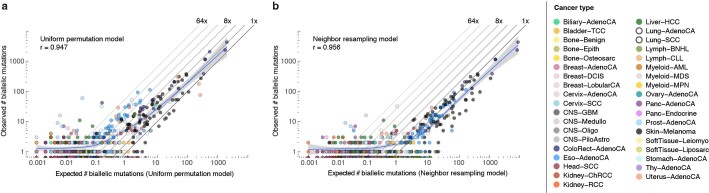
Comparison between observed and simulated biallelic mutations. (**a,b**) Scatterplots of the observed *vs*. expected number of biallelic mutations (parallel + divergent) for all PCAWG tumors using the uniform permutation (**a**) and neighbor resampling models (**b**). The Pearson correlation and a spline regression fit with 95% confidence interval (shaded grey) are shown.

While the uniform permutation model underestimates, neighbor resampling accurately predicts the number of biallelic mutations (Fig. 4c and Extended Data Fig. 6). Resampling mutation burdens and tumor types with the confirmed model demonstrates how biallelic mutations are proportional to the square of the mutation burden (*n*^2^; Fig. 4d). The coefficient per tumor type (*C*_type_) scales the callable genome size (*N*) and provides straightforward estimation of the number of violations as $C_{\mathrm{type}}n^2/N$ (Fig. 4d,e).

**Fig. 5:**
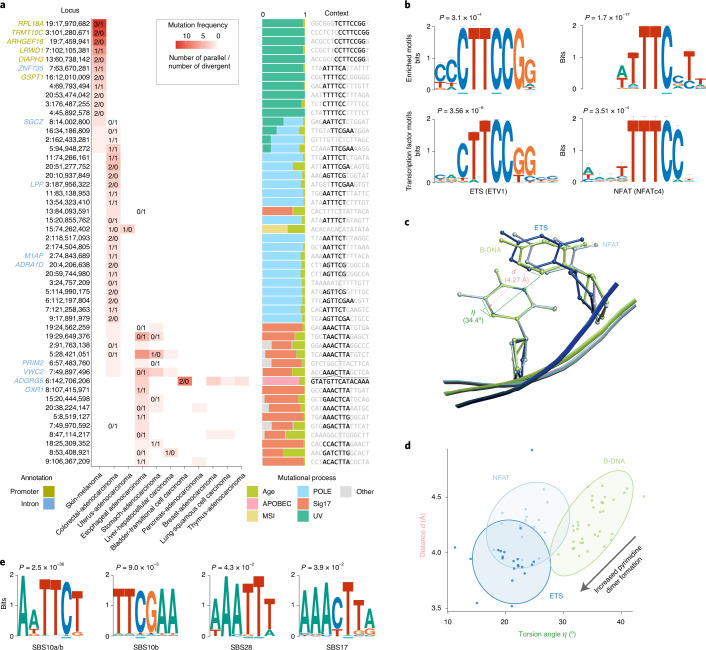
Biallelic mutations reveal hotspot motifs. **a**, Heatmap of the 50 most frequently mutated loci in PCAWG with at least 1 biallelic mutation. The number of parallel/divergent mutations at each site is indicated, as are gene annotations, the underlying mutational processes and the local sequence context with emerging motifs. For chr6:142,706,206, part of the stem and loop of a local sequence palindrome are indicated. MSI, microsatellite instability. **b**, Sequence logos of motifs enriched at loci with biallelic mutations in melanoma (top) and corresponding transcription factor recognition sequences (bottom). The error bars indicate the confidence of a motif based on the number of sites used in its creation. A Fisher exact test was used to assess motif enrichment (top) while *P* values for motif comparison (bottom) were computed and corrected for multiple testing according to Gupta et al.^17^. **c**, Superposition of Tp**C** dinucleotides in crystal structures of ETS-bound (GABP), NFAT-bound (NFAT1c) and free B-form DNA (PDB ID: 1AWC, 1OWR and 1BNA, respectively). The distance *d* between the midpoints of the two adjacent C5–C6 bonds as well as their torsion angle *η* is indicated. **d**, Scatter plot showing the distance *d* and angle *η* indicated in **c** for Tp**C** dinucleotides in structures of ETS-bound (dark blue), NFAT-bound (blue) or free B-form DNA (green) obtained from the RCSB PDB (Supplementary Table 7). The ellipses represent the normal probability contours of each group. Lower values of *d* and *η* increase the yield of UV-based pyrimidine dimer formation, as indicated by the arrow. **e**, Sequence logos of motifs enriched at loci with biallelic mutations in colorectal adenocarcinoma (SBS10, 28) and esophageal/stomach adenocarcinoma (SBS17). A Fisher exact test was used to assess motif enrichment.

**Extended Data Fig. 7:**
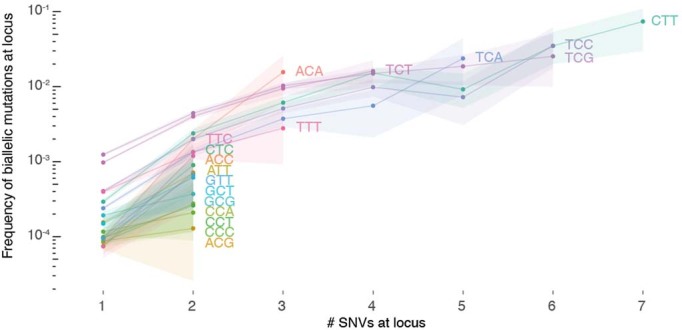
Loci with biallelic mutations have higher intrinsic mutability. The fraction of loci with biallelic mutations is plotted for loci with 1, 2, …, 7 monoallelic SNVs across PCAWG. Loci are further stratified per trinucleotide context and those with annotated driver mutations are excluded. Bootstrap resampling is performed to obtain 95% confidence intervals (shaded).

**Extended Data Fig. 8:**
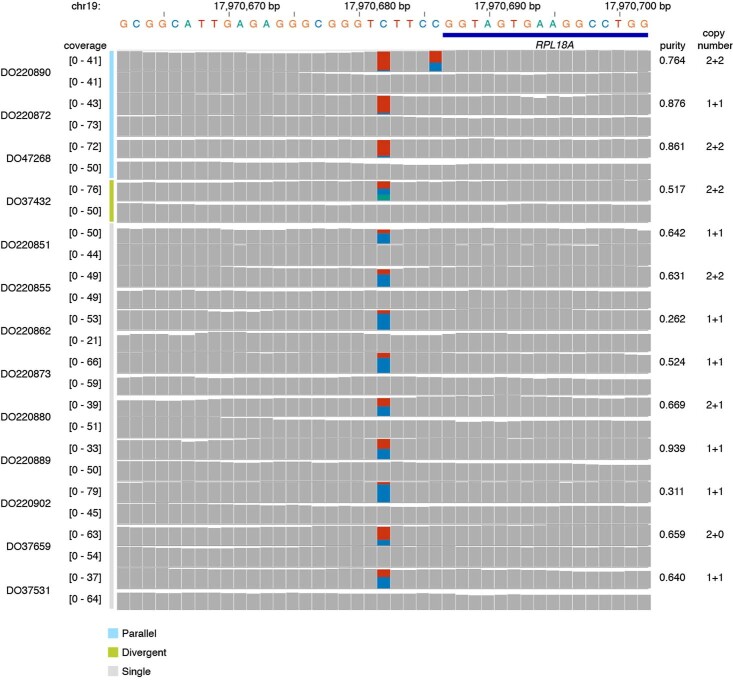
Recurrent mono- and biallelic mutation of the *RPL18A* promoter. Histograms of read coverage in 13 melanoma tumor-normal pairs showing mono- or biallelic mutation of the ETS-binding T**C**TTCCG motif at the *RPL18A* promoter.

**Extended Data Fig. 9:**
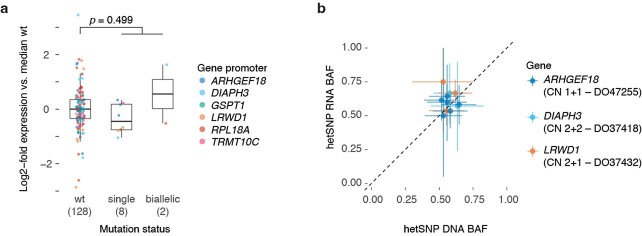
Effect of promoter mutation on gene expression for genes with biallelic hits. (**a**) Box and scatter plot showing the log2-fold change in expression (FPKM-UQ, Methods) compared to the median wild type for promoter mutated genes in Fig. 5a. Each dot represents the relative expression in a single PCAWG melanoma with RNA-Seq data, stratified by the mutation status of that gene’s promoter. The total number of tumors for each category is indicated between parentheses. Centre line, median; box limits, upper and lower quartiles; whiskers, 1.5x interquartile range. A two-sided Student’s *t*-test was used to evaluate the difference between the log2-transformed expression values of wild type vs the pooled single and biallelic mutant cases. (**b**) Scatter plot of the DNA and RNA B-allele frequencies of expressed germline heterozygous SNPs in the genes/samples with a single mutant promoter allele in (a). The ICGC donor ID and local consensus copy number are indicated. Error bars and the centre represent, respectively, the posterior 95% highest density interval and maximum likelihood estimate of the DNA and RNA B-allele frequencies assuming a uniform $Beta\left( {1,1} \right)$ prior and a binomial likelihood for the allele counts.

Biallelic mutations are not associated with somatic rearrangements (*P*_adj_ ≥ 0.31; Mann–Whitney *U*-test, Benjamini–Hochberg-corrected) but occur at loci with a higher mutation rate (Extended Data Fig. 7), some of which harbor recurrent biallelic events (Fig. 5a). The promoter of *RPL18A* shows 3 parallel, 1 divergent and 9 single mutations at chr19:17,970,682, all in melanoma (12% total; Extended Data Fig. 8) (ref. ^14^). Motif enrichment at loci with biallelic versus trinucleotide-matched monoallelic hits in melanoma reveals enrichment of Y**C**TT**C**CGG and WTTT**C**C motifs (Fig. 5a,b) (ref. ^14^). Y**C**TT**C**CGG motifs are recognized by E26 transformation-specific (ETS) transcription factor family members. Binding increases their sensitivity to UV damage due to perturbation of the Tp**C** C5–C6 interbond distance *d* and torsion angle *η*, favoring cyclobutane pyrimidine dimer formation (Fig. 5c,d) (refs. ^15,16^). The WTTT**C**C motif matches the recognition sequence for nuclear factor of activated T cells (NFAT) transcription factors^17,18^. Analysis of crystal structures of NFATc1–4 bound to DNA indicates that binding induces similar, less outspoken Tp**C** conformational changes that may explain its increased mutability (Fig. 5d and Supplementary Table 6). While we cannot formally exclude selection as a contributor to these recurrent mutations, no effects on total or allele-specific expression of genes with biallelic promoter mutations could be observed (Extended Data Fig. 9).

Similar analysis in colorectal adenocarcinoma revealed special cases of the SBS10a/b and SBS28 sequence contexts, which are associated with *POLE* exonuclease domain mutations (Fig. 5a,e)^10,11,19^. AWTT**C**T and TT**C**GAA for SBS10 and AAA**TT**T for SBS28 all carry extra adenosine and thymine bases surrounding the regular trinucleotide context of the mutated C and T, respectively. Likewise, AT-rich sequences surrounding the canonical SBS17 C**T**T context render some loci hypermutable in esophageal and stomach adenocarcinomas (AAAC**T**TA motif; Fig. 5a,e). These preferences have also been observed in the recent extension from tri- to pentanucleotide signatures^11^. However, it is unclear how these additional bases increase local mutability. Lastly, it is worth highlighting recurrent (biallelic) mutation at chr6:142,706,206, in an intron of *ADGRG6* (Fig. 5a). The CTCTTTGTAT-GTT**C**-ATACAAAGAG palindrome may adopt a hairpin structure, exposing the hypermutable C in a 4-base pair (bp) loop and rendering it susceptible to APOBEC3A deamination^7^.

Biallelic hits provide insights beyond mutational processes. The rate of biallelic mutation is proportional to that of parallel mutation between clones and increases with both the number of lineages considered and total mutation burden (Supplementary Fig. 2). When constructing phylogenies from ever more exhaustive multi-sample or single-cell data^20,21^, biallelic mutations provide an estimate for the number of parallel events.

**Extended Data Fig. 10:**
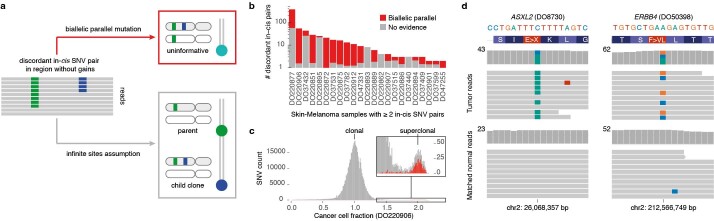
Biallelic mutations can confound common analysis. (**a**) Patterns of in-*cis* SNV pairs in a diploid region evidence linear phylogenies (parent-child) when the infinite sites assumption holds. (**b**) Bar plot showing the number of in-*cis* SNV pairs in PCAWG melanoma samples with at least two such pairs. Bar height reflects total numbers observed while the red portion indicates the fraction of all pairs with evidence for biallelic parallel mutation (beta-binomial *p*-value ≤ 0.05 or phasing to a heterozygous SNP). (**c**) Histogram of cancer cell fractions of SNVs in melanoma DO220906. The clonal cluster and a superclonal cluster containing mainly biallelic parallel mutations (red), are indicated. (**d**) IGV visualisation of two missed biallelic drivers in colorectal and oesophageal adenocarcinomas DO8730 and DO50398, respectively. Reads (horizontal bars) are downsampled for clarity and local base-wise coverage is indicated left of the histograms.

Using single-sample bulk sequencing to establish evolutionary relationships between subclones is challenging^4,22^. Under the infinite sites assumption, one can examine rare pairs of phaseable SNVs in regions without copy number gains^4,23^. Specifically, a pattern where one SNV is only found on a subset of the reads reporting the other evidences a linear relationship (Extended Data Fig. 10a). In PCAWG melanomas, however, a median 67% of these pairs in diploid regions reflect phylogenetically uninformative biallelic parallel mutations (Extended Data Fig. 10b). To avoid biasing phylogenies, biallelic SNVs should be filtered by restricting analyses to haploid regions or scrutinizing the VAF and the likelihood of biallelic mutation in the sample^4^. SNV clustering approaches, which rely on the infinite sites assumption for subclonal reconstruction and assignment of each variant to a specific lineage, may pick up ‘superclonal clusters’ of biallelic parallel mutations but are otherwise expected to remain robust at the levels identified in this study (Extended Data Fig. 10c) (ref. ^22^).

Phasing is also used to boost the accuracy of variant callers for single-molecule sequencing data^24^. As with multi-allelic variants, relaxation of the set of allowed haplotypes will need to be considered to capture the full extent of somatic variation. Indeed, while only 2.8% of biallelic hits fall within or near exons, we identified 8 candidate biallelic driver events. Parallel nonsense mutations in the tumor suppressors *ASXL2* and *CDKN2A* and divergent events in *ERBB4* suggest that, in rare cases, biallelic mutations are selected for (Extended Data Fig. 10d and Supplementary Table 7).

Taken together, we identified 18,295 biallelic mutations in 21% of PCAWG cases, demonstrating how the infinite sites assumption breaks down at the bulk level for a considerable fraction of tumors. By extension, the model is untenable in most, if not all, tumors at the multi-sample or single-cell level. If not correctly identified, biallelic mutations confound variant interpretation, ranging from driver inference to subclonal clustering and timing analyses, as well as phylogenetic inference. Nevertheless, at-scale detection of biallelic mutations affords an intimate look at the mutational processes operative in cells, such as hotspots, hypermutable motifs and the molecular mechanisms of DNA damage and repair.

## Methods

### SNV calling

PCAWG consensus single- and multi-nucleotide variant calls were obtained from the International Cancer Genome Consortium (ICGC) (http://dcc.icgc.org/releases/PCAWG/consensus_snv_indel/). Briefly, these calls were constructed according to a ‘2+ out of 4’ strategy, where calls made by at least two callers (the three Broad, EMBL/DKFZ and Sanger core PCAWG pipelines plus MuSE v.1.0) were selected as consensus calls^13^. Postmerging, these calls were subject to further quality control including filtering against oxidative artifacts (OxoG), alignment (Burrows–Wheeler Aligner versus BLAST-Like Alignment Tool), or strand biases resulting from different artifact-causing processes, as well as checks for tumor-in-normal and sample cross-contamination. Crucially, care was taken to avoid ‘bleed-through’ of germline variants into the somatic mutation calls. Specifically, absence from the Broad panel of normals based on 2,450 PCAWG samples and a higher read coverage (≥19 reads with at most 1 read reporting the alternate allele) in the matched normal sample were required to call a somatic mutation at 1 of the >14 M common (>1%) polymorphic loci of the 1000 Genomes Project. SNVs that overlapped a germline SNV or indel call in the matched normal were also removed. The sensitivity and precision of the final consensus somatic SNV calls were 95% (90% confidence interval (CI) = 88–98) and 95% (90% CI = 71–99), respectively, as evaluated by targeted deep-sequencing validation^13^. Of note, 18 biallelic parallel mutations identified in this study were covered by the PCAWG validation effort with 17 passing and 1 not being observed.

To identify biallelic divergent variants, which are filtered out in PCAWG, we recalled variants on 195 non-graylisted^13^ PCAWG tumor-normal pairs (not showing any tumor-in-normal contamination) where we might reasonably expect to find such mutations according to our uniform permutation simulations. Included also, as an internal control, were all other samples from the MELA-AU cohort (skin cancer-Australia), which met these criteria but where we did not expect biallelic divergent mutations. SNVs and indels were called using Mutect2 (Genome Analysis Toolkit (GATK) v.4.0.8.1) on the base quality score-recalibrated PCAWG BAM files and filtered according to best practices^25^. The Genome Aggregation Database (gnomAD) was provided as a germline resource and an additional panel of normals was derived from all matched normal cases. To prevent filtering of biallelic variants, FilterMutectCalls was run with --max-alt-allele-count 2. Additional filtering against germline SNPs was done by requiring a posterior probability for the alternative allele to be germline (P_GERMLINE) < −1 for both of the alternate alleles and requiring a minimal depth of 19 high-quality reads (mapping quality ≥ 35 and base quality ≥ 20) in the matched normal sample.

### Consensus copy number, purity and ploidy

PCAWG consensus copy number, tumor purity and ploidy were obtained from the ICGC^4,13^ (http://dcc.icgc.org/releases/PCAWG/consensus_cnv/). Briefly, each cancer’s genome was first segmented into regions of constant copy number using six individual copy number callers: ABSOLUTE; ACEseq; Battenberg; cloneHD; JaBbA; and Sclust, run as detailed in Dentro et al.^4^. Consensus segment breakpoints were determined from the PCAWG consensus structural variants (http://dcc.icgc.org/releases/PCAWG/consensus_sv/) complemented with high-confidence breakpoints identified by several of the copy number callers. The six callers were then rerun, enforcing this consensus segmentation and separately established consensus tumor ploidy, which was typically obtained by resolving disagreement on whether a whole-genome duplication had occurred by an expert panel^4^. The allele-specific copy number calls were combined by looking, for each segment, at the agreement in major and minor allele copy number states between callers. Lastly, consensus on tumor purity was obtained by combining the calls from the six copy number callers with those from subclonal architecture reconstruction methods that leverage SNV data: CliP; CTPsingle; PhyloWGS; cloneHD; and Ccube, as detailed in Dentro et al.^4^. This multitiered approach yielded a purity for every tumor and a quality-tiered copy number for every consensus segment.

### Simulating infinite sites violations

To estimate the number of infinite sites violations in tumors, we developed two distinct simulation approaches leveraging the PCAWG consensus SNV calls.

Our uniform permutation model resamples the observed SNVs in a tumor uniformly across the callable regions of the chromosomes, according to the observed trinucleotide-based mutational spectrum. A single simulation proceeds as follows. First, the total mutational load *n*_*t,*simulated_ is resampled from a gamma-Poisson mixture where the Poisson rate parameter $$\lambda \sim {{{\mathrm{Gamma}}}}$$ with mode equal to the observed mutational load $$n_{t,\mathrm{obs}}$$ and an s.d. of $$\sigma = 0.05 \times n_{t,\mathrm{observed}}$$, that is, $$n_{t,\mathrm{simulated}}\sim\mathrm{Poisson}\left( {\lambda \sim\mathrm{Gamma}\left( {r,\,\beta } \right)} \right)$$ where the rate of the Gamma distribution was $$r = n_{t,\mathrm{observed}} + \sqrt {n_{t,\mathrm{observed}}^2 + 2\sigma ^2} /2\sigma ^2$$ and the shape was $$\beta = 1 + n_{t,\mathrm{observed}} \times r$$. Mimicking the observed distribution, these mutations are then divided across the chromosomes according to a Dirichlet-multinomial model with $$n_{t,\mathrm{simulated}}$$ trials and parameter vector ***α*** where ***α***_*i*_ is equal to 1 + the total mutational burden on chromosome *i*. That is, *n*_**simulated**_ ~ *Mult*(*n*_*t*,simulated_, *π* ~ *Dir*(***α***)) with ***α*** = (*n*_1,observed_, *n*_2,observed_,…,*n*_*X*,observed_) + 1. Next, mutation spectra per chromosome (*π*_***i***_) were sampled from a Dirichlet distribution with parameter vector ***μ***_***i***_ where ***μ***_*i,j*_ is equal to a pseudocount $$\psi _j$$ derived from the overall mutational spectrum plus the observed number of mutations of type *j* on chromosome *i*, that is, ***π***_*i*_ ~ *Dir*(***μ***_*i*_) with $${{{\mathbf{\upmu }}}}_{{{\mathbf{i}}}} = \left( {\mu _{i,A\left[ {C > A} \right]A,\mathrm{observed}},\,\mu _{i,A\left[ {C > G} \right]A,\mathrm{observed}},\, \ldots ,\,\mu _{i,T\left[ {T > G} \right]T,\mathrm{observed}}} \right) + \psi$$ with $$\psi = \left( {\mu _{t,A\left[ {C > A} \right]A,\mathrm{observed}} + 1,\,\mu _{t,A\left[ {C > G} \right]A,\mathrm{observed}} + 1,\, \ldots ,\,\mu _{t,T\left[ {T > G} \right]T,\mathrm{observed}} + 1} \right)$$$$\times 23/n_{t,\mathrm{observed}}.$$ These spectra were then normalized to mutation type probabilities using the trinucleotide content on the corresponding chromosomes. In turn, the probabilities were used for rejection sampling of $$n_{i,\mathrm{simulated}}$$ mutations at trinucleotides taken uniformly along the two (diploid) copies of the callable parts of chromosome *i*. The resulting mutation spectra were indistinguishable from the observed spectrum of the sample. During simulation, the algorithm kept track of which allelic positions were mutated and considered them accordingly for biallelic parallel or divergent mutation and back or forward mutation. Simulations were repeated 1,000 times per sample.

In the neighbor resampling model, we resampled without replacement the mutational landscape of a tumor from the empirical mutation distribution, minus the annotated driver SNVs (https://dcc.icgc.org/releases/PCAWG/driver_mutations/). Specifically, in each simulation, we randomly picked 50% of the observed mutations in the original tumor and resampled the other 50% from the pooled SNVs of representative PCAWG tumors. We defined a tumor as representative for the simulation target when it had the same PCAWG histology and similar mutational signature exposures (cosine similarity mutation spectra ≥ 0.9) (ref. ^11^). This can be viewed as sampling one allele from the original tumor and one allele from the corresponding empirical mutation distribution. Note that the approach allowed to simulate biallelic events but not back and forward mutation and could be applied only to tumors with a representative SNV pool at least 0.5 times their total mutation burden. We further excluded all graylisted and non-preferred multi-sample tumors^13^ and 21 prostate cancer cases from the PRAD-CA cohort (prostate adenocarcinoma-Canada), which were suspected of contamination harboring excess low VAF SNV calls in repetitive regions.

Neighbor resampling was also applied to indels, in which case the exact same pipeline described above could be followed using indels instead of SNVs. To identify representative tumors, we used the PCAWG indel signatures (ID 1–17) and their exposures in each of the samples^11^. Microsatellite instability classification of all PCAWG tumors was obtained from Fujimoto et al.^26^.

In all simulations, input mutations being (re)sampled were assumed to represent single events. Since some are in fact biallelic, this may have underestimated the true number of violations.

### Identification of parallel mutations: allele frequencies

Parallel mutation increases the VAF, which can be picked up by comparing it to the B-allele frequency (*BAF*) of local heterozygous SNPs, taking tumor purity and local total copy number (*logR*) into account. We obtained phased *BAF* values and *logR* as an intermediate output of Battenberg copy number calling^4^. Briefly, allele counts at 1000 Genomes phase 3 SNP loci were extracted from the matched tumor and normal BAM files using alleleCount v4.0.0 with a minimal base quality of 20 and mapping quality of 35. Heterozygous SNPs were identified as having $0.1 < BAF < 0.9$ in the matched normal sample and poorly behaving loci were filtered out (Battenberg problematic loci file). Haplotypes were imputed using Beagle 5.0, followed by a piecewise constant fit of the phased tumor *BAF* values and flipping of haplotype blocks with mean $BAF < 0.5$. Total allele counts of tumor and normal samples were converted into *logR* values and corrected for guanine-cytosine-content and replication timing artifacts.

$BAF_{\mathrm{segment}}$ and $logR_{\mathrm{segment}}$ estimates were computed for all PCAWG consensus copy number segments^4^. Allele counts at phased heterozygous SNPs were considered to be generated according to a Beta-binomial model with $V_i\sim\mathrm{Bin}(n_i = V_i + R_i,\,P\sim\mathrm{Beta}\left( {BAF_{\mathrm{segment}} \times \omega ,\,(1 - BAF_{\mathrm{segment}}) \times \omega } \right))$ where *V*_*i*_ and *R*_*i*_ are the observed counts of the major and minor allele of SNP *i*, respectively and *ω* is a sample-specific concentration parameter (that is, a pseudo-coverage of the average segment). For each sample, *ω* was optimized between 50 and 1,000 by computing a two-sided *P* from the Beta-binomial model above for each SNP and ensuring that the robustly fitted slope of a Q–Q plot of these *P* values was equal to 1.

A similar model could subsequently be used to test whether a variant was present on a higher number of copies than the number of copies of the major allele present in the tumor. In pure tumor samples, this would be directly observable since their allele frequency exceeds that of local heterozygous SNPs on the major allele. However, when considering admixed normal cells, the maximal expected allele frequency needed to be corrected for tumor purity and total copy number of the segment as follows:

$$BAF_{\mathrm{somatic}} = BAF_{\mathrm{segment}} - \frac{{1 - \rho }}{{\left( {2\left( {1 - \rho } \right) + \rho {{{\mathrm{{\Psi}}}}}_t} \right)2^{logR_{\mathrm{segment}}}}}$$

with *ρ* and ${{{\mathrm{{\Psi}}}}}_t$ as the PCAWG consensus tumor purity and ploidy, respectively^4^. This amounted to subtracting from the segment *BAF* the contribution of the major allele from admixed normal cells. If $BAF_{\mathrm{somatic}}$ was estimated to be <0.05 for a segment, it was conservatively raised back to $BAF_{\mathrm{segment}}$.

The final Beta-binomial model with $BAF_{\mathrm{somatic}}$ and *ω* then describes the expected allele counts *V*_*i*_ of clonal somatic variants carried on all copies of the major allele. This model was used to perform a one-sided test for the SNVs contained on that copy number segment as $P(V_i \ge v|V_i + R_i,BAF_{\mathrm{somatic}},\omega )$. An independent filtering step required $P(V_i + R_i \ge v|V_i + R_i,BAF_{\mathrm{somatic}},\omega )$ <0.001 to remove sites with low statistical power (that is, low total read counts or $BAF_{\mathrm{somatic}}\sim 1$). *P* values were corrected for multiple testing according to Benjamini and Hochberg and SNVs were considered as potential parallel mutations when *q* ≤ 0.1.

Additional quality checks and filters mitigated potential errors and biases in allele counts, consensus genome segmentation, purity and ploidy: (1) SNVs overlapping a known heterozygous germline SNP in the individual were filtered out; (2) candidate variants were filtered when they resided in a region of common structural variation as listed in nstd186 (National Center for Biotechnology Information (NCBI) Curated Common SVs, all populations from 1000 Genomes; allele frequency ≥ 1%); (3) the *BAF* and *logR* of proximal heterozygous SNPs on either side of a candidate variant should not represent outliers on the segment, which could indicate a missed copy number event. For the *BAF*, we required the two-sided Beta-binomial *P* values of these SNPs, as computed above, to be >0.001 and their combined *P* > 0.01 (Fisher’s method). For the *logR*, identical thresholds apply, with *P* values derived using a two-tailed test assuming a Gaussian distribution with the mean equal to the median segment *logR* and s.d. equal to the median absolute deviation adjusted for asymptotic consistency; (4) candidate parallel mutations with ≥2 heterozygous SNPs within 25 bp were filtered out because these can affect mapping qualities and bias allele counts; (5) SNVs in regions with loss of heterozygosity in the PCAWG consensus copy number were not tested. In males, only the pseudoautosomal regions of X were considered; (6) the robustly fitted slope of a Q–Q plot of the final SNV *P* values should be ≤1, if not, sample purity may have been underestimated and the sample was excluded; (7) candidate variants from tumors where both simulators yielded zero biallelic mutations across 1,000 simulations were excluded.

Further flags were included for quality control but were not used during filtering of the final call set: (1) candidate biallelic hits at T and B cell receptor loci were flagged to assess the impact of V(D)J recombination in infiltrating immune cells on allele frequencies and coverage; (2) for each variant, we checked whether it lifted over from the 1000 Genomes GRCh37 build to a single location on hg38 and required the same reference allele; (3) SNVs were flagged if near an indel (position −10 to +25) in the sample.

### Identification of parallel mutations: variant phasing

Phasing information was obtained for all heterozygous SNP–SNV pairs that were within 700 bp of one another. We counted only read pairs with mapping quality ≥20, base quality ≥25, no hard or soft clipping, which were properly paired, were not flagged as duplicates and did not have a failed vendor quality control flag. We further removed read pairs with indels and those that had ≥2 mismatches in a single read or ≥3 in the whole pair (if the phased variants were spanned by different reads in the pair).

We inferred a parallel mutation when, for a heterozygous SNP–SNV pair, ≥2 reads from each allele of the SNP reported the somatic variant, that is, ≥2 Ref/Alt and ≥2 Alt/Alt reads. In addition, Ref/Alt and Alt/Alt reads each should represent >10% of the total phased reads. To avoid a scenario where, after a gain of the chromosome copy carrying the somatic variant, the in-*cis* allele of the heterozygous SNP is mutated to the in-*trans* allele, we required that the *BAF* of this SNP was not an outlier on the segment by requiring that its two-sided Beta-binomial *P* > 0.001.

While phasing info was sparse, it was less dependent on copy number, purity and coverage than the VAF approach. Phasing to a heterozygous SNP can detect late parallel mutations with multiplicity smaller than the copy number of the major allele, for example, on a segment with copy number 2 + 1 where both parental alleles have 1 copy mutated. Therefore, phasing may be used to evaluate the performance of the VAF approach in a sample. However, both approaches are blind in regions with loss of heterozygosity. Parallel mutations can occur in these contexts when the copy number ≥2 but cannot readily be distinguished from early mutations that have occurred before the duplication.

The precision and recall of the VAF approach were assessed by taking all evaluated phaseable SNVs (that is, SNP–SNV pairs having ≥2 reads each for the SNP Ref and Alt alleles and ≥4 reads reporting the SNV). Precision was calculated as the fraction of VAF-inferred biallelic parallel mutations that were confirmed by phasing. Recall was the fraction of phasing hits picked up through their allele frequencies. Overall performance was reported as the median precision and recall for samples with ≥10,000 phaseable SNVs.

By extrapolating the rate of parallel mutation at phaseable SNVs to all testable SNVs (that is, those passing the quality checks and filters listed above), we estimated the total number of parallel mutations in a sample *i* ($n_{\mathrm{violation},i}$). The estimate and its uncertainty can be described using a Beta-binomial model $n_{\mathrm{violation},i}\sim\mathrm{Bin}(n = n_i,\,P\sim\mathrm{Beta}\left( {n_{\mathrm{phasing,parallel},i} + 0.001,\,n_{\mathrm{phasing,single},i} + 0.001} \right))$ where *n*_*i*_ is the total number of passed SNVs, $n_{\mathrm{phasing,parallel},i}$ the number of phasing-informed biallelic parallel mutations and $n_{phasing,single,i}$ the number of phaseable SNVs without evidence for a parallel hit.

### Birthday problem approximation

The number of infinite sites violations in a sample may be approximated by a variant of the birthday problem, which asks for the probability that at least two people share a birthday in a group of *n* random people. While ignoring intricacies such as mutation types and copy number, it provides a reasonable approximation and straightforward formulation. We started with the probability that mutations A and B hit the same locus: $P\left( {\mathrm{A}} = {\mathrm{B}} \right) = 1/N$ where *N* is the size of the genome. From this we derived the probability that they did not share a locus $P\left( {\mathrm{A}} \ne {\mathrm{B}} \right) = 1 - 1/N$. The probability that A does not hit the same locus as *n* other mutations is then $P\left( {\mathrm{A}} \ne {\mathrm{B}}_1,\, \ldots ,{\mathrm{B}}_{n} \right) = \left( {1 - 1/N} \right)^{n - 1}$. To obtain the expected number of mutations not sharing a locus, this probability was multiplied by the total mutation burden *n*. Finally, the number of infinite sites violations was then $E\left[ {\mathrm {no}}. {\rm{violations}} \right] = n_{\mathrm{violation}} = n - n \times\left( {1 - 1/N} \right)^{n - 1}$. Given that for a human genome $1/N \cong 3^{ - 10} \approx 0$, Taylor approximation yields $n_{\mathrm{violation}} \cong n - n\times\left( {1 - (n - 1)/N} \right) \cong n^2/N$, indicating that the number of infinite sites violations scales with the square of the total mutation burden and the inverse of the genome size.

### Motif enrichment

To assess enrichment of specific motifs at sites with biallelic mutations, we extracted 15-bp sequence contexts (+ strand where C or T was the reference base and − strand otherwise), for all parallel and divergent biallelic mutations. For every biallelic mutation, we sampled 10 mutation type-matched SNVs from the same tumor and extracted their 15-bp contexts as a control set. The Multiple EM for Motif Elicitation suite of tools (STREME and TomTom v.5.3.2) was used to discover sequence motifs enriched in the biallelic set relative to the control set^14,17^. In the case of melanoma, identified motifs were linked to known transcription factor recognition sequences from the HOmo sapiens COmprehensive MOdel COllection (HOCOMOCO) Human v.11 Core collection using TomTom with the Sandelin–Wasserman motif comparison function^18^. *P* values were computed according to STREME and TomTom.

### Gene expression analysis

PCAWG expression data were obtained from the ICGC Data Portal http://dcc.icgc.org/releases/PCAWG/transcriptome/gene_expression/^27^. Briefly, reads were aligned with both TopHat2 v.2.0.12 and STAR v.2.4.0i (two-pass). Read counts for genes were calculated using HTSeq-count v0.11.1 and the GENCODE v.19 annotation. Counts were normalized using fragments per kilobase of transcript per million mapped reads and upper quartile (FPKM-UQ) normalization^27^. The final expression values are an average of the TopHat2 and STAR-based alignments. FPKM-UQ values for genes with recurrent (biallelic) promoter mutations in melanoma were extracted and stratified by promoter mutation status in the tumor (WT, single SNV, biallelic mutation).

To assess whether the single SNVs induced allele-specific expression, we used Rsamtools v3.11 to pile up base counts from the STAR-aligned BAM files at heterozygous germline SNPs. Posterior 95% highest density intervals were computed for the DNA and RNA base counts assuming a uniform Beta(1,1) prior and a binomial likelihood. Nonoverlapping intervals can indicate allele-specific expression.

### Structural analysis

X-ray diffraction and solution nuclear magnetic resonance structures for free B-form DNA, NFAT- or ETS-bound DNA were obtained from the Research Collaboratory for Structural Bioinformatics (RCSB) Protein Data Bank (PDB). C5–C6 interbond distances *d* and torsion angles *η* were extracted using PyMOL v.2.4.0 at the relevant Tp**C** dinucleotide in the ETS and NFAT recognition motifs and at nonterminal Tp**C** dinucleotides in the free B-DNA. When multiple chains were present in a single structure, the average *d* and *η* were used.

### Statistics and reproducibility

No statistical method was used to predetermine sample size. The experiments were not randomized. The investigators were not blinded to allocation during the experiments and outcome assessment. After quality assurance by the PCAWG Consortium, data from 176 of its 2,834 donors were excluded as unusable. Reasons for data exclusions included inadequate coverage, extreme bias in coverage across the genome, evidence for contamination in samples and excessive sequencing errors^13^. These exclusion criteria were predetermined.

In our neighbor resampling simulations, we additionally excluded samples that had been graylisted by the PCAWG Consortium and used only the PCAWG designated representative sample for each patient with multiregion sequencing^13^. In addition, we excluded 21 prostate cancer cases from the PRAD-CA cohort, which were suspect of contamination, harboring excess low VAF SNV calls in repetitive regions of the genome as described in the corresponding Methods section.

### Reporting Summary

Further information on research design is available in the Nature Research Reporting Summary linked to this article.

## Online content

Any methods, additional references, Nature Research reporting summaries, source data, extended data, supplementary information, acknowledgements, peer review information; details of author contributions and competing interests; and statements of data and code availability are available at 10.1038/s41588-021-01005-8.

## Supplementary information

Supplementary Information Supplementary Figs. 1 and 2.

Reporting Summary

Peer Review Information

Supplementary Table 1 Supplementary Tables 1–7.

## Data Availability

The PCAWG dataset is available through the ICGC data portal at https://dcc.icgc.org/pcawg^13^. Further information on accessing the data, including raw read files, can be found at https://docs.icgc.org/pcawg/data/. In accordance with the data access policies of the ICGC and The Cancer Genome Atlas (TCGA) projects, most molecular, clinical and specimen data are in an open tier that does not require access approval. To access information that could potentially identify participants, such as germline alleles and underlying sequencing data, researchers will need to apply to the TCGA Data Access Committee via the database of Genotypes and Phenotypes (dbGaP) (https://dbgap.ncbi.nlm.nih.gov/aa/wga.cgi?page=login) for access to the TCGA portion of the dataset and to the ICGC Data Access Compliance Office (http://icgc.org/daco) for the ICGC portion. In addition, to access somatic SNVs derived from TCGA donors, researchers will also need to obtain dbGaP authorization. Structural data were obtained from the RCSB PDB (https://www.rcsb.org/). The HOCOMOCO Human v.11 Core set was used as the source of known transcription factor recognition sequences (https://hocomoco11.autosome.ru/). NCBI Curated Common SVs are available via the NCBI dbVar at https://www.ncbi.nlm.nih.gov/dbvar/studies/nstd186/. The germline resources of the 1000 Genomes Project and gnomAD were obtained from the International Genome Sample Resource (https://www.internationalgenome.org/) and gnomAD (https://gnomad.broadinstitute.org/), respectively.

## Code availability

The core computational pipelines used by the PCAWG Consortium for alignment, quality control and variant calling are available to the public at https://dockstore.org/search?search=pcawg under the GNU General Public License v.3.0, which allows for reuse and distribution. All custom scripts for simulating, identifying and characterizing biallelic mutations from the PCAWG data are available on GitHub (https://github.com/jdemeul/InfiniteSites). R v.4.0.0 was used for the final analyses. Variant recalling on 195 PCAWG samples was done using GATK v.4.0.8.1, which is available from https://gatk.broadinstitute.org/. STREME and TomTom v.5.3.2 (MEME suite, https://meme-suite.org/) were used for motif enrichment and analysis. PCAWG consensus SNV, MNV, indel and structural variant calling was described by the ICGC/TCGA PCAWG^13^. PCAWG consensus copy number calling was described by Dentro et al.^4^. PCAWG gene expression analysis was reported in Calabrese et al.^27^ and was based on TopHat2 v.2.0.12 and STAR v.2.4.0i alignments. PyMOL v.2.4.0 was used for the structural analyses.
