# Biallelic mutations in cancer genomes reveal local mutational determinants

**DOI:** 10.1038/s41588-021-01005-8

**Published:** 2022-02-10

**Authors:** Jonas Demeulemeester, Stefan C. Dentro, Moritz Gerstung, Peter Van Loo

**Affiliations:** 1 Cancer Genomics Laboratory, The Francis Crick Institute, London NW1 1AT, UK; 2 Department of Human Genetics, KU Leuven, 3000 Leuven, Belgium; 3 European Molecular Biology Laboratory - European Bioinformatics Institute (EMBL-EBI), Hinxton, Cambridgeshire CB10 1SA, UK; 4 Wellcome Sanger Institute, Hinxton, Cambridgeshire CB10 1SA, UK

## Abstract

The infinite sites model of molecular evolution posits that every position in the genome is mutated at most once^1^. By restricting the number of possible mutation histories, haplotypes and alleles, it forms a cornerstone of tumour phylogenetic analysis^2^, and is often implied when calling, phasing and interpreting variants^3,4^ or studying the mutational landscape as a whole^5^. Here we identify 18,495 biallelic mutations, where the same base is mutated independently on both parental copies, in 559 (21%) bulk sequencing samples from the Pan-Cancer Analysis of Whole Genomes (PCAWG) study. Biallelic mutations reveal UV damage hotspots at ETS and NFAT binding sites, and hypermutable motifs in *POLE*-mutant and other cancers. We formulate recommendations for variant calling and provide frameworks to model and detect biallelic mutations. These results highlight the need for accurate models of mutation rates and tumour evolution, as well as their inference from sequencing data.

Recent studies have shown systematic variation in mutation rates across the genome, resulting in specific hotspots^5–7^. In addition, breakdown of the infinite sites assumption at the scale of individual single nucleotide variants (SNVs) was inferred from single-cell tumour sequencing data and flagged as a confounder during phylogenetic reconstruction^8^. In bulk tumour data, population averaging and limited long-range information make it difficult to assess mutational recurrence and its impact on analyses.

In a single diploid lineage, four classes of infinite sites violations may be considered (Figure 1): (i) biallelic parallel and (ii) biallelic divergent, where two alleles independently mutate to the same or different alternate bases, respectively; (iii) monoallelic forward and (iv) monoallelic back, where one variant is mutated to another or back to wild type, respectively. We focus on biallelic mutations, which become problematic when artificially treating genomes as haploid, hypothesising these may be observed directly in bulk tumour genome sequencing data. Loss of variants owing to large-scale genomic deletion does not strictly contradict the infinite sites assumption, yet should be accounted for in cancer genomes^2,8,9^.

To assess the landscape of infinite sites violations, we start with a simulation approach using the PCAWG dataset of 2,658 whole-genome sequenced cancers. We resample a tumour’s observed mutations, preserving mutational signature exposures^10,11^ but otherwise assuming uniform mutability across the callable diploid genome (uniform permutation model; Extended Data Figure 1, Supplementary Table 1). As mutation rates are certainly not uniform and any deviation increases the number of violations^5^, this derives a lower bound of at least one, typically parallel, violation in 147 tumours (5.5%, Figure 2a). A second simulation approach, resampling (without replacement, non-driver) mutations from tumours of the same cancer type with similar mutational signature activities, confirms these observations (neighbour resampling model; Figure 2b, Extended Data Figure 1, Supplementary Table 2). In addition, this approach indicates four microsatellite unstable tumours harbour hundreds of parallel biallelic indels (Extended Data Figure 2). Consistent differences between the simulators, in the number of violations per tumour type, inform on the non-uniformity of the mutational processes, *i*.*e*., a reduced “effective genome size” (akin to the population genetics concept of effective population size; Figure 2c).

Distinct preferences for parallel, divergent, forward and back mutation may be understood from the active mutational processes (Figure 2d). For instance, the dominant mutagenic activity of UV light in cutaneous melanoma (single base substitution signature 7a/b, SBS7a/b) yields almost uniquely C>T substitutions in CC and CT contexts^10,11^, which can only result in accumulation of biallelic parallel mutations. In contrast, in oesophageal adenocarcinoma DO50406, interplay between SBS17a and b^10,11^ results in various substitutions of T in a C **T** T context, generating both parallel and divergent variants. Back and forward mutation occur when the variant allele retains considerable mutability.

We next set out to directly detect biallelic mutations in PCAWG genomes. Parallel mutation increases the variant allele frequency (VAF) and may be distinguished from local copy number gains by comparing the VAF to the allele frequencies of neighbouring heterozygous SNPs, taking tumour purity and copy number into account. Additionally, when proximal to a heterozygous germline variant, read phasing can evidence mutation of both alleles (Figure 3a–b, Extended Data Figure 3, Supplementary Table 3). Without phasing information, we can only detect parallel mutations on more copies than the major allele tumour copy number. Hence, no parallel mutations are called in regions with loss of heterozygosity and late or subclonal events are likely to be underrepresented. Insights into the latter can be glimpsed from multi-sample studies. In a cohort of metastatic prostate cancer with sequencing of matched primary and metastases^12,13^, we discern early clonal (preceding the most recent common ancestor) as well as candidate late and subclonal events (Extended Data Figure 4).

Divergent mutations can be picked up by variant callers but are traditionally filtered out^3^. As neither the PCAWG consensus nor the four contributing pipelines report divergent mutations, we recall mutations with Mutect2 for 195 relevant cases, allowing two alternative alleles (Figure 3c, Supplementary Table 4–5). Overall, recalling identifies a median 96.3% of consensus variants and adds 9.5% novel variants, with 0.04% of the latter contributed by divergent mutations (Supplementary Figure 1). For 90% of divergent mutations, one of the alternate alleles is reported in the PCAWG consensus.

In total, we identify 5,330 divergent mutations, 12,937 parallel SNVs and 14 dinucleotide variants in 559 (21%) PCAWG samples (Supplementary Table 3–5). Parallel mutations confirmed by phasing are found in tumours with as few as 8,892 SNVs while divergent mutations are repeatedly identified in oesophageal adenocarcinomas with 20,000-30,000 SNVs (Extended Data Figure 5). On the other end of the spectrum, phasing indicates that two ultra-hypermutated colorectal adenocarcinomas each boast around 8,000 parallel and 1,700 divergent mutations.

Biallelic mutations carry a footprint determined by, but distinct from, the overall mutational profile. For example, as parallel mutations require two independent identical hits, they show a mutation spectrum similar to the square of that of SNVs (Figure 4a–b). Indeed, the observed biallelic mutations are better explained by the simulated violation spectra than the overall mutation spectra (*p* = 2.83x10^-4^ and 1.35x10^-8^ for parallel and divergent, respectively, median simulated–observed cosine similarities 0.968 and 0.944, Mann–Whitney *U*, samples with ≥ 10 violations). This further supports the accuracy of our biallelic mutation calls, excluding major contributions from sequencing and alignment artefacts, germline variants, focal tandem duplicator phenotypes, precursor lesions or somatic gene conversion.

While the uniform permutation model underestimates, neighbour resampling accurately predicts the number of biallelic mutations (Figure 4c, Extended Data Figure 6). Resampling mutation burdens and tumour types with the confirmed model demonstrates how biallelic mutations are proportional to the square of the mutation burden (*m^2^*, Figure 4d). The coefficient per tumour type (*C_type_*) scales the callable genome size (*N*) and provides straightforward estimation of the number of violations as *C_type_ m^2^/_N_* (Figure 4d–e).

Biallelic mutations are not associated with somatic rearrangements (*p_adj_* ≥ 0.31; Mann-Whitney *U*-test, Benjamini–Hochberg) but occur at loci with a higher mutation rate (Extended Data Figure 7), some of which harbour recurrent biallelic events (Figure 5a). The promoter of *RPL18A* shows three parallel, one divergent, and nine single mutations at chr19:17,970,682, all in melanoma (12% total, Extended Data Figure 8)^14^. Motif enrichment at loci with biallelic *vs*. trinucleotide-matched monoallelic hits in melanoma reveals enrichment of Y **C** TT **C** CGG and WTTT **C** C motifs (Figure 5a–b)^14^. Y **C** TT **C** CGG motifs are recognised by E26 transformation-specific (ETS) transcription factor family members. Binding increases their sensitivity to UV damage due to perturbation of the Tp **C** C5–C6 interbond distance *d* and torsion angle *η*, favouring cyclobutane pyrimidine dimer formation (Figure 5c-d)^15,16^. The WTTT **C** C motif matches the recognition sequence for Nuclear factor of activated T-cells (NFAT) transcription factors^17,18^. Analysis of crystal structures of NFATc1–4 bound to DNA indicates that binding induces similar, less outspoken, Tp **C** conformational changes which may explain its increased mutability (Figure 5d, Supplementary Table 6). While we cannot formally exclude selection as a contributor to these recurrent mutations, no effects on total or allele-specific expression of genes with biallelic promoter mutations could be observed (Extended Data Figure 9).

Similar analysis in colorectal adenocarcinoma reveals special cases of the SBS10a/b and SBS28 sequence contexts, which are associated with Pol *ε* exonuclease domain mutations (Figure 5a,e)^10,11,19^. AWTT **C** T and TT **C** GAA for SBS10 and AAA **TT** T for SBS28 all carry extra adenosine and thymine bases surrounding the regular trinucleotide context of the mutated C and T, respectively. Likewise, AT-rich sequences surrounding the canonical SBS17 C **T** T context render some loci hypermutable in oesophageal and stomach adenocarcinomas (AAAC **T** TA motif; Figure 5a,e). These preferences have also been observed in the recent extension from tri- to pentanucleotide signatures^11^. It is unclear however how these additional bases increase local mutability. Last, it is worth highlighting recurrent (biallelic) mutation at chr6:142,706,206, in an intron of *ADGRG6* (Figure 5a). The CTCTTTGTAT-GTT **C**-ATACAAAGAG palindrome may adopt a hairpin structure, exposing the hypermutable C in a 4bp loop and rendering it susceptible to APOBEC3A deamination^7^.

Biallelic hits provide insights beyond mutational processes. The rate of biallelic mutation is proportional to that of parallel mutation between clones and increases with both the number of lineages considered and total mutation burden (Supplementary Figure 2). When constructing phylogenies from ever more exhaustive multi-sample or single-cell data^20,21^, biallelic mutations provide an estimate for the number of parallel events.

Using single-sample bulk sequencing to establish evolutionary relationships between subclones is challenging^4,22^. Under the infinite sites assumption, one can examine rare pairs of phaseable SNVs in regions without copy number gains^4,23^. Specifically, a pattern where one SNV is only found on a subset of the reads reporting the other evidences a linear relationship (Extended Data Figure 10a). In PCAWG melanomas, however, a median 67% of these pairs in diploid regions reflect phylogenetically uninformative biallelic parallel mutations (Extended Data Figure 10b). To avoid biasing phylogenies, biallelic SNVs should be filtered by restricting analyses to haploid regions or scrutinising the VAF and the likelihood of biallelic mutation in the sample^4^. SNV clustering approaches, which rely on the infinite sites assumption for subclonal reconstruction and assignment of each variant to a specific lineage, may pick up “superclonal clusters” of biallelic parallel mutations, but are otherwise expected to remain robust at the levels identified here (Extended Data Figure 10c)^22^.

Phasing is also used to boost the accuracy of variant callers for single molecule sequencing data^24^. As with multi-allelic variants, relaxation of the set of allowed haplotypes will need to be considered to capture the full extent of somatic variation. Indeed, while only 2.8% of biallelic hits fall within or near exons, we identify 8 candidate biallelic driver events. Parallel nonsense mutations in tumour suppressors *ASXL2* and *CDKN2A*, and divergent events in *ERBB4*, suggest that in rare cases, biallelic mutations are selected for (Extended Data Figure 10d, Supplementary Table 7).

Taken together, we identify 18,495 biallelic mutations in 21% of PCAWG cases, demonstrating how the infinite sites assumption breaks down at the bulk level for a considerable fraction of tumours. By extension, the model becomes untenable in most, if not all, tumours at the multi-sample or single cell level. If not correctly identified, biallelic mutations confound variant interpretation, ranging from driver inference to subclonal clustering and timing analyses, as well as phylogenetic inference. Nevertheless, at-scale detection of biallelic mutations affords an intimate look at the mutational processes operative in cells, such as hotspots, hypermutable motifs and the molecular mechanisms of DNA damage and repair.

## Methods

### Singe Nucleotide Variant calling

PCAWG consensus single and multi-nucleotide variant calls are obtained from http://dcc.icgc.org/releases/PCAWG/consensus_snv_indel/. Briefly, these calls were constructed according to a “2+ out of 4” strategy, where calls made by at least two callers (the three Broad, EMBL/DKFZ, and Sanger core PCAWG pipelines, plus MuSE v1.0) were selected as consensus calls^13^. Post-merging, these calls were subject to further quality control including filtering against oxidative artefacts (OxoG), alignment (BWA *vs*. BLAT), or strand biases resulting from different artefact-causing processes, as well as checks for tumour-in-normal and sample cross-contamination. Crucially, care was taken to avoid “bleed-through” of germline variants into the somatic mutation calls. Specifically, absence from the Broad panel-of-normals based on 2,450 PCAWG samples and a higher read coverage (≥19 reads with at most one read reporting the alternate allele) in the matched normal sample were required to call a somatic mutation at one of the >14M common (>1%) polymorphic loci of the 1000 genomes project. SNVs that overlapped a germline SNV or indel call in the matched normal were also removed. Sensitivity and precision of the final consensus somatic SNV calls were 95% (90% CI [88, 98]) and 95% [71, 99], respectively, as evaluated by targeted deep-sequencing validation^13^. Of note, 18 biallelic parallel mutations identified here were covered by the PCAWG validation effort with 17 passing and one not being observed.

To identify biallelic divergent variants, which are filtered out in PCAWG, we recalled variants on 195 non-graylisted^13^ PCAWG tumour-normal pairs (that do not show any tumour-in-normal contamination) where we might reasonably expect to find such mutations according to our uniform permutation simulations. Included also, as an internal control, are all other samples from MELA-AU cohort which meet these criteria but in which we do not expect biallelic divergent mutations. SNVs and indels are called using Mutect2 (GATK v4.0.8.1) on the base quality score-recalibrated PCAWG bam files and filtered following best practices^25^. The Genome Aggregation Database (gnomAD) was provided as a germline resource and an additional panel of normals was derived from all matched normal cases. To prevent filtering of biallelic variants, FilterMutectCalls is run with *--max-alt-allele-count 2*. Additional filtering against germline SNPs was done by requiring a posterior probability for the alternative allele to be germline (P_GERMLINE) < -1 for both of the alternate alleles and requiring a minimal depth of 19 high quality reads (mapping quality ≥ 35 and base quality ≥ 20) in the matched normal sample.

### Consensus copy number, purity and ploidy

PCAWG consensus copy number, tumour purity, and ploidy were obtained from ^4,13^ http://dcc.icgc.org/releases/PCAWG/consensus_cnv/. Briefly, each cancer’s genome was first segmented into regions of constant copy number using six individual copy number callers: ABSOLUTE, ACEseq, Battenberg, cloneHD, JaBbA and Sclust, run as detailed in Dentro *et al*.^4^. Consensus segment breakpoints were determined from the PCAWG consensus structural variants (http://dcc.icgc.org/releases/PCAWG/consensus_sv/) complemented with high-confidence breakpoints identified by several of the copy number callers. The six callers were then re-run, enforcing this consensus segmentation as well as separately established consensus tumour ploidy, which was typically obtained by resolving disagreement on whether a whole genome duplication had occurred by an expert panel^4^. The allele-specific copy number calls were combined by looking, for each segment, at the agreement in major and minor allele copy number states between the callers. Lastly, consensus was obtained on tumour purity by combining the calls from the six copy number callers with those from subclonal architecture reconstruction methods that leverage SNV data: CliP, CTPsingle, PhyloWGS, cloneHD and Ccube, as detailed in Dentro *et al*.^4^. This multi-tiered approach yielded a purity for every tumour and a quality tiered copy number for every consensus segment.

### Simulating infinite sites violations

To estimate the number of infinite sites violations in tumours, we developed two distinct simulation approaches leveraging the PCAWG consensus SNV calls.

Our uniform permutation model resamples the observed SNVs in a tumour uniformly across the callable regions of the chromosomes, according to the observed trinucleotide-based mutational spectrum. A single simulation proceeds as follows. First, the total mutational load ^*n*^t,sim is resampled from a gamma-Poisson mixture where the Poisson rate parameter *λ* ~ Gamma with mode equal to the observed mutational load *n_t,obs_* and a standard deviation *σ* = 0.05 × *n_t,obs_*. That is: *n_t,sim_* ~ *Poisson*(*λ* ~ *Gamma*(*r*, *β*)) where the rate of the Gamma distribution $\;r=n_{t,obs}+\sqrt{n_{t,obs}^{2}+2\sigma^{2}}/2\sigma^{2}$ and the shape *β* = 1 + *n_t,obs_* × *r*.

Mimicking the observed distribution, these mutations are then divided across the chromosomes according to a Dirichlet-multinomial model with *n_t,sim_* trials and parameter vector ***α*** where α_i_ is equal to 1 + the total mutational burden on chromosome *i*. That is: ***n* *_sim_*** ~ *Mult* (*n_t,sim_*,*π* ~ *Dir*(***α***)) with ***α*** = (*n_1,obs_,n_2,obs_*, …,*n_x,obs_*) + **1**. Next, mutation spectra per chromosome (*π_i_*) are sampled from a Dirichlet distribution with parameter vector *μ_i_* where *μ_i,j_* is equal to a pseudocount *ψj* derived from the overall mutational spectrum plus the observed number of mutations of type *j* on chromosome *i*. That is: ***π_i_*** ~ *Dir*(***μ_i_***) with $\mu_{i}=\left(\mu_{i,A[C>A]A,obs},\mu_{i,A[C>G]A,obs},\ldots ,\mu_{i,T[T>G]T,obs}\right)+\psi$ with $\psi =\left(\mu_{t,A[C>A]A,obs}+1,\mu_{t,A[C>G]A,obs}+1,\ldots ,\mu_{t,T[T>G]T,obs}+1\right)\times 23/n_{t,obs}$ . These spectra are then normalised to mutation type probabilities using the trinucleotide content on the corresponding chromosomes. In turn, the probabilities are used for rejection sampling of *n_i,sim_* mutations at trinucleotides taken uniformly along the two (diploid) copies of the callable parts of chromosome *i*. The resulting mutation spectra are indistinguishable from the observed spectrum of the sample. During simulation, the algorithm keeps track of which allelic positions have been mutated and considers them accordingly for biallelic parallel or divergent mutation and back or forward mutation. Simulations are repeated 1,000 times per sample.

In the neighbour resampling model, we resample without replacement the mutational landscape of a tumour from the empirical mutation distribution, minus the annotated driver SNVs (https://dcc.icgc.org/releases/PCAWG/driver_mutations/). Specifically, in each simulation, we randomly pick 50% of the observed mutations in the original tumour and resample the other 50% from the pooled SNVs of representative PCAWG tumours. We define a tumour as representative for the simulation target when it has the same PCAWG histology and similar mutational signature exposures (cosine similarity mutation spectra ≥ 0.9)^11^. This can be viewed as sampling one allele from the original tumour and one allele from the corresponding empirical mutation distribution. Note that the approach allows to simulate biallelic events but not back and forward mutation and can be applied only to tumours with a representative SNV pool at least 0.5 times their total mutation burden. We further exclude all graylisted and non-preferred multi-sample tumours^13^ as well as 21 prostate cancer cases from the PRAD-CA cohort which were suspected of contamination harbouring excess low VAF SNV calls in repetitive regions.

Neighbour resampling was also applied to indels, in which case the exact same pipeline described above could be followed, using indels instead of SNVs. To identify representative tumours, we used the PCAWG indel signatures (ID1–17) and their exposures in each of the samples^11^. Microsatellite instability classification of all PCAWG tumours was obtained from Fujimoto *et al*.^26^.

In all simulations, input mutations being (re)sampled are assumed to represent single events. As some are in fact biallelic, this may underestimate the true number of violations.

### Identification of parallel mutations – allele frequencies

Parallel mutation increases the variant allele frequency, which can be picked up by comparing it to the B-allele frequency (*BAF*) of local heterozygous SNPs, taking tumour purity and local total copy number (logR) into account. We obtain phased *BAF* values and logR as an intermediate output of Battenberg copy number calling^4^. Briefly, allele counts at 1,000 Genomes v3 SNP loci are extracted from the matched tumour and normal bam files using alleleCount with a minimal base quality of 20 and mapping quality of 35. Heterozygous SNPs are identified as having 0.1 < *BAF* < 0.9 in the matched normal sample and poorly behaving loci are filtered out (Battenberg problematic loci file). Haplotypes are imputed using Beagle5, followed by a piecewise constant fit of the phased tumour *BAF* values and flipping of haplotype blocks with mean *BAF* < 0.5. Total allele counts of tumour and normal are converted into LogR values and corrected for GC-content and replication timing artefacts.

*BAF_seg_* and *logR_seg_* estimates are computed for all PCAWG consensus copy number segments^4^. Allele counts at phased heterozygous SNPs are considered to be generated according to a beta-binomial model with *V_i_* ~ *Bin*(*n_i_* = *V_i_* + *R_i_*, *p* ~ *Beta*(*BAF_seg_* × *ω*,(1 − *BAF_se_g*) × *ω*)) where *V_i_* and *R_i_* are, respectively, the observed counts of the major and minor allele of SNP *i*, and *ω* is a sample-specific concentration parameter (*i.e*. a pseudo-coverage of the average segment). For each sample, *ω* is optimised between 50 and 1000, by computing for each SNP a two-sided *P*-value from the beta-binomial model above and ensuring the robustly fitted slope of a QQ-plot of these *P*-values is equal to 1.

A similar model can subsequently be used to test whether a variant is present on a higher number of copies than the number of copies of the major allele present in the tumour. In pure tumour samples, this would be directly observable as their allele frequency exceeds that of local heterozygous SNPs on the major allele. Considering admixed normal cells, however, the maximal expected allele frequency needs to be corrected for tumour purity and total copy number of the segment as follows:

$$BAF_{\mathrm{som}}=BAF_{\mathrm{seg}}-\frac{1-\rho }{\left(2(1-\rho )+\rho {\text{Ψ}}_{t}\right)2^{\log R_{\mathrm{seg}}}}$$

with *ρ* and Ψ_*t*_, the PCAWG consensus tumour purity and ploidy, respectively^4^. This amounts to subtracting from the segment *BAF* the contribution of the major allele from admixed normal cells. If *BAF_som_* is estimated to be < 0.05 for a segment, it is conservatively raised back to *BAF_seg_*.

The final beta-binomial model with *BAF_som_* and *ω* then describes the expected allele counts *V_i_* of clonal somatic variants carried on all copies of the major allele. This model is used to perform a one-sided test for the SNVs contained on that copy number segment as *P*(*V_i_* ≥ *υ* | *V_i_* + *R_i_,BAF_som_, ω*). An independent filtering step requires *P(V_i_ + R_i_ ≥ υ* | *V_i_ + R_i_,BAF_som_, ω*) < 0.001 to remove sites with low statistical power (*i.e*., low total read counts or *BAF_som_ ~* 1). *P*-values are corrected for multiple testing according to Benjamini–Hochberg and SNVs are considered as potential parallel mutations when *q* ≤ 0.1.

Additional quality checks and filters mitigate potential errors and biases in allele counts, consensus genome segmentation, purity and ploidy. (i) SNVs overlapping a known heterozygous germline SNP in the individual are filtered out. (ii) Candidate variants are filtered when they reside in a region of common structural variation as listed in nstd186 (NCBI Curated Common SVs – all populations from 1,000 Genomes; allele frequency ≥ 0.01). (iii) *BAF* and *logR* of proximal heterozygous SNPs on either side of a candidate variant should not represent outliers on the segment, which could indicate a missed copy number event. For the *BAF*, we require the two-sided beta-binomial *P*-values of these SNPs, as computed above, to be > 0.001 and their combined *P*-value > 0.01 (Fisher’s method). For the *logR*, identical thresholds apply, with *P*-values derived using a two-tailed test assuming a Gaussian distribution with mean equal to the median segment *logR* and standard deviation the median absolute deviation adjusted for asymptotic consistency. (iv) Candidate parallel mutations with ≥ 2 heterozygous SNPs within 25 bp are filtered out as these can affect mapping qualities and bias allele counts. (v) SNVs in regions with loss of heterozygosity in the PCAWG consensus copy number are not tested. In males, only the pseudoautosomal regions of X are considered. (vi) The robustly fitted slope of a QQ-plot of the final SNV *P*-values should be ≤ 1, if not, sample purity may have been underestimated and the sample is excluded. (vii) Candidate variants from tumours in which both simulators yield zero biallelic mutations across 1,000 simulations were excluded.

Further flags were included for quality control, but were not used during filtering of the final call set. (i) Candidate biallelic hits at T- and B-cell receptor loci are flagged to assess the impact of V(D)J recombination in infiltrating immune cells on allele frequencies and coverage. (ii) For each variant, we checked whether it lifted over from the 1,000 Genomes GRCh37 build to a single location on hg38 and required the same reference allele. (iii) SNVs were flagged if near an indel (position -10 to +25) in the sample.

### Identification of parallel mutations – variant phasing

Phasing information is obtained for all heterozygous SNP–SNV pairs that are within 700bp of one another. We count only read pairs with mapping quality ≥ 20, base quality ≥ 25, no hard or soft-clipping, that are properly paired, are not flagged as duplicates and do not have a failed vendor quality control flag. We further remove read pairs with indels and those that have ≥ 2 mismatches in a single read or ≥ 3 in the whole pair (if the phased variants are spanned by different reads in the pair).

We infer a parallel mutation when, for a heterozygous SNP–SNV pair, ≥ 2 reads from each allele of the SNP report the somatic variant, *i.e*., ≥ 2 Ref-Alt and ≥ 2 Alt-Alt reads. In addition, Ref-Alt and Alt-Alt reads each should represent > 10% of the total phased reads. To avoid a scenario where, after a gain of the chromosome copy carrying the somatic variant, the in-*cis* allele of the heterozygous SNP is mutated to in-*trans* allele, we require that the BAF of this SNP is not an outlier on the segment by requiring that its two-sided beta-binomial *P*-value > 0.001.

While phasing info is sparse, it is less dependent on copy number, purity and coverage than the VAF approach. Phasing to a heterozygous SNP can detect late parallel mutations with multiplicity smaller than the copy number of the major allele, *e.g*., on a segment with copy number 2+1 where both parental alleles have one copy mutated. Phasing may therefore be used to evaluate the performance of the VAF approach in a sample. However, both approaches are blind in regions with loss of heterozygosity. Parallel mutations can occur in these contexts when the copy number ≥ 2 but cannot readily be distinguished from early mutations which have occurred before the duplication.

Precision and recall of the VAF approach are assessed by taking all evaluated phaseable SNVs (*i.e*. SNP-SNV pairs having ≥ 2 reads each for the SNP Ref and Alt alleles and ≥ 4 reads reporting the SNV). Precision is calculated as the fraction of VAF-inferred biallelic parallel mutations which are confirmed by phasing. Recall is the fraction of phasing hits picked up through their allele frequencies. Overall performance is reported as the median precision and recall for samples with ≥ 10,000 phaseable SNVs.

By extrapolating the rate of parallel mutation at phaseable SNVs to all testable SNVs (*i.e*. those passing the quality checks and filters listed above), we estimate the total number of parallel mutations in a sample *i* (*n_viol ,i_*). The estimate and its uncertainty can be described using a beta-binomial model *n_viol ,i_* ~ *Bin*(*n* =*n_i_,P* ~ *Beta*(*n_phas,par ,i_* + 0001,*n_phas,single,i_* + 0.001)) where *n_i_* is the total number of passed SNVs, *n_phas,par, i_* the number of phasing-informed biallelic parallel mutations and *n_phas,singel ,i_* the number of phaseable SNVs without evidence for a parallel hit.

### Birthday problem approximation

The number of infinite sites violations in a sample may be approximated by a variant of the birthday problem, which asks for the probability that at least two people share a birthday in a group of *N* random people. While ignoring intricacies such as mutation types and copy number, it provides a reasonable approximation and straightforward formulation. We start with the probability that mutation A and B hit the same locus: *P*(*A* = *B*) = ^1^/_*N*_ where *N* is the size of the genome. From this we derive the probability they do not share a locus *P*(*A* ≠ *B*) = 1 − 1/*N*. The probability *A* does not hit the same locus as *n* other mutations is then *P*(*A* ≠ *B*_1_, …,*B_n_*) = (1 − ^1^/_*N*_)^*n*–1^. To obtain the expected number of mutations not sharing a locus, this probability is multiplied by the total mutation burden *n*. Finally, the number of infinite sites violations is then *E*[*#violations*] = *n_viol_* = (1 − ^1^/_*N*_)^*n*−1^. Given that for a human genome ^1^/_*N*_ ≌ 3^–10^ ≈ 0, Taylor approximation yields *n_viol_* = *n* − _2_ *n*.(1 − (*n* − 1)/*N*) ≌^*n* ^2^^/_*N*_, indicating that the number of infinite sites violations scales with the square of the total mutation burden and the inverse of the genome size.

### Motif enrichment

To assess enrichment of specific motifs at sites with biallelic mutations, we extracted 15bp sequence contexts (+ strand where C or T is the reference base and - strand otherwise), for all parallel and divergent biallelic mutations. For every biallelic mutation, we sampled 10 mutation type-matched SNVs from the same tumour and extracted their 15bp contexts as a control set. The Multiple EM for Motif Elicitation suite of tools (STREME and TomTom; v5.3.2) was used to discover sequence motifs enriched in the biallelic relative to the control set^14,17^. In the case of melanoma, identified motifs were linked to known transcription factor recognition sequences from the HOCOMOCO Human v11 Core collection using TomTom 18 with the Sandelin-Wasserman motif comparison function^18^. *P*-values were computed according to STREME and TomTom.

### Gene expression analysis

PCAWG expression data was obtained from http://dcc.icgc.org/releases/PCAWG/transcriptome/gene_expression/ ^27^. Briefly, reads were aligned with both TopHat2 (v.2.0.12) and STAR (v.2.4.0i, two-pass). Read counts for genes were calculated using htseq-count and the GENCODE v19 annotation. Counts were normalized using Fragments Per Kilobase of transcript per Million mapped reads and upper quartile normalization (FPKM-UQ)^27^. The final expression values are an average of the TopHat2 and STAR-based alignments. FPKM-UQ values for genes with recurrent (biallelic) promoter mutations in melanoma were extracted and stratified by promoter mutation status in the tumour (wild type, single SNV, biallelic mutation).

To assess whether the single SNVs induce allele-specific expression, we used RSamtools to pile up base counts from the STAR-aligned bam files at heterozygous germline SNPs. Posterior 95% highest density intervals were computed for the DNA and RNA base counts assuming a uniform *Beta*(1,1) prior and a binomial likelihood. Non-overlapping intervals can indicate allele-specific expression.

### Structural analysis

X-ray diffraction and solution Nuclear Magnetic Resonance structures for free B-form DNA, NFAT- or ETS-bound DNA were obtained from the RCSB Protein Data Bank. C5-C6 interbond distances *d* and torsion angles *η* were extracted using PyMOL v2.4.0 at the relevant TpC dinucleotide in the ETS and NFAT recognition motifs and at non-terminal TpC dinucleotides in the free B-DNA. When multiple chains were present in a single structure, the average *d* and *η* were used.

## 1 Extended Data

**Extended Data Fig. 1:**
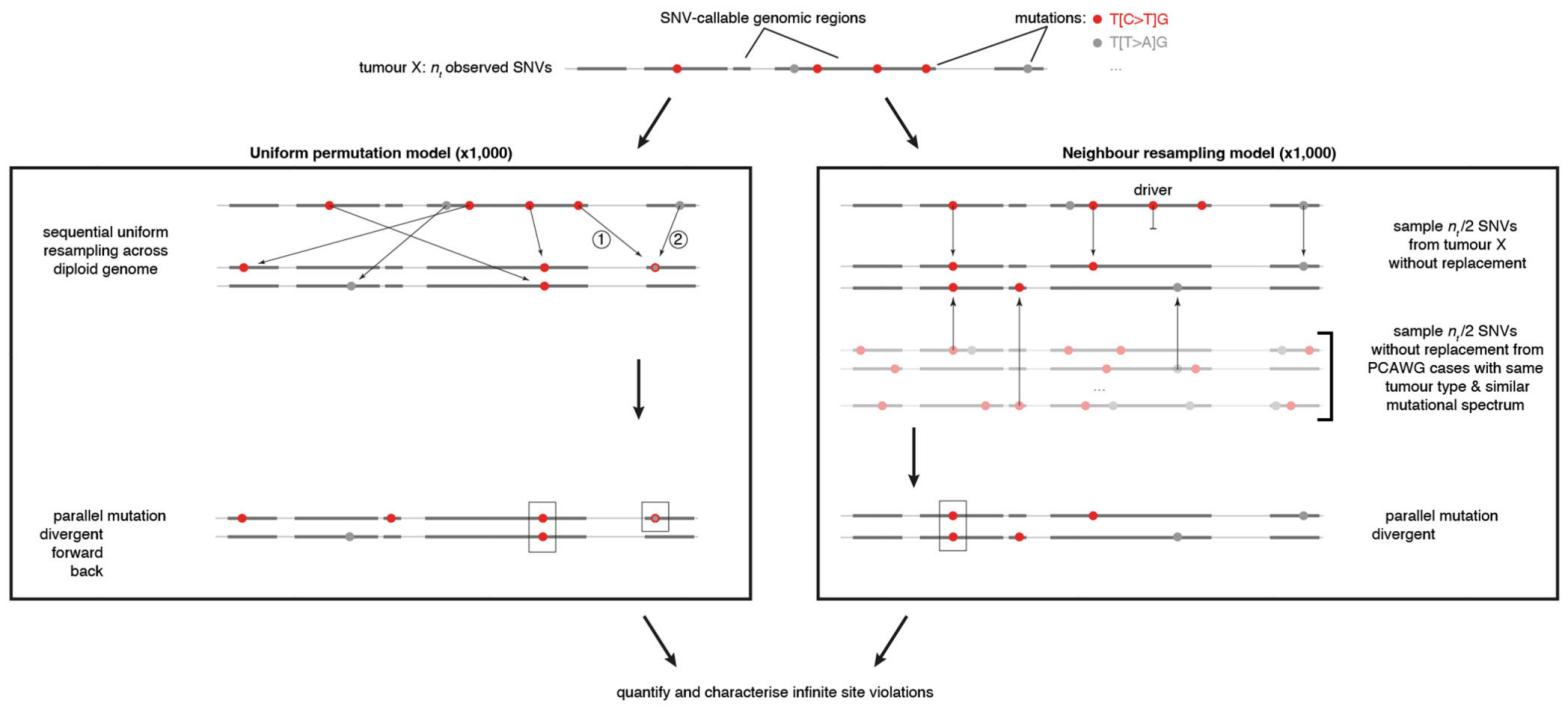
Simulation approaches for infinite sites violations. Schematic overview of the uniform permutation (left) and neighbour resampling (right) approaches to assess the number and type of infinite sites violations expected in a tumour. Numbers in the uniform permutation panel highlight the sequential nature of the sampling, which keeps track of mutated positions to consider them accordingly for biallelic, forward, and back mutation. Note that the neighbour resampling model excludes all PCAWG annotated driver mutations and allows simulation of biallelic events only.

**Extended Data Fig. 2:**
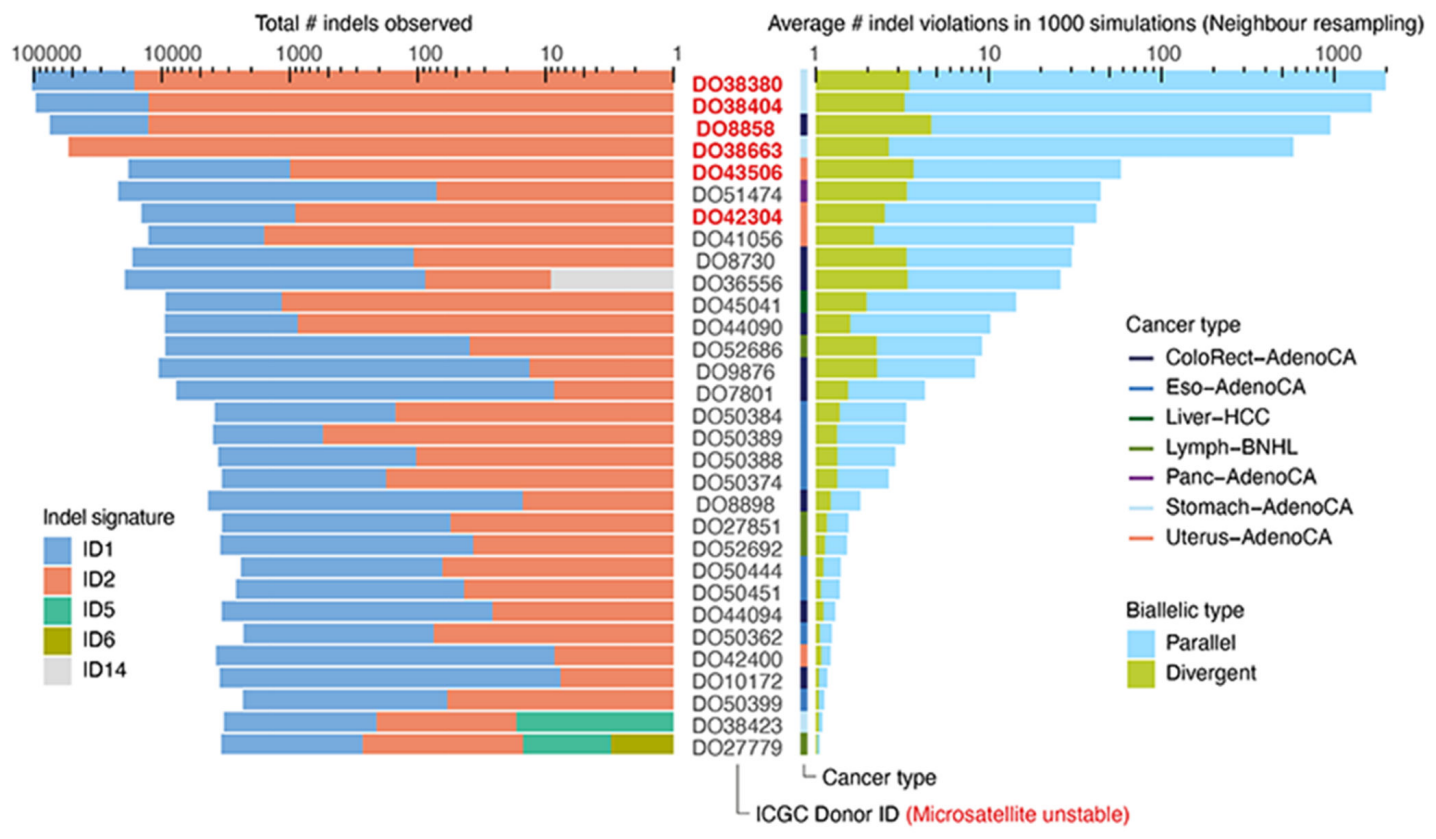
Biallelic indels are expected in a subset of microsatellite unstable tumours. Bar plots of the observed indel burden and signature (left) and the expected biallelic indels according to the neighbour resampling model (right). Bar height indicates total numbers and coloured subdivisions represent fractions contributed by each indel signature (left) or biallelic indel type (right). Only PCAWG tumours with ≥1 expected biallelic indel are shown. Four microsatellite unstable tumours are predicted to boast several hundreds to over one thousand, mostly parallel, biallelic indels. These mainly originate from indel signatures 1 and 2, likely reflecting slippage during DNA replication and subsequent 1bp T (or A) insertion and deletion in thymine (adenosine) mononucleotide repeats, respectively.

**Extended Data Fig. 3:**
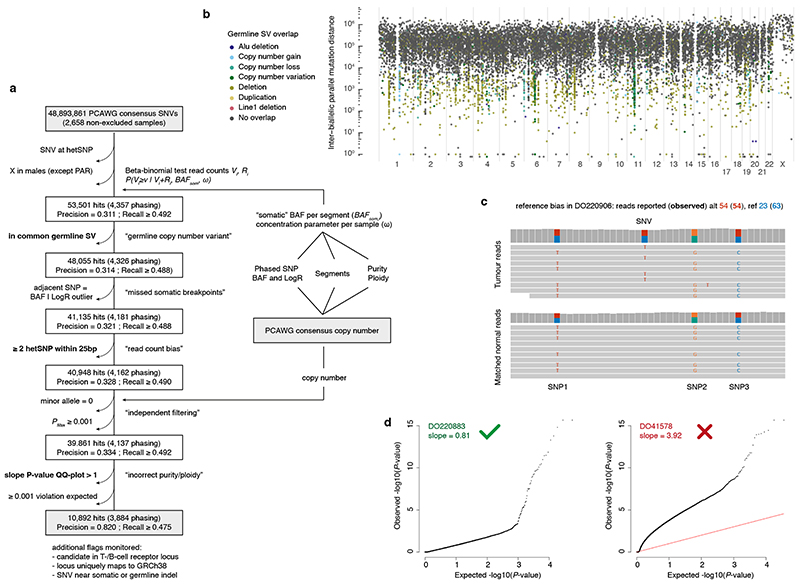
Detection of biallelic parallel mutations by allele frequency. (**a**) Flow chart showing the filtering steps, phasing-based estimates of precision and lower bound recall, as well as the input and output data for our pipeline to detect biallelic parallel mutations in PCAWG based on variant allele frequencies (VAF). Three filtering steps highlighted in bold are further illustrated in panels (b-d). (**b**) Rainfall plot of all biallelic parallel hits obtained after omitting the germline SV filter. Streaks of coloured dots indicate a clustering of hits in regions with common germline structural variants. While demonstrating the ability of the pipeline to detect VAF outliers, these hits are poorly supported by phasing data and likely represent single somatic SNVs in the context of a heterozygous germline deletion. (**c**) Example of reference bias in the PCAWG consensus SNV read counts. Reads carrying the somatic variant contain alternate germline alleles at three proximal positions, resulting in an underreporting of the number of wild type reads and an overestimation of the VAF. (**d**) Diagnostic QQ-plots of the unadjusted one-sided betabinomial read count test *P*-values for two samples (Methods). DO41578 *P*-values are overinflated (slope > 1), hinting at consensus purity/ploidy errors, and the sample is excluded as a result.

**Extended Data Fig. 4:**
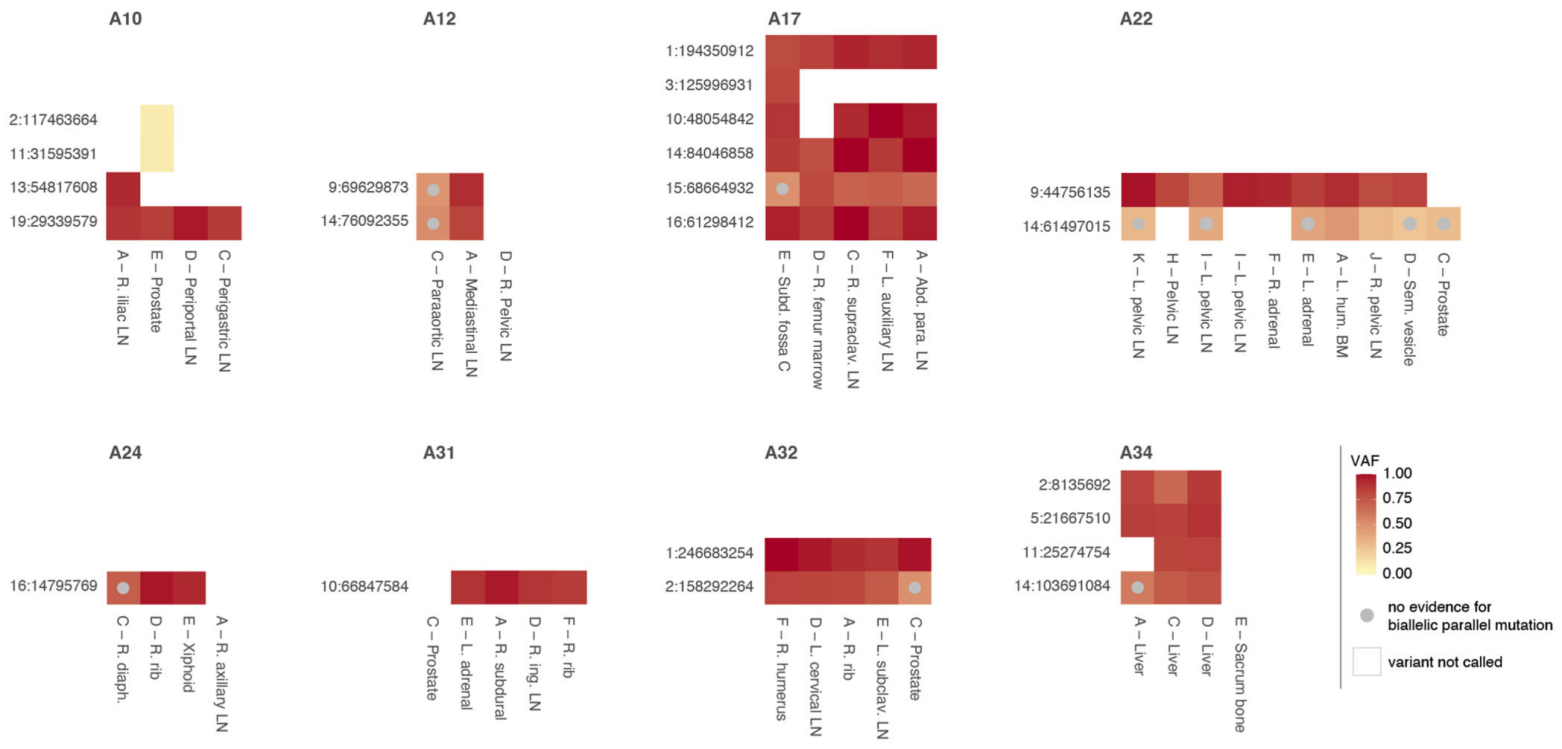
Biallelic parallel mutation during metastatic prostate cancer evolution. Heatmap showing allele frequencies of variants found to be biallelic in at least one sample of eight prostate cancers with sequencing of matched primary and metastases (A10-A34, different sites indicated as in Gundem et al.^12^). Early clonal biallelic mutations are detected in all samples of a patient (*e.g.,* A10 chr19:29,339,579), while late clonal and subclonal ones show no evidence of being biallelic in some samples (beta-binomial p-value > 0.05 and no discordant phasing to a heterozygous germline SNP) or are detected in only a subset of samples (*e.g.,* A22 chr14:61,497,015 and A10 chr2:117,463,664, respectively).

**Extended Data Fig. 5:**
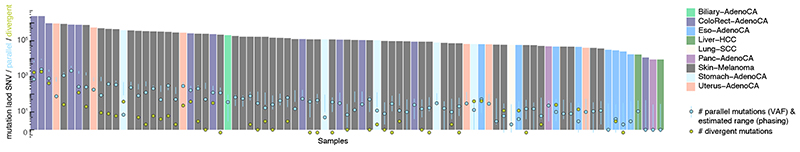
Landscape of biallelic mutations across PCAWG. Number of observed parallel (red) and divergent (blue) mutations plotted in context of the total SNV burden for 84 PCAWG samples with ≥ 1 phasing-confirmed VAF hit. The range of parallel mutations expected purely from SNV-SNP phasing is also indicated (95% confidence interval, red vertical bars) as this approach is less sensitive to purity and copy number state than the VAF-based analysis. Samples for which the number of divergent mutations is not shown were not considered for Mutect2 recalling.

**Extended Data Fig. 6:**
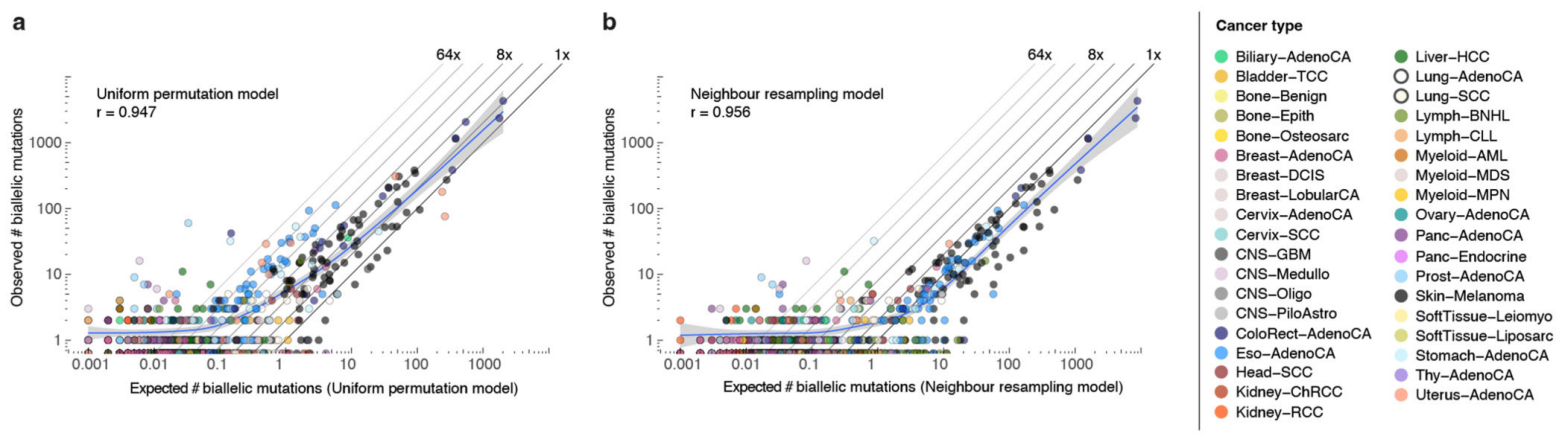
Comparison between observed and simulated biallelic mutations. (**a,b**) Scatterplots of the observed *vs.* expected number of biallelic mutations (parallel + divergent) for all PCAWG tumours using the uniform permutation (**a**) and neighbour resampling models (**b**). The Pearson correlation and a spline regression fit with 95% confidence interval (shaded grey) are shown.

**Extended Data Fig. 7:**
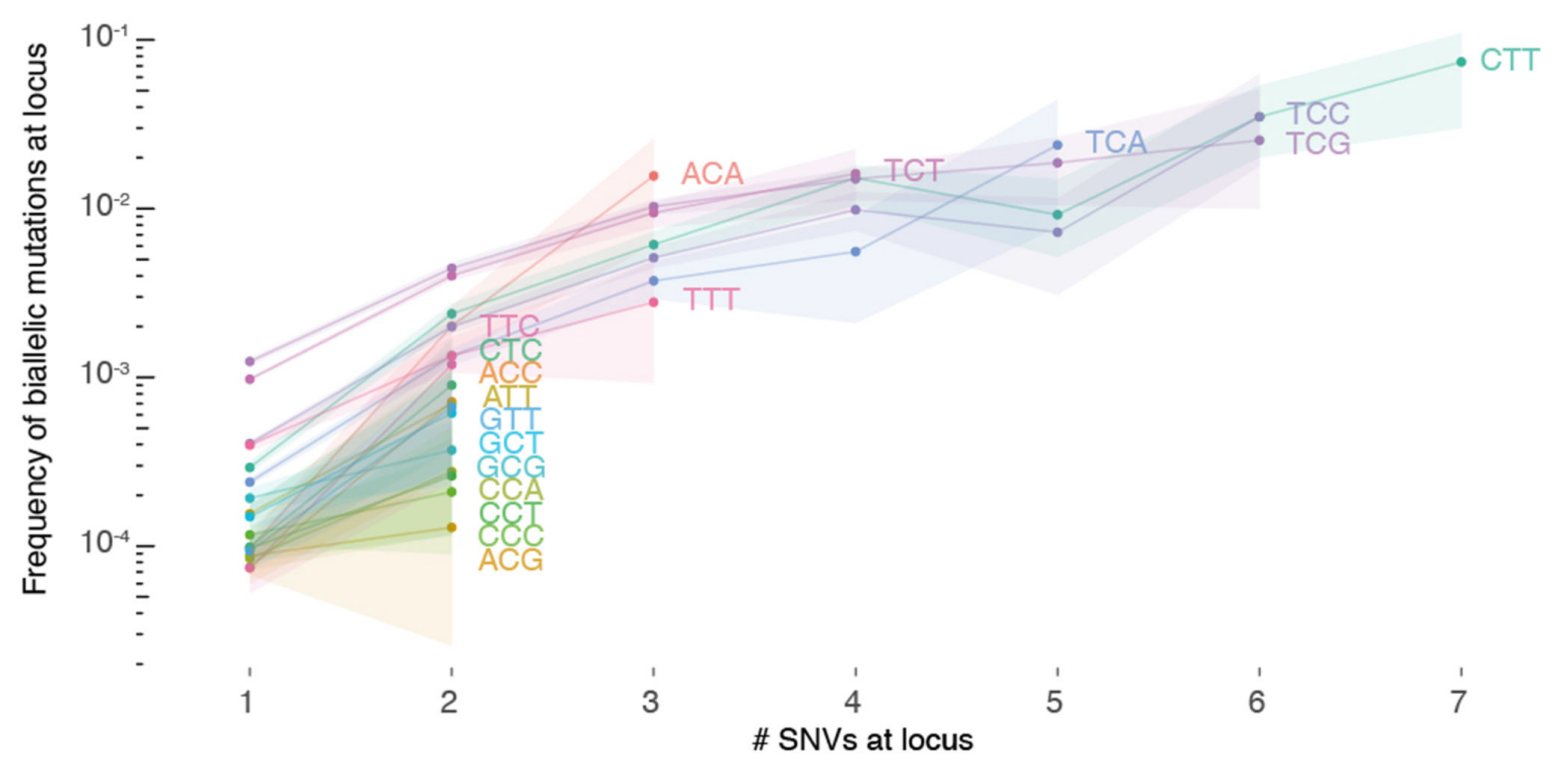
Loci with biallelic mutations have higher intrinsic mutability. The fraction of loci with biallelic mutations is plotted for loci with 1, 2, …, 7 monoallelic SNVs across PCAWG. Loci are further stratified per trinucleotide context and those with annotated driver mutations are excluded. Bootstrap resampling is performed to obtain 95% confidence intervals (shaded).

**Extended Data Fig. 8:**
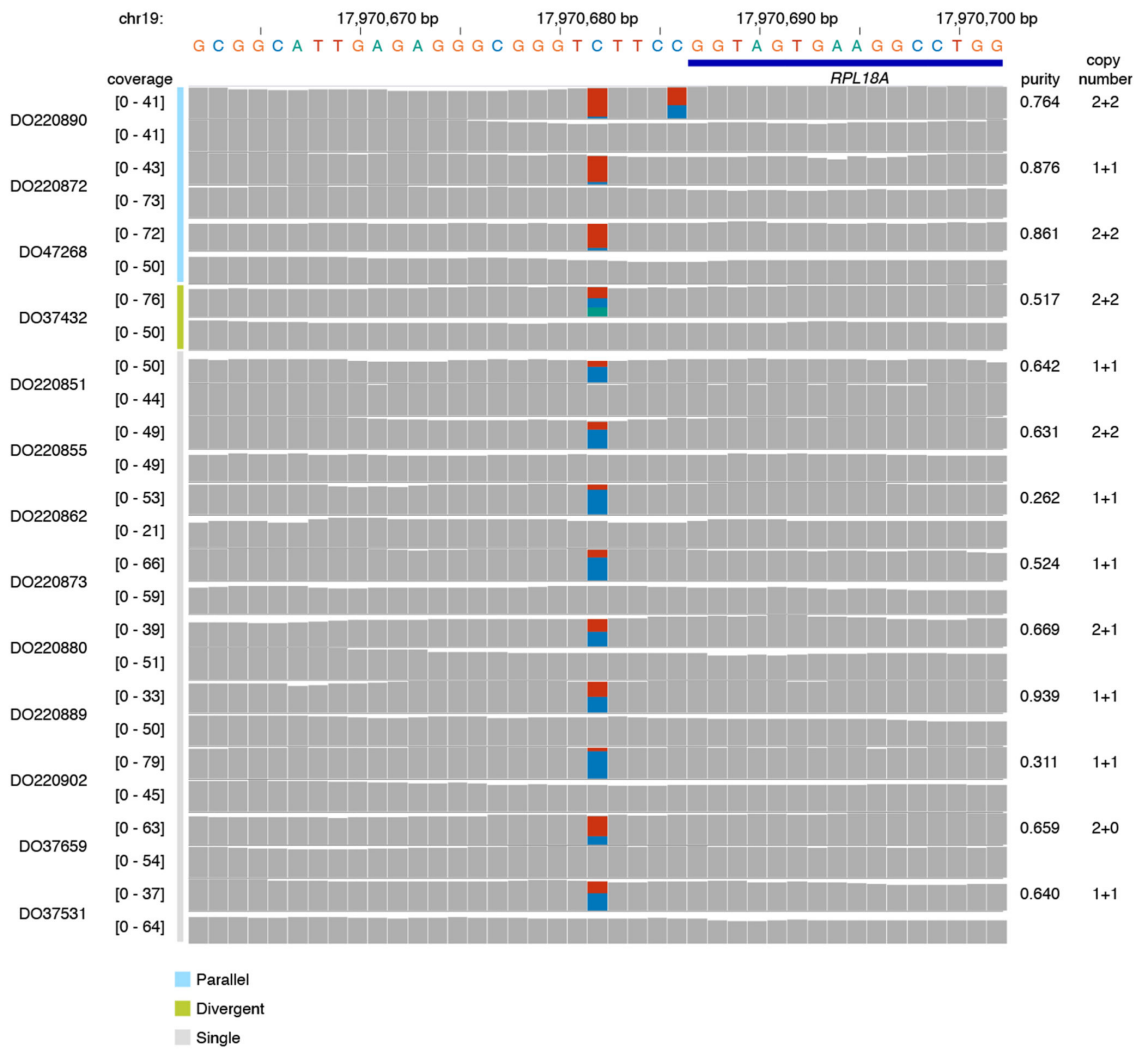
Recurrent mono- and biallelic mutation of the RPL18A promoter. Histograms of read coverage in 13 melanoma tumour-normal pairs showing mono- or biallelic mutation of the ETS-binding T**C**I 1CCG motif at the *RPL18A* promoter.

**Extended Data Fig. 9:**
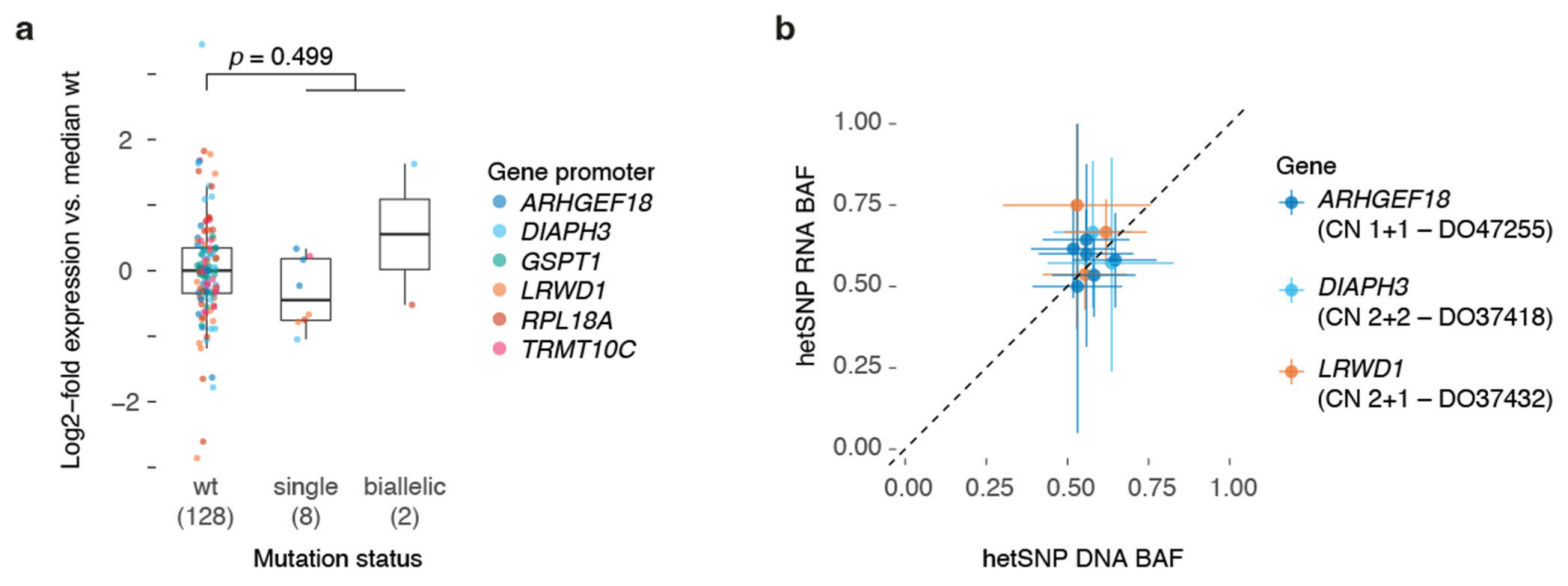
Effect of promoter mutation on gene expression for genes with biallelic hits. (**a**) Box and scatter plot showing the log2-fold change in expression (FPKM-UQ, Methods) compared to the median wild type for promoter mutated genes in **Figure 5a**. Each dot represents the relative expression in a single PCAWG melanoma with RNA-Seq data, stratified by the mutation status of that gene’s promoter. The total number of tumours for each category is indicated between parentheses. Centre line, median; box limits, upper and lower quartiles; whiskers, 1.5x interquartile range. A two-sided Student’s *í*-test was used to evaluate the difference between the log2-transformed expression values of wild type vs the pooled single and biallelic mutant cases. (**b**) Scatter plot of the DNA and RNA B- allele frequencies of expressed germline heterozygous SNPs in the genes/samples with a single mutant promoter allele in (a). The ICGC donor ID and local consensus copy number are indicated. Error bars and the centre represent, respectively, the posterior 95% highest density interval and maximum likelihood estimate of the DNA and RNA B-allele frequencies assuming a uniform *Beta(1,1*) prior and a binomial likelihood for the allele counts.

**Extended Data Fig. 10:**
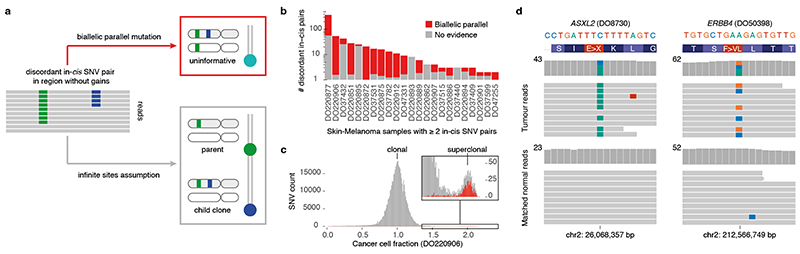
Biallelic mutations can confound common analysis. (**a**) Patterns of in-*cis* SNV pairs in a diploid region evidence linear phylogenies (parent-child) when the infinite sites assumption holds. (**b**) Bar plot showing the number of in-*cis* SNV pairs in PCAWG melanoma samples with at least two such pairs. Bar height reflects total numbers observed while the red portion indicates the fraction of all pairs with evidence for biallelic parallel mutation (beta-binomial *p*-value ≤ 0.05 or phasing to a heterozygous SNP). (**c**) Histogram of cancer cell fractions of SNVs in melanoma DO220906. The clonal cluster and a superclonal cluster containing mainly biallelic parallel mutations (red), are indicated. (**d**) IGV visualisation of two missed biallelic drivers in colorectal and oesophageal adenocarcinomas DO8730 and DO50398, respectively. Reads (horizontal bars) are downsampled for clarity and local basewise coverage is indicated left of the histograms.

## Supplementary Material

Inventory of Supporting Information

Supplementary Figures 1-2

Supplementary Tables 1-7

## Figures and Tables

**Figure 1:**
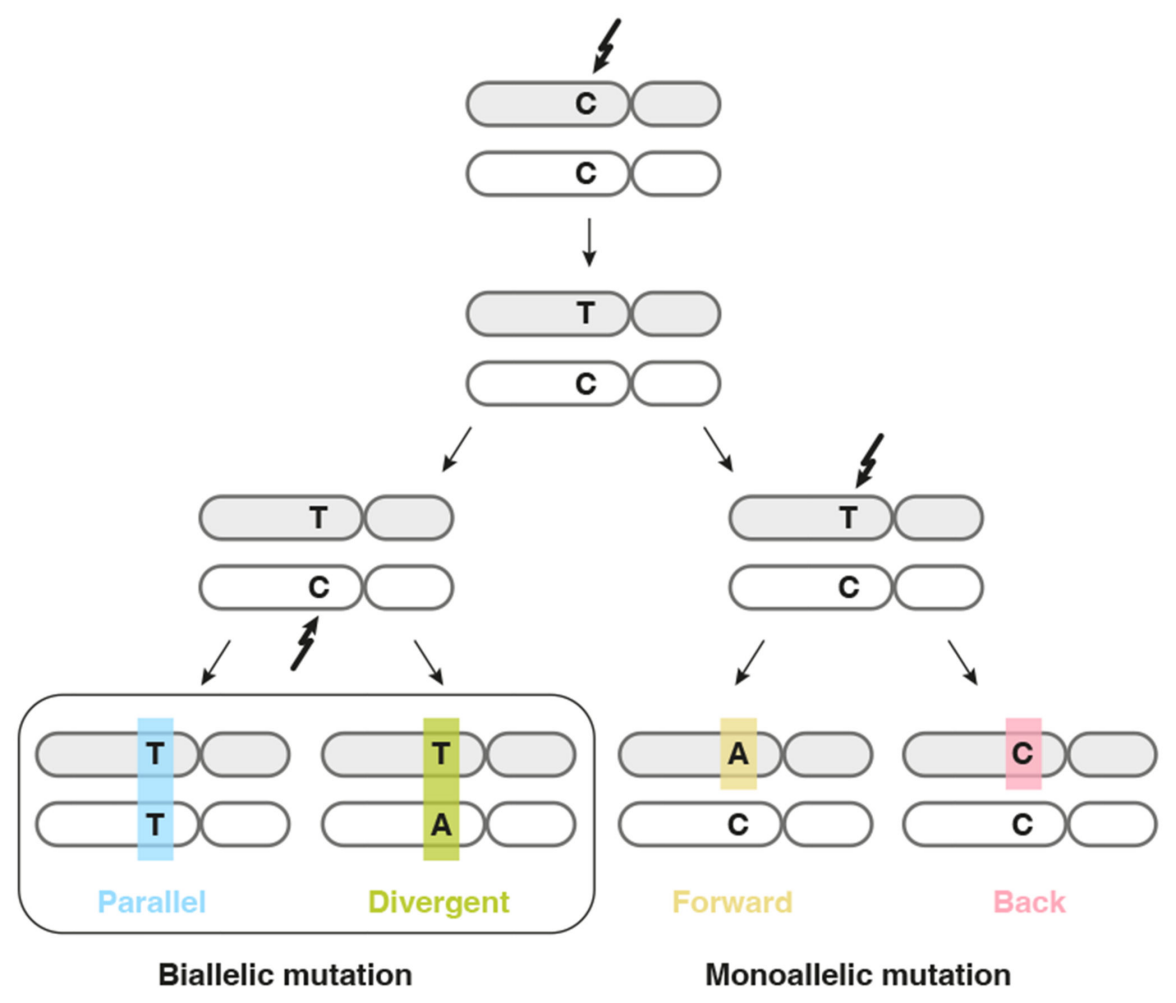
Possible violations of the infinite sites assumption in a single clonal lineage. Two subsequent mutations at a diploid locus can affect the same or alternate alleles. Depending on the base changes, there are four scenarios: biallelic parallel or divergent mutations affect separate alleles, whereas monoallelic forward and back mutation hit the same allele twice.

**Figure 2:**
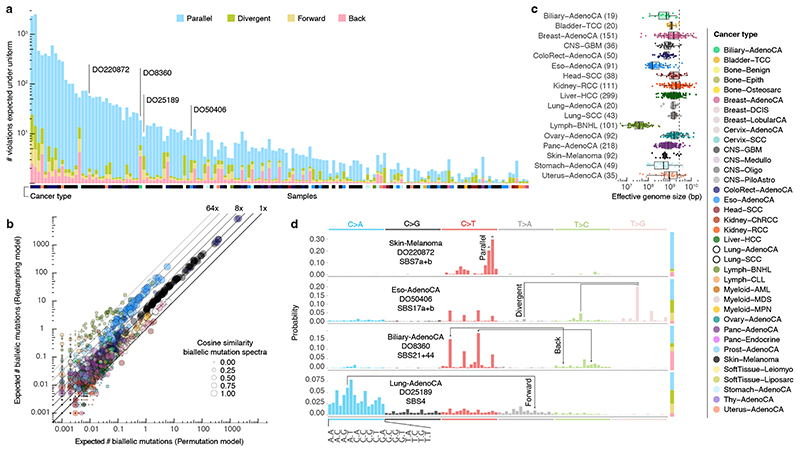
Simulated landscape of infinite sites violations in the PCAWG cohort. (**a**) Number and type of infinite sites violations in 147 PCAWG samples with ≥ 1 expected violation under a uniform mutation distribution. Bar height indicates the expected number of violations and coloured subdivisions represent the fractions contributed by each violation type. Tumour type of the samples is colour-coded below the bars. The four samples highlighted in (d) are indicated. (**b**) Comparison of the expected biallelic violations from the uniform permutation and neighbour resampling models. Every dot represents a tumour simulated 1,000x with each model. Colour and size reflect, respectively, tumour type and the cosine similarity of the predicted biallelic mutation spectra. (**C**) Box and scatterplot showing the effective genome size perceived by the mutational processes per cancer type, as estimated from the per-sample differences between simulation approaches. The dashed line indicates the callable genome size. The effective genome size is smallest in Lymph-BNHL (~37Mb), likely driven by recurrent focal hypermutation^13^. Centre line, median; box limits, upper and lower quartiles; whiskers, 1.5x interquartile range. Only tumours with ≥ 10 biallelic mutations across 1,000 simulations are included and their numbers are indicated between parentheses next to the tumour type. Only tumour types with ≥ 10 such tumours are shown. (**d**) Mutation spectra of four tumours with distinct violation contributions indicated in (a). The 16 distinct trinucleotide contexts are provided on the x-axis for C>A type substitutions and are the same for each coloured block. The proportion of parallel, divergent, back and forward mutation is indicated in the stacked bar on the right. Frequent combinations of mutations leading to specific infinite site violations are highlighted as well as the signatures generating them.

**Figure 3:**
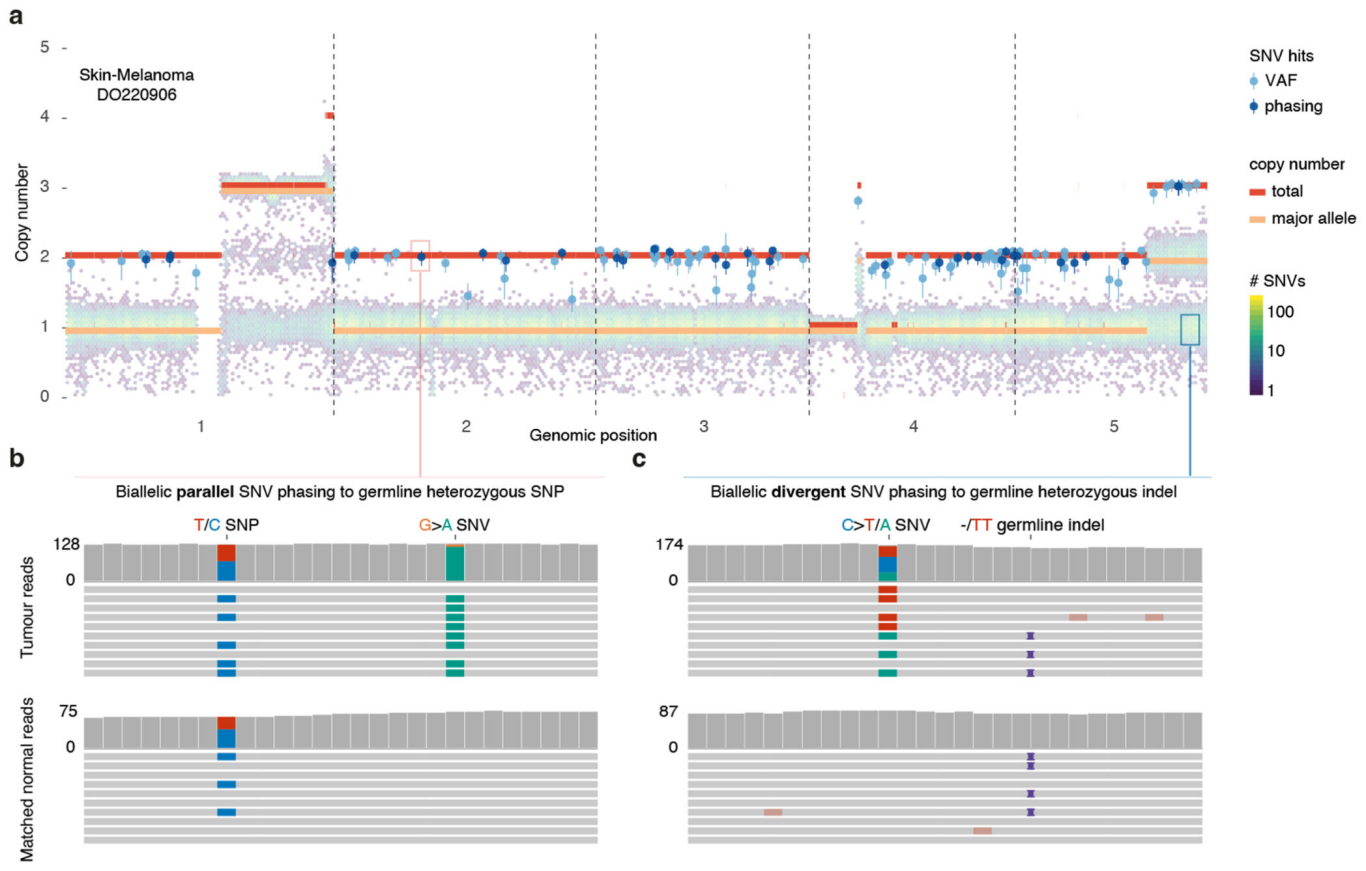
Detecting biallelic mutations in a case of melanoma. (**a**) Tumour allele-specific copy number and binned mutation copy number plotted for chromosomes 1–5 of melanoma DO220906. Somatic SNVs with a mutation copy number exceeding that of the major allele (and equal to the total copy number) are evident, suggesting biallelic parallel mutation events. Error bars and their centres represent, respectively, the posterior 95% highest density interval and maximum likelihood estimate obtained from a beta-binomial model of the observed reference and alternate allele read counts with a uniform *Beta*(1,1) prior (Methods). (**b,c**) IGV visualisation of DO220906 tumour (top) and matched normal (bottom) sequencing data at two loci, illustrating how read phasing information can confirm independent mutation of both parental alleles for (**b**) parallel and (**C**) divergent mutations. Reads (horizontal bars) are downsampled for clarity and local base-wise coverage is indicated left of the histograms. In total, we identify 373 parallel mutations (74 supported by phasing) and 8 divergent mutations in DO220906.

**Figure 4:**
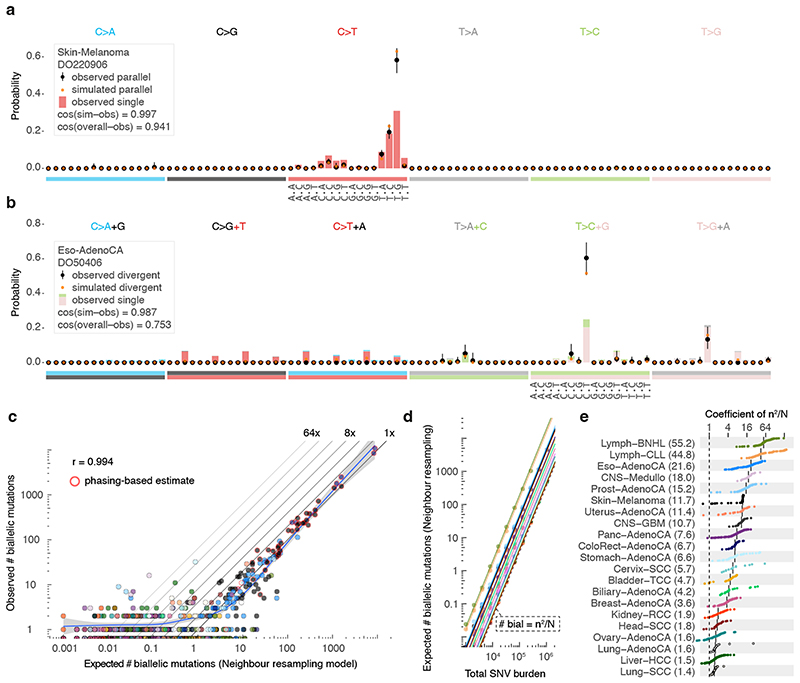
Comparison between observed and simulated biallelic mutations. (**a**) Bar chart highlighting the mutation spectrum of observed and predicted parallel mutations (circles) as well as the background SNVs for melanoma DO220906 (bars). Cosine similarities between the spectra are indicated. Error bars represent the 95% confidence intervals obtained from a Dirichlet-multinomial model of the observed biallelic parallel mutation type counts with a uniform Dirichlet prior. (**b**) Similar as (a) but showing divergent mutations for oesophageal adenocarcinoma DO50406. Bars are stacked to reflect the frequency of the colour-coded base changes indicated on top. (**c**) Scatterplot of the observed *vs*. neighbour resampling model-expected number of biallelic mutations (parallel + divergent) for all PCAWG tumours. For cases with ≥10,000 phaseable SNVs (red borders), the phasing-based number is provided. Colours reflect tumour type as in Figure 2. The Pearson correlation and a spline regression fit with 95% confidence interval (shaded grey) are shown. (**d**) Number of biallelic violations expected according to the neighbour resampling model for a range of mutation burdens and tumour types. The dashed line indicates the birthday problem estimate equal to the square of the mutation burden divided by the genome size (*m^2^/N*). Full coloured lines are the linear fits per tumour type. (**e**) Bar plot of the fitted coefficients of *m^2^/N* as derived in (**d**). For each tumour type, the ICGC donor ID indicates the representative tumour used.

**Figure 5:**
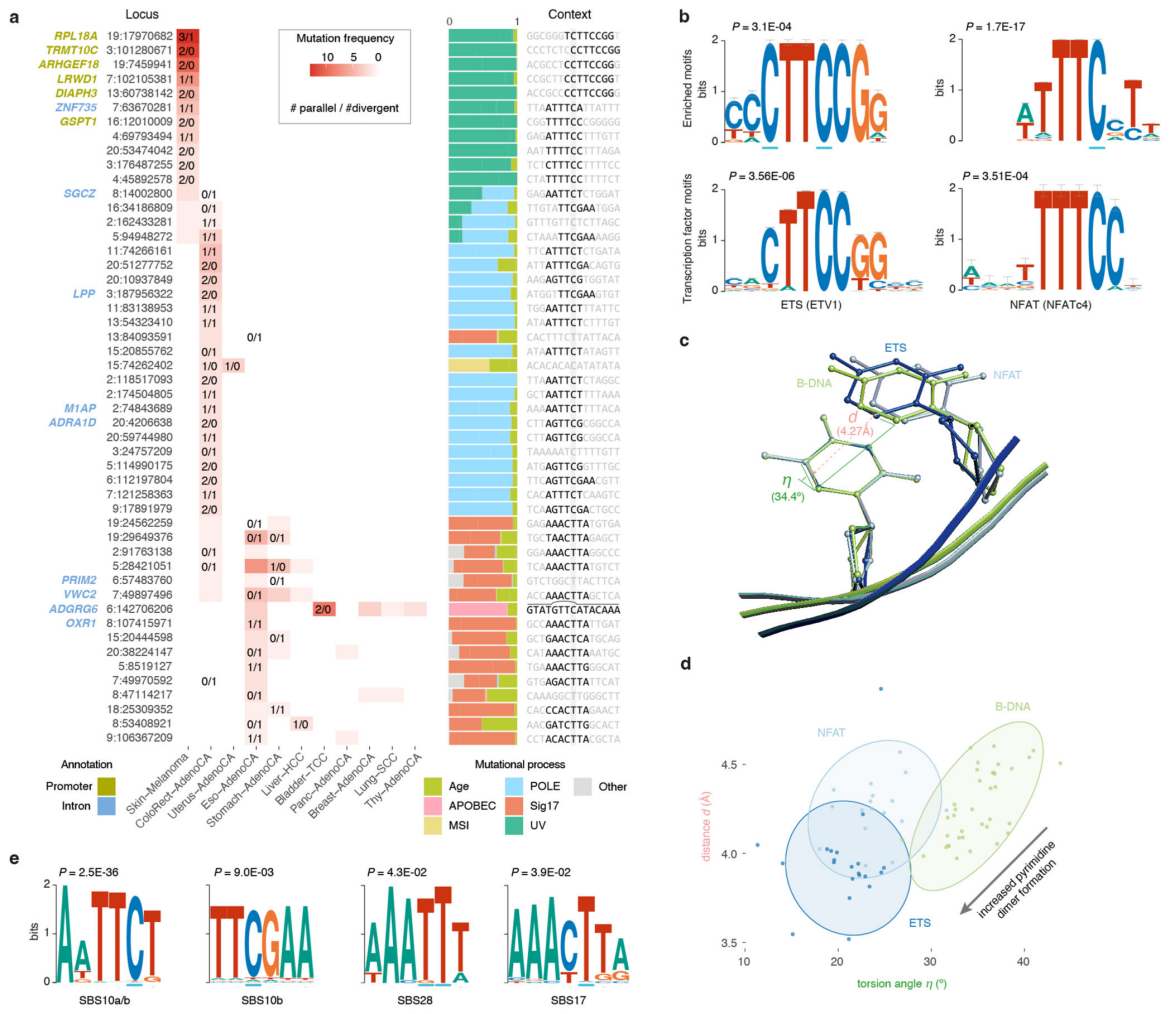
Biallelic mutations reveal hotspot motifs. (**a**) Heatmap of the fifty most frequently mutated loci in PCAWG with at least one biallelic mutation. The number of parallel/divergent mutations at each site is indicated, as are gene annotations, the underlying mutational processes, and the local sequence context with emerging motifs. For chr6:142,706,206, part of the stem and loop of a local sequence palindrome are indicated. MSI, microsatellite instability. (**b**) Sequence logos of motifs enriched at loci with biallelic mutations in melanoma (top) and corresponding transcription factor recognition sequences (bottom). Error bars indicate the confidence of a motif based on the number of sites used in its creation. Fisher’s exact test is used to assess motif enrichment (top) while *P*-values for motif comparison (bottom) are computed and corrected for multiple testing according to Gupta *et al*.^17^. (**c**) Superposition of TpC dinucleotides in crystal structures of ETS-bound (GABP), NFAT-bound (NFAT1c) and free B-form DNA (PDB IDs, 1AWC, 1OWR and 1BNA, respectively). The distance *d* between the midpoints of the two adjacent C5-C6 bonds as well as their torsion angle *η* is indicated. (**d**) Scatter plot showing the distance *d* and angle *η* indicated in (**c**) for TpC dinucleotides in structures of ETS-bound (dark blue), NFAT-bound (blue) or free B-form DNA (green) obtained from the RCSB protein data bank (Supplementary Table 7). Ellipses represent the normal-probability contours of each group. Lower values of *d* and *η* increase the yield of UV-based pyrimidine dimer formation, as indicated by the arrow. (**e**) Sequence logos of motifs enriched at loci with biallelic mutations in colorectal adenocarcinoma (SBS10, 28) and oesophageal/stomach adenocarcinoma (SBS17). Fisher’s exact test is used to assess motif enrichment.

## Data Availability

The Pan-Cancer Analysis of Whole-Genomes (PCAWG) dataset is available through the ICGC data portal at https://dcc.icgc.org/pcawg ^13^. Further information on accessing the data, including raw read files, can be found at https://docs.icgc.org/pcawg/data/. In accordance with the data access policies of the ICGC and TCGA projects, most molecular, clinical and specimen data are in an open tier that does not require access approval. To access information that could potentially identify participants, such as germline alleles and underlying sequencing data, researchers will need to apply to the TCGA Data Access Committee (DAC) via dbGaP (https://dbgap.ncbi.nlm.nih.gov/aa/wga.cgi?page=login) for access to the TCGA portion of the dataset, and to the ICGC Data Access Compliance Office (DACO; http://icgc.org/daco) for the ICGC portion. In addition, to access somatic SNVs derived from TCGA donors, researchers will also need to obtain dbGaP authorization. Structural data were obtained from the RCSB Protein Data Bank (https://www.rcsb.org/). The HOCOMOCO Human v11 Core set was used as the source of known transcription factor recognition sequences (https://hocomoco11.autosome.ru/). NCBI Curated Common Structural Variants are available via NCBI dbVar at https://www.ncbi.nlm.nih.gov/dbvar/studies/nstd186/. The germline resources of the 1,000 Genomes Project and gnomAD were respectively obtained from https://www.internationalgenome.org/ and https://gnomad.broadinstitute.org/.

## Code availability

The core computational pipelines used by the PCAWG Consortium for alignment, quality control and variant calling are available to the public at https://dockstore.org/search?search=pcawg under the GNU General Public License v3.0, which allows for reuse and distribution. All custom scripts for simulating, identifying and characterizing biallelic mutations from PCAWG data are available on GitHub at https://github.com/jdemeul/InfiniteSites. R v4.0.0 was used for the final analyses. Variant recalling on 195 PCAWG samples was done using GATK v4.0.8.1, which is available from https://gatk.broadinstitute.org/. STREME and TomTom v5.3.2 (MEME suite, https://meme-suite.org/) were used for motif enrichment and analysis. PCAWG consensus SNV, MNV, indel and structural variant calling was described in The ICGC/TCGA Pan-Cancer Analysis of Whole Genomes Consortium^13^. PCAWG consensus copy number calling was described by Dentro *et al*.^4^. PCAWG gene expression analysis was reported in Calabrese *et al*.^27^ and was based on TopHat2 v2.0.12 and STAR v2.4.0i alignments. PyMOL v2.4.0 was used for structural analyses.
