# Supplementary material for: Biallelic mutations in cancer genomes reveal local mutational determinants
Source: Nat Genet. Author manuscript; Available in PMC 2022 Feb 15. (PMC8837546; doi:10.1038/s41588-021-01005-8)
Supplement: Inventory of Supporting Information [file EMS140579-supplement-Inventory_of_Supporting_Information.docx]

Inventory of Supporting Information

**Manuscript #:** NG-LE57231R1.

**Corresponding author name(s):** Jonas Demeulemeester, Peter Van Loo.

| Please complete each of the Inventory Tables below to outline your Extended Data and Supplementary Information items.  There are four sections:   - *Extended Data* - *Supplementary Information: Flat Files* - *Supplementary Information: Additional Files* - *Source Data*   Each section includes specific instructions. Please complete these tables as fully as possible. We ask that you avoid using spaces in your file names, and instead use underscores, i.e.: Smith_ED_Fig1.jpg not Smith ED Fig1.jpg  Please note that titles and descriptive captions will only be lightly edited, so please ensure that you are satisfied with these prior to submission.  If you have any questions about any of the information contained in this inventory, please contact the journal. |
| --- |
|  |

1. **Extended Data**

**Complete the Inventory below for all Extended Data figures.**

- Keep Figure Titles to one sentence only
- Upload your files as ‘Figure Files’ in our Manuscript Tracking system
- File names should include the Figure Number. i.e.: *Smith_ED_Fig1.jpg*
- Please be sure to include the file extension in the Filename. Note that Extended Data files must be submitted as .jpg, .tif or .eps files *only*, and should be approximately 10MB
- All Extended Data figure legends must be provided in the Inventory below and should not exceed 300 words each *(if possible)*
- Please include Extended Data *ONLY* in this table

| Figure # | Figure title  One sentence only | Filename  This should be the name the file is saved as when it is uploaded to our system. Please include the file extension. i.e.: *Smith_ED_Fig1.jpg* | Figure Legend  If you are citing a reference for the first time in these legends, please include all new references in the main text Methods References section, and carry on the numbering from the main References section of the paper. If your paper does not have a Methods section, include all new references at the end of the main Reference list. |
| --- | --- | --- | --- |
| Extended Data Fig. 1 | Simulation approaches for infinite sites violations. | ExtendedDataFigure1.jpg | Schematic overview of the uniform permutation (left) and neighbour resampling (right) approaches to assess the number and type of infinite sites violations expected in a tumour. Numbers in the uniform permutation panel highlight the sequential nature of the sampling, which keeps track of mutated positions to consider them accordingly for biallelic, forward, and back mutation. Note that the neighbour resampling model excludes all PCAWG annotated driver mutations and allows simulation of biallelic events only. |
| Extended Data Fig. 2 | Biallelic indels are expected in a subset of microsatellite unstable tumours. | ExtendedDataFigure2.jpg | Bar plots of the observed indel burden and signature (left) and the expected biallelic indels according to the neighbour resampling model (right). Bar height indicates total numbers and coloured subdivisions represent fractions contributed by each indel signature (left) or biallelic indel type (right). Only PCAWG tumours with ≥1 expected biallelic indel are shown. Four microsatellite unstable tumours are predicted to boast several hundreds to over one thousand, mostly parallel, biallelic indels. These mainly originate from indel signatures 1 and 2, likely reflecting slippage during DNA replication and subsequent 1bp T (or A) insertion and deletion in thymine (adenosine) mononucleotide repeats, respectively. |
| Extended Data Fig. 3 | Detection of biallelic parallel mutations by allele frequency. | ExtendedDataFigure3.jpg | (**a**) Flow chart showing the filtering steps, phasing-based estimates of precision and lower bound recall, as well as the input and output data for our pipeline to detect biallelic parallel mutations in PCAWG based on variant allele frequencies (VAF). Three filtering steps highlighted in bold are further illustrated in panels (b-d). (**b**) Rainfall plot of all biallelic parallel hits obtained after omitting the germline SV filter. Streaks of coloured dots indicate a clustering of hits in regions with common germline structural variants. While demonstrating the ability of the pipeline to detect VAF outliers, these hits are poorly supported by phasing data and likely represent single somatic SNVs in the context of a heterozygous germline deletion. (**c**) Example of reference bias in the PCAWG consensus SNV read counts. Reads carrying the somatic variant contain alternate germline alleles at three proximal positions, resulting in an underreporting of the number of wild type reads and an overestimation of the VAF. (**d**) Diagnostic QQ-plots of the unadjusted one-sided beta-binomial read count test *P*-values for two samples (**Methods**). DO41578 *P*-values are overinflated (slope > 1), hinting at consensus purity/ploidy errors, and the sample is excluded as a result. |
| Extended Data Fig. 4 | Biallelic parallel mutation during metastatic prostate cancer evolution. | ExtendedDataFigure4.jpg | Heatmap showing allele frequencies of variants found to be biallelic in at least one sample of eight prostate cancers with sequencing of matched primary and metastases (A10–A34, different sites indicated as in Gundem et al.^12^). Early clonal biallelic mutations are detected in all samples of a patient (*e.g.,* A10 chr19:29,339,579), while late clonal and subclonal ones show no evidence of being biallelic in some samples (beta-binomial p-value > 0.05 and no discordant phasing to a heterozygous germline SNP) or are detected in only a subset of samples (*e.g.,* A22 chr14:61,497,015 and A10 chr2:117,463,664, respectively). |
| Extended Data Fig. 5 | Landscape of biallelic mutations across PCAWG. | ExtendedDataFigure5.jpg | Number of observed parallel (red) and divergent (blue) mutations plotted in context of the total SNV burden for 84 PCAWG samples with ≥ 1 phasing-confirmed VAF hit. The range of parallel mutations expected purely from SNV-SNP phasing is also indicated (95% confidence interval, red vertical bars) as this approach is less sensitive to purity and copy number state than the VAF-based analysis. Samples for which the number of divergent mutations is not shown were not considered for Mutect2 recalling. |
| Extended Data Fig. 6 | Comparison between observed and simulated biallelic mutations. | ExtendedDataFigure6.jpg | (**a,b**) Scatterplots of the observed *vs.* expected number of biallelic mutations (parallel + divergent) for all PCAWG tumours using the uniform permutation (**a**) and neighbour resampling models (**b**). The Pearson correlation and a spline regression fit with 95% confidence interval (shaded grey) are shown. |
| Extended Data Fig. 7 | Loci with biallelic mutations have higher intrinsic mutability. | ExtendedDataFigure7.jpg | The fraction of loci with biallelic mutations is plotted for loci with 1, 2, …, 7 monoallelic SNVs across PCAWG. Loci are further stratified per trinucleotide context and those with annotated driver mutations are excluded. Bootstrap resampling is performed to obtain 95% confidence intervals (shaded). |
| Extended Data Fig. 8 | Recurrent mono- and biallelic mutation of the RPL18A promoter. | ExtendedDataFigure8.jpg | Histograms of read coverage in 13 melanoma tumour-normal pairs showing mono- or biallelic mutation of the ETS-binding T**C**TTCCG motif at the *RPL18A* promoter. |
| Extended Data Fig. 9 | Effect of promoter mutation on gene expression for genes with biallelic hits. | ExtendedDataFigure9.jpg | (**a**) Box and scatter plot showing the log2-fold change in expression (FPKM-UQ, **Methods**) compared to the median wild type for promoter mutated genes in **Figure 5a**. Each dot represents the relative expression in a single PCAWG melanoma with RNA-Seq data, stratified by the mutation status of that gene’s promoter. The total number of tumours for each category is indicated between parentheses. Centre line, median; box limits, upper and lower quartiles; whiskers, 1.5x interquartile range. A two-sided Student’s *t*-test was used to evaluate the difference between the log2-transformed expression values of wild type vs the pooled single and biallelic mutant cases. (**b**) Scatter plot of the DNA and RNA B-allele frequencies of expressed germline heterozygous SNPs in the genes/samples with a single mutant promoter allele in (a). The ICGC donor ID and local consensus copy number are indicated. Error bars and the centre represent, respectively, the posterior 95% highest density interval and maximum likelihood estimate of the DNA and RNA B-allele frequencies assuming a uniform $Beta\left( 1,1 \right)$ prior and a binomial likelihood for the allele counts. |
| Extended Data Fig. 10 | Biallelic mutations can confound common analysis. | ExtendedDataFigure10.jpg | (**a**) Patterns of in-*cis* SNV pairs in a diploid region evidence linear phylogenies (parent-child) when the infinite sites assumption holds. (**b**) Bar plot showing the number of in-*cis* SNV pairs in PCAWG melanoma samples with at least two such pairs. Bar height reflects total numbers observed while the red portion indicates the fraction of all pairs with evidence for biallelic parallel mutation (beta-binomial *p*-value ≤ 0.05 or phasing to a heterozygous SNP). (**c**) Histogram of cancer cell fractions of SNVs in melanoma DO220906. The clonal cluster and a superclonal cluster containing mainly biallelic parallel mutations (red), are indicated. (**d**) IGV visualisation of two missed biallelic drivers in colorectal and oesophageal adenocarcinomas DO8730 and DO50398, respectively. Reads (horizontal bars) are downsampled for clarity and local base-wise coverage is indicated left of the histograms. |

***Delete rows as needed to accommodate the number of figures (10 is the maximum allowed).***

1. **Supplementary Information:**
2. **Flat Files**

**Complete the Inventory below for all additional textual information and any additional Supplementary Figures, which should be supplied in one combined PDF file.**

- **Row 1:** A combined, flat PDF containing any Supplementary Text, Discussion, Notes, Additional Supplementary Figures, Supplementary Protocols, simple tables, and all associated legends. Only one such file is permitted.
- **Row 2:** Nature Research’s Reporting Summary; if previously requested by the editor, please provide an updated Summary, fully completed, without any mark-ups or comments. **(Reporting Summaries are not required for all manuscripts.)**

| Item | Present? | Filename  This should be the name the file is saved as when it is uploaded to our system, and should include the file extension. The extension must be .pdf | A brief, numerical description of file contents.  i.e.: *Supplementary Figures 1-4, Supplementary Discussion, and Supplementary Tables 1-4.* |
| --- | --- | --- | --- |
| Supplementary Information | Yes | 20211024_Demeulemeester_InfSites_SuppInfo.pdf | Supplementary Figures 1-2 |
| Reporting Summary | Yes | nr-reporting-summary-final.pdf |  |
| Peer Review Information | Yes | *OFFICE USE ONLY* |  |

1. **Additional Supplementary Files**

**Complete the Inventory below for all additional Supplementary Files that cannot be submitted as part of the Combined PDF.**

- Do not list Supplementary Figures in this table (see section 2A)
- Where possible, include the title and description within the file itself
- Spreadsheet-based tables & data should be combined into a workbook with multiple tabs, not submitted as individual files.
- Compressed files are acceptable where necessary. ZIP files are preferred.
- Please note that the *ONLY* allowable types of additional Supplementary Files are:

| - Supplementary Tables | - Supplementary Audio | - Supplementary Videos | - Supplementary Software |
| --- | --- | --- | --- |
| - Supplementary Data, for example: raw NMR Data, Cryo-EM Data, Computational Data, Crystallographic Data, etc. | | | |

| Type | Number  If there are multiple files of the same type this should be the numerical indicator. i.e. “1” for Video 1, “2” for Video 2, etc. | Filename  This should be the name the file is saved as when it is uploaded to our system, and should include the file extension. i.e.: *Smith_ Supplementary_Video_1.mov* | Legend or Descriptive Caption  Describe the contents of the file |
| --- | --- | --- | --- |
| Supplementary Table | 1 | SupplementaryTables.xlsx | Supplementary Tables 1-7 |

***Add rows as needed to accommodate the number of files.***

1. **Source Data**

**Complete the Inventory below for all Source Data files.**

- Acceptable types of Source Data for Main Figures and Extended Data Figures are:
  - Statistical Source Data
    - Plain Text (ASCII, TXT) or Excel formats only
    - One file for each relevant Figure, containing all source data
  - Full-length, unprocessed Gels or Blots
    - JPG, TIF, or PDF formats only
    - One file for each relevant Figure, containing all supporting blots and/or gels
- ‘Source Data’ is only allowed for Main Figures and Extended Data Figures.
  - Include Unprocessed Gels or Blots for Supplementary Figures as additional Supplementary Figures.
  - Include Statistical Source Data for Supplementary Figures as ‘Supplementary Data’ files and list them in section 2B.
  - Please see [this example of Source Data](https://www.nature.com/articles/s41591-019-0505-4) in a publication.

| Parent Figure or Table | Filename  This should be the name the file is saved as when it is uploaded to our system, and should include the file extension. i.e.: *Smith_SourceData_Fig1.xls,* or *Smith_ Unmodified_Gels_Fig1.pdf* | Data description  i.e.: Unprocessed Western Blots and/or gels, Statistical Source Data, etc. |
| --- | --- | --- |
