# Supplementary material for: Biallelic mutations in cancer genomes reveal local mutational determinants
Source: Nat Genet. 2022 Feb 10;54(2):128–33. doi: 10.1038/s41588-021-01005-8 (PMC8837546; doi:10.1038/s41588-021-01005-8)
Supplement: Supplementary file 1 [file 41588_2021_1005_MOESM1_ESM.pdf]

nature portfolio

https://doi.org/10.1038/s41588-021-01005-8

Supplementary information

Biallelic mutations in cancer genomes reveal local mutational determinants

In the format provided by the
authors and unedited

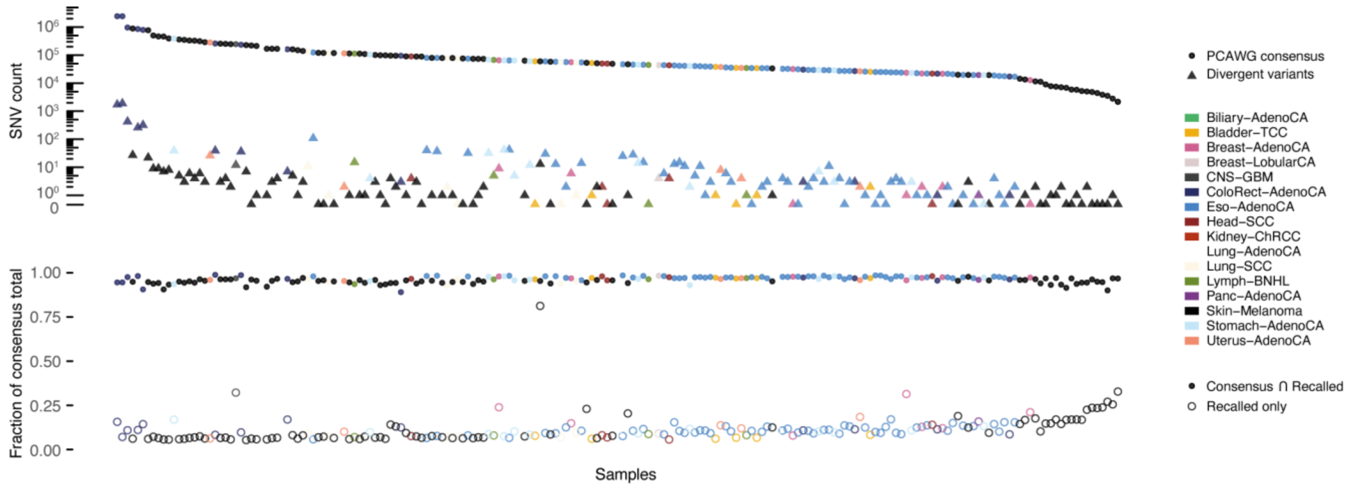

Supplementary Figure 1

Variant recalling results on 195 PCAWG tumours. Dot plot showing the total number of PCAWG consensus SNV calls and the number of divergent mutations identified after recalling with Mutect2 (top), the fraction of PCAWG consensus calls recovered during recalling and the fraction of new calls (bottom).

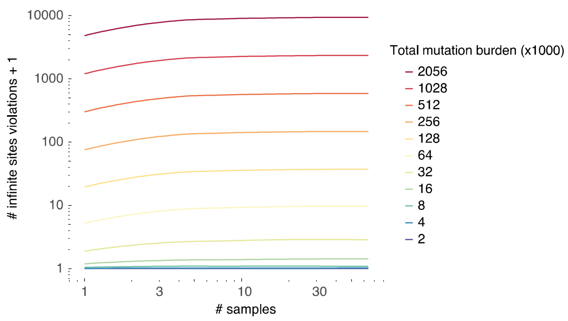

Supplementary Figure 2

Infinite sites violations in a multi-sample setting. Simulation results showing how the number of infinite sites violations increases when multiple samples are considered with the indicated total mutational load. Note that in an actual multi-sample setting, the total mutation burden generally increases with the number of samples.
