# Supplementary material for: Biallelic mutations in cancer genomes reveal local mutational determinants
Source: Nat Genet. 2022 Feb 10;54(2):128–33. doi: 10.1038/s41588-021-01005-8 (PMC8837546; doi:10.1038/s41588-021-01005-8)
Supplement: Supplementary file 2 [file 41588_2021_1005_MOESM2_ESM.pdf]

nature research

Corresponding author(s): Peter Van Loo
Jonas Demeulemeester

Last updated by author(s): Oct 24, 2021

Reporting Summary

Nature Research wishes to improve the reproducibility of the work that we publish. This form provides structure for consistency and transparency in reporting. For further information on Nature Research policies, see our Editorial Policies and the Editorial Policy Checklist.

Statistics

For all statistical analyses, confirm that the following items are present in the figure legend, table legend, main text, or Methods section.

n/a	Confirmed	
☐	☒	The exact sample size (*n*) for each experimental group/condition, given as a discrete number and unit of measurement
☐	☒	A statement on whether measurements were taken from distinct samples or whether the same sample was measured repeatedly
☐	☒	The statistical test(s) used AND whether they are one- or two-sided<br>*Only common tests should be described solely by name; describe more complex techniques in the Methods section.*
☒	☐	A description of all covariates tested
☐	☒	A description of any assumptions or corrections, such as tests of normality and adjustment for multiple comparisons
☐	☒	A full description of the statistical parameters including central tendency (e.g. means) or other basic estimates (e.g. regression coefficient) AND variation (e.g. standard deviation) or associated estimates of uncertainty (e.g. confidence intervals)
☐	☒	For null hypothesis testing, the test statistic (e.g. F, t, r) with confidence intervals, effect sizes, degrees of freedom and P value noted<br>*Give P values as exact values whenever suitable.*
☐	☒	For Bayesian analysis, information on the choice of priors and Markov chain Monte Carlo settings
☒	☐	For hierarchical and complex designs, identification of the appropriate level for tests and full reporting of outcomes
☐	☒	Estimates of effect sizes (e.g. Cohen's *d*, Pearson's *r*), indicating how they were calculated

Our web collection on statistics for biologists contains articles on many of the points above.

Software and code

Policy information about availability of computer code

Data collection	No software was used for data collection.
Data analysis	The core computational pipelines used by the PCAWG Consortium for alignment, quality control and variant calling are available to the public at https://dockstore.org/search?search=pcawg under the GNU General Public License v3.0, which allows for reuse and distribution. All custom scripts for simulating, identifying and characterizing biallelic mutations from PCAWG data are available on GitHub at https://github.com/jdemeul/InfiniteSites.<br>R v4.0.0 was used for the final analyses. Variant recalling on 195 PCAWG samples was done using GATK v4.0.8.1, which is available from https://gatk.broadinstitute.org/. STREME and TomTom v5.3.2 (MEME suite, https://meme-suite.org/) were used for motif enrichment and analysis. PCAWG consensus SNV, MNV, indel and structural variant calling was described in The ICGC/TCGA Pan-Cancer Analysis of Whole Genomes Consortium, Nature, 2020. PCAWG consensus copy number calling was described in Dentro et al., Cell, 2021. PCAWG gene expression analysis was reported in Calabrese et al., Nature, 2020 and was based on TopHat2 v2.0.12 and STAR v2.4.0i alignments. PyMOL v2.4.0 was used for structural analyses.

For manuscripts utilizing custom algorithms or software that are central to the research but not yet described in published literature, software must be made available to editors and reviewers. We strongly encourage code deposition in a community repository (e.g. GitHub). See the Nature Research guidelines for submitting code & software for further information.

Data

Policy information about availability of data

All manuscripts must include a data availability statement. This statement should provide the following information, where applicable:

- Accession codes, unique identifiers, or web links for publicly available datasets
- A list of figures that have associated raw data
- A description of any restrictions on data availability

The Pan-Cancer Analysis of Whole-Genomes (PCAWG) dataset is available through the ICGC data portal at https://dcc.icgc.org/pcawg. Further information on accessing the data, including raw read files, can be found at https://docs.icgc.org/pcawg/data/. In accordance with the data access policies of the ICGC and TCGA projects, most molecular, clinical and specimen data are in an open tier that does not require access approval. To access information that could potentially identify participants, such as germline alleles and underlying sequencing data, researchers will need to apply to the TCGA Data Access Committee (DAC) via dbGaP (https://dbgap.ncbi.nlm.nih.gov/aa/wga.cgi?page=login) for access to the TCGA portion of the dataset, and to the ICGC Data Access Compliance Office (DACO; http://icgc.org/daco) for the ICGC portion. In addition, to access somatic SNVs derived from TCGA donors, researchers will also need to obtain dbGaP authorization. Structural data were obtained from the RCSB Protein Data Bank (https://www.rcsb.org/). The HOCOMOCO Human v11 Core set was used as the source of known transcription factor recognition sequences (https://hocomoco11.autosome.ru/). NCBI Curated Common Structural Variants are available via NCBI dbVar at https://www.ncbi.nlm.nih.gov/dbvar/studies/nstd186/. The germline resources of the 1,000 Genomes Project and gnomAD were respectively obtained from https://www.internationalgenome.org/ and https://gnomad.broadinstitute.org/.

Field-specific reporting

Please select the one below that is the best fit for your research. If you are not sure, read the appropriate sections before making your selection.

☒ Life sciences ☐ Behavioural & social sciences ☐ Ecological, evolutionary & environmental sciences

For a reference copy of the document with all sections, see nature.com/documents/nr-reporting-summary-flat.pdf

Life sciences study design

All studies must disclose on these points even when the disclosure is negative.

Sample size	The PCAWG consortium compiled an inventory of matched tumour/normal whole cancer genomes in the ICGC Data Coordinating Centre. Most samples came from treatment-naïve, primary cancers, but there were a small number of donors with multiple samples of primary, metastatic and/or recurrent tumours. The PCAWG inclusion criteria were: (i) matched tumour and normal specimen pair; (ii) a minimal set of clinical fields; and (iii) characterisation of tumour and normal whole genomes using Illumina HiSeq paired-end sequencing reads.<br>In total, the consortium collected genome data from 2,834 donors, representing all ICGC and TCGA donors that met these criteria at the time of the final data freeze in autumn 2014. No statistical methods were used to predetermine sample size.
Data exclusions	After quality assurance by the PCAWG consortium, data from 176 of its 2,834 donors were excluded as unusable. Reasons for data exclusions included inadequate coverage, extreme bias in coverage across the genome, evidence for contamination in samples and excessive sequencing errors (The ICGC/TCGA Pan-Cancer Analysis of Whole Genomes Consortium, Nature, 2020). These exclusion criteria were pre-determined.<br>In our neighbour resampling simulations, we additionally excluded samples that had been greylisted by the PCAWG consortium and used only the PCAWG designated representative sample for each patient with multi-region sequencing. In addition, we excluded 21 prostate cancer cases from the PRAD-CA cohort which were suspect of contamination, harbouring excess low VAF single nucleotide variant calls in repetitive regions of the genome as described in the corresponding Methods section.
Replication	In order to evaluate the performance of our biallelic parallel mutation calling pipeline, we have used phasing information from the paired-end reads. Precision and recall are assessed by taking all evaluated phaseable SNVs (i.e., SNP-SNV pairs having ≥ 2 reads each for the SNP Ref and Alt alleles and ≥ 4 reads reporting the SNV). Precision is calculated as the fraction of VAF-inferred biallelic parallel mutations which are confirmed by phasing. Recall is the fraction of phasing hits picked up through their allele frequencies. Overall performance is reported as the median precision and recall for samples with ≥ 10,000 phaseable SNVs and is estimated at 82% and ≥47.5%.
Randomization	No randomisation was performed - this was a descriptive study, not an experimental study.
Blinding	No blinding was undertaken - this was a descriptive study, not an experimental study.

Reporting for specific materials, systems and methods

We require information from authors about some types of materials, experimental systems and methods used in many studies. Here, indicate whether each material, system or method listed is relevant to your study. If you are not sure if a list item applies to your research, read the appropriate section before selecting a response.

Materials & experimental systems

n/a	Involved in the study
☒	☐ Antibodies
☒	☐ Eukaryotic cell lines
☒	☐ Palaeontology and archaeology
☒	☐ Animals and other organisms
☒	☐ Human research participants
☒	☐ Clinical data
☒	☐ Dual use research of concern

Methods

n/a	Involved in the study
☒	☐ ChIP-seq
☒	☐ Flow cytometry
☒	☐ MRI-based neuroimaging
