# Supplementary material for: Biallelic mutations in cancer genomes reveal local mutational determinants
Source: Nat Genet. 2022 Feb 10;54(2):128–33. doi: 10.1038/s41588-021-01005-8 (PMC8837546; doi:10.1038/s41588-021-01005-8)
Supplement: Supplementary file 3 [file 41588_2021_1005_MOESM3_ESM.pdf]

natureresearch

Peer Review Information

Journal: Nature Genetics
Manuscript Title: Biallelic mutations in cancer genomes reveal local mutational determinants
Corresponding author name(s): Dr Peter Van Loo

Reviewer Comments & Decisions:

Decision Letter, initial version:

12th May 2021

Dear Dr Van Loo,

Your Letter, "Biallelic mutations in cancer genomes reveal local mutational determinants" has now been seen by 3 referees. You will see from their comments below that while they find your work of interest, some important points are raised. We are interested in the possibility of publishing your study in Nature Genetics, but would like to consider your response to these concerns in the form of a revised manuscript before we make a final decision on publication.

To guide the scope of the revisions, the editors discuss the referee reports in detail within the team, including with the chief editor, with a view to identifying key priorities that should be addressed in revision and sometimes overruling referee requests that are deemed beyond the scope of the current study. From our reading of the reports, we think that all the reviewer comments are reasonable and constructive and should be addressed in full. However, we'd like you to pay particular attention to the major comment #2 raised by Reviewer #2 about the utility of identifying biallelic mutations and what it means in the context of common analysis. Moreover, we would urge you to make your code available to the reviewers for the next stage of review, should we reach that point.

Please do not hesitate to get in touch if you would like to discuss these issues further.

We therefore invite you to revise your manuscript taking into account all reviewer and editor comments. Please highlight all changes in the manuscript text file. At this stage we will need you to upload a copy of the manuscript in MS Word .docx or similar editable format.

We are committed to providing a fair and constructive peer-review process. Do not hesitate to contact us if there are specific requests from the reviewers that you believe are technically impossible or

unlikely to yield a meaningful outcome.

When revising your manuscript:

*1) Include a “Response to referees” document detailing, point-by-point, how you addressed each referee comment. If no action was taken to address a point, you must provide a compelling argument. This response will be sent back to the referees along with the revised manuscript.

*2) If you have not done so already please begin to revise your manuscript so that it conforms to our Letter format instructions, available
here.
Refer also to any guidelines provided in this letter.

*3) Include a revised version of any required Reporting Summary:
https://www.nature.com/documents/nr-reporting-summary.pdf
It will be available to referees (and, potentially, statisticians) to aid in their evaluation if the manuscript goes back for peer review.
A revised checklist is essential for re-review of the paper.

Please be aware of our guidelines on digital image standards.

Please use the link below to submit your revised manuscript and related files:

[REDACTED]

Note: This URL links to your confidential home page and associated information about manuscripts you may have submitted, or that you are reviewing for us. If you wish to forward this email to co-authors, please delete the link to your homepage.

We hope to receive your revised manuscript within four to eight weeks. If you cannot send it within this time, please let us know.

Please do not hesitate to contact me if you have any questions or would like to discuss these revisions further.

Nature Genetics is committed to improving transparency in authorship. As part of our efforts in this direction, we are now requesting that all authors identified as ‘corresponding author’ on published papers create and link their Open Researcher and Contributor Identifier (ORCID) with their account on the Manuscript Tracking System (MTS), prior to acceptance. ORCID helps the scientific community achieve unambiguous attribution of all scholarly contributions. You can create and link your ORCID from the home page of the MTS by clicking on ‘Modify my Springer Nature account’. For more information please visit please visit www.springernature.com/orcid.

We look forward to seeing the revised manuscript and thank you for the opportunity to review your work.

Sincerely,

Safia Danovi
Editor
Nature Genetics

Referee expertise:

Referee #1: mutational analysis, computational, cancer genomics

Referee #2: mutational analysis, computational, cancer genomics

Referee #3: mutational analysis, computational, cancer genomics

Reviewers' Comments:

Reviewer #1:
Remarks to the Author:
Summary: In this manuscript by Demeulemeester et al, the authors seek to understand how often biallelic mutations occur in tumor genomes that have a high potential of being missed by variant calling pipelines. They first use simulations based on whole genome data to assess whether these events are likely to be a frequent occurrence, and then use techniques that utilize phasing, variant allelic fraction and purity/CNV information to document four types of biallelic event. They conclude that such events are relatively frequent in tumor genomes, indicate mechanisms associated with higher vulnerability to mutation and recommend frameworks to better detect such mutations from sequencing data. The manuscript is well written and takes a new approach to reanalysis of existing tumor data that expands our current understanding of the mechanisms that are influential in forming the tumor mutational landscape, thus it will likely be of broad interest to the cancer genomics community.

Major comments:

The authors frame their manuscript from the perspective of violations of the infinite sites model. The infinite sites model considers the evolution of a single diploid lineage and assumes that the same site can’t be mutated twice. Given the simplicity of the model, it seems somewhat obvious that there will be violations in tumor genomes. It would help the flow to include a few sentences describing how the infinite sites model assumptions are embedded in the current variant calling approaches that are applied to tumor genomes.

One aspect of the simulations was somewhat unclear. Are the specific mutated positions resampled? Or do the mutated positions simply inform the sampling of a mutation burden and distribution of base contexts, and new sites are sampled at random on each chromosome? A figure describing the simulation, or a simple pseudocode may make help the reader to understand precisely what the simulation is doing.

Following on this, my interpretation of the methods was that the actual mutations were resampled. In that case, is there a bias for violations to occur in tumors with a lower mutation burden in simulation? In practice, and ignoring positive selection, tumors with a higher mutation burden should be more likely to have such an event (indeed, the authors illustrated that the number of violations would scale with the square of the TMB by the birthday approximation). When simulated and observed violations are compared, do they have the same or opposite relation to TMB?

Given that certain sites (e.g. gain of function mutations in certain oncogenes) are recurrently mutated across tumors, would the neighbour resampling simulation be expected to generate a higher number of parallel biallelic events?

It would be helpful to detail the abundance of violations in coding versus non-coding regions. It is clear that phylogenetic studies and studies of mutational signatures benefit from whole genome sequencing (WGS), but WGS remains the exception in clinical settings. What is the potential of the recommended framework for panel-based studies or WXS? Relatedly, is it possible to evaluate the approximate timing of biallelic events using VAF, BAF and phasing information?

Given the approximate random expectation of biallelic events estimated by the authors, can the authors estimate whether these events appear to follow random expectation or could point in some way to positive selection? Their motif enrichment analysis points to sites that are more vulnerable to mutations (more exposed due to frequent TF occupancy, better substrate for POLE or APOBEC3 enzymes) which point to a higher susceptibility of these sites to mutation. In the authors opinion, are biallelic sites informative for identifying functional drivers in cancer, or more helpful for ruling out selection (i.e. recurrence is due to mutability, not selective advantage).

The authors only consider SNVs in their analysis. Would mutational processes and preferences in tumors have the potential to generate biallelic parallel or divergent indels as well? For example, in MSI tumors, recurrent indels and frameshift mutations have been documented.

It was unclear whether the authors plan to make their simulation and biallelic variant detection code available.

Minor comments:

Please include more detailed descriptions of tools used in the methods. What copy number callers and versions were used? What parameter settings were used with each?

Could the authors evaluate whether fragile sites in the genome contribute to biallelic events? Such sites would seem to have a higher-than-average mutation rate, though they may be more relevant to recurrent copy number alterations than SNVs.

Line 28: What is meant by "flagged up in"? Is this a typo?
Line 106: Please clarify "VAF outlying parallel mutations"

Reviewer #2:

Remarks to the Author:
Demeulemeester et al. used whole-genome sequencing data from PCAWG to explore the frequency of violations to the infinite sites model in human cancer. These biallelic mutations are typically filtered in standard variant analysis but the authors do a careful job of detecting them. Two simulation models are used to estimate expected frequencies. They find the biallelic mutation in a substantial fraction (26%) of PCAWG samples. To detect biallelic mutations, the authors use B-allele frequencies as well as phasing of candidate SNVs with adjacent heterozygous SNPs. This phasing idea has been explored in various applications recently and the present study is another clever application. The findings are interesting and the manuscript is well-written overall.

Major comments.
- The authors argue that the violation to the infinite sites model is under-appreciated in previous studies and that it is important to assess the validity of the model. But it is not a “model” in a traditional sense in that no one actually believes it to be true--it is just a simplifying assumption that one often has to make. Furthermore, the violation of this model has been shown beautifully using single cell data by Kuipers et al, Genome Research in 2017 (which the authors cite), so that the novelty of this paper is not so much in showing the violation of the model but in whether such violations can be detected in bulk sequencing data.

- These biallelic mutations are certainly interesting, but the importance of detecting such mutations beyond the theoretical is not clear to me. The authors state that the biallelic mutations could be confounding phylogenetic reconstruction. Is it possible to demonstrating this impact through simulations and, if possible, real-life data? A clear example of how incorporation of these biallelic mutations would give more insights on clonal structure of a tumor would be helpful.

- Parallel mutations are not called in regions with LOH because those mutations are indistinguishable from early mutations. Is it possible that some false positives calls are made due to inaccurate LOH calls? No matter how LOH is called, I would think that a non-trivial segment of the genome is not accurately identified as such.

Minor comments.
- According to Supplemental Table 4, in cases with divergent mutations, they seem to comprise on average 0.01% of the total mutation burden. Do these cases share any clinicobiological traits or is it likely that these mutations constitute passenger events resulting from of a high mutation rate?

- Row 131: The reference to figure 3C should likely point to figure 2C.

- The contents of figure 6d needs to be clarified in some more detail. What do the individual data points represent and what does increased quantum yield mean in this context?

- Supplementary table 3 and 5 are missing data in the chr and position columns.

Reviewer #3:
Remarks to the Author:
Demeulemeester and colleagues report biallelic mutations in cancer whole genome sequencing data which have implications for the validity of some assumptions often used in evolutionary and cancer

genome analysis. With simulation and re-sampling studies they demonstrate that despite the typically small number of substitution mutations found in cancers relative to the size of the genome, their sequence context preferences within a cancer make it reasonably likely that both (diploid) copies of a site could be mutated independently in the same tumour. They go on to show that such sites can be found in publicly available cancer genome sequences based on variant allele frequency (VAF) and careful filtering by several methods. Cases of both parallel mutation (both copies change in the same way) and divergent mutation (the two copies change in different ways) are observed, as would logically be expected, their relative ratio depends on the mutation spectrum of the corresponding tumour. Observed biallelic mutations are typically far more frequent than would be expected based on sampling mutations from similar tumours with closely matched mutation spectra indicating that the analysis of biallelic mutations captures aspects of tumour specific biology and mutation processes that are often not common between "matched" tumours. The authors go on to give examples of extreme hot-spot mutagenesis that correspond to biallelic mutation sites and provide some mechanistic explanations including validation of UV+ETS factor and hairpin+APOBEC hotspots and indicate that the transcription factor NFAT could have similar properties to ETS factors in UV mutagenesis.

This work is important because it highlights that some intuitive expectations about the randomness and chance co-occurrence of mutations at a specific site will often lead to under-estimating the expected frequency of coincidental mutations. It is likely an important part of the explanation for why some genomic sites appear to be mutational hotspots in cancer, and in genomes in general. The manuscript will be of interest to researchers with an interest in mutational processes, mutation spectra, selection under clonal expansion and the broad field of cancer genomics. In terms of scope it would seem a good fit with Nature Genetics.

The study appears well designed and executed. The manuscript is very clearly written with a good narrative and high quality figures. Methods are appropriately detailed though important information is missing from the supplementary files as noted below.

Have a small number of concerns that I would like addressed or clarified before recommending publication.

#1. Framing the work in the context of the infinite sites model is slightly misleading. The two modes of biallelic mutation (parallel and divergent) are not violations of the infinite sites model as there are two copies of each allelic site, each being mutated once, which conforms to infinite sites. Violations in cancer genome analysis under this scenario arise through the artificial treatment of the (approximately) diploid genome as haploid. In contrast the monoallelic forward and backward mutations, which are not the focus of this work, are violations of infinite sites as they mutate the same site multiple times.

#2. The role of selection is not considered in the manuscript. While I accept that the signals reported are almost certainly dominated by mutability, the data are collated from tumours which are subject to positive selection for oncogenic driver mutations. As selection is not excluded as a contributor to biallelic mutations (e.g. additive benefit from biallelic promoter TFBS mutations) it should be at least considered even if ultimately dismissed.

#3. The most striking results for me are those presented in Figure 5c,d. I can envisage wanting to cite this work but would want to give examples of specific cancer types and their corresponding fold-excess of biallelic mutations over expectation from between tumour sampling. However, only two such

figures are vaguely given ("over 8 to 32-fold excess biallelic mutations") and the relate to what is defined as the less accurate method. I'd really like to see the data in these to figure panels plotted as frequency distributions for each cancer type, and a median fold value associated. This value then essentially captures some aspect of the mutational process uniqueness of each tumour in the cancer type.

#4. The study design is essentially a trawl for variants with unusually high VAF that are not present in the non-tumour sample from the same patient, using PCAWG cancer genome sequencing data. Inevitably this will find many alignment problems, copy number alterations, sequence errors and contamination issues. These "noise" signals likely dominate an initial naive screen but the authors have detailed multiple procedures to clean the noise and reveal the signal reported. The correspondence of the biallelic mutations to the predominant mutation signatures for the tumours, and more so to the "simulated violation spectra", along with the SNV-SNP phasing give good confidence that the cleaning has been somewhat successful and there is signal, but it is difficult from the presented results to judge how successful and what fraction of the reported signal remains contaminating noise. Supplementary plots illustrating how such confirmatory metrics change with progressive filtering (or comparisons to simulated data doped with known levels of noise) may help the reader understand this largely hidden but important aspect of the data processing.

#5. Supplementary files. Chromosome identifier and position information are systematically missing from all of the supplementary files. This has precluded reviewer evaluation of data quality for the mutation calls and checking for obvious artefacts in the data. For example checking for pronounced spatial clustering of biallelic mutations or to see if called mutations correspond to known polymorphic sites. The methods report that such human polymorphisms have been filtered but it's a reviewer's prerogative to check.

#6. Figure 2 legend. "Tumour histology of the samples" better described as tumour type.

Author Rebuttal to Initial comments

Point-by-point response to referees

Reviewer #1:

Remarks to the Author:

Summary: In this manuscript by Demeulemeester et al, the authors seek to understand how often biallelic mutations occur in tumor genomes that have a high potential of being missed by variant calling pipelines. They first use simulations based on whole genome data to assess whether these events are likely to be a frequent occurrence, and then use techniques that utilize phasing, variant allelic fraction and purity/CNV information to document four types of biallelic event. They conclude that such events are relatively frequent in tumor genomes, indicate mechanisms associated with higher vulnerability to mutation and recommend frameworks to better detect such mutations from sequencing data. The manuscript is well written and takes a new approach to reanalysis of existing tumor data that expands our current understanding of the mechanisms that are influential in forming the tumor mutational landscape, thus it will likely be of broad interest to the cancer genomics community.

We thank the reviewer for their thorough assessment of our manuscript and hope to have addressed the points raised in our responses below.

Major comments:

The authors frame their manuscript from the perspective of violations of the infinite sites model. The infinite sites model considers the evolution of a single diploid lineage and assumes that the same site can't be mutated twice. Given the simplicity of the model, it seems somewhat obvious that there will be violations in tumor genomes. It would help the flow to include a few sentences describing how the infinite sites model assumptions are embedded in the current variant calling approaches that are applied to tumor genomes.

We agree with the reviewer and have extended the introductory paragraph and rephrased the first section as follows:

> *The infinite sites model of molecular evolution posits that every position in the genome is mutated at most once[1]. By restricting the number of possible mutation histories, haplotypes and alleles, it forms a cornerstone of tumour phylogenetic analysis[2], and is often implied when calling, phasing and interpreting variants[3,4] or studying the mutational landscape as a whole[5]..*
> …
> *In addition, breakdown of the infinite sites assumption at the scale of individual single nucleotide variants (SNVs) was inferred from single-cell tumour sequencing data and flagged as a confounder during phylogenetic reconstruction[8]. In bulk tumour data, population averaging and limited long-range information make it difficult to assess mutational recurrence and its impact on analyses.*

The paragraphs and figure panels provided in response to comment #2 by reviewer #2 further clarify how the infinite sites assumption is embedded in cancer genomics analyses and, specifically, how breakdown affects these:

> *Using single-sample bulk sequencing to establish evolutionary relationships between subclones is challenging[4,23]. Under the infinite sites assumption, one can examine rare pairs of phaseable SNVs in regions without copy number gains[4,24]. Specifically, a pattern where one SNV is only found on a subset of the reads reporting the other evidences a linear relationship (**Figure 6f**). In PCAWG melanomas, however, a median 67% of these pairs in diploid regions reflect phylogenetically uninformative biallelic parallel mutations (**Figure 6g**). To avoid biasing phylogenies, biallelic SNVs should be filtered by restricting analyses to haploid regions or scrutinising the VAF and the likelihood of biallelic mutation in the sample[4]. SNV clustering approaches, which rely on the infinite sites assumption for subclonal reconstruction and assignment of each variant to a specific*

*lineage, may pick up "superclonal clusters" of biallelic parallel mutations, but are otherwise expected to remain robust at the levels identified here (**Figure 6h**)[23].*

*Phasing is also used to boost the accuracy of variant callers for single molecule sequencing data[25]. As with multi-allelic variants, relaxation of the set of allowed haplotypes will need to be considered to capture the full extent of somatic variation. Indeed, while only 2.8% of biallelic hits fall within or near exons, we identify 8 candidate biallelic driver events. Parallel nonsense mutations in tumour suppressors ASXL2 and CDKN2A, and divergent events in ERBB4, suggest that in rare cases, biallelic mutations are selected for (**Figure 6i**, **Table S7**).*

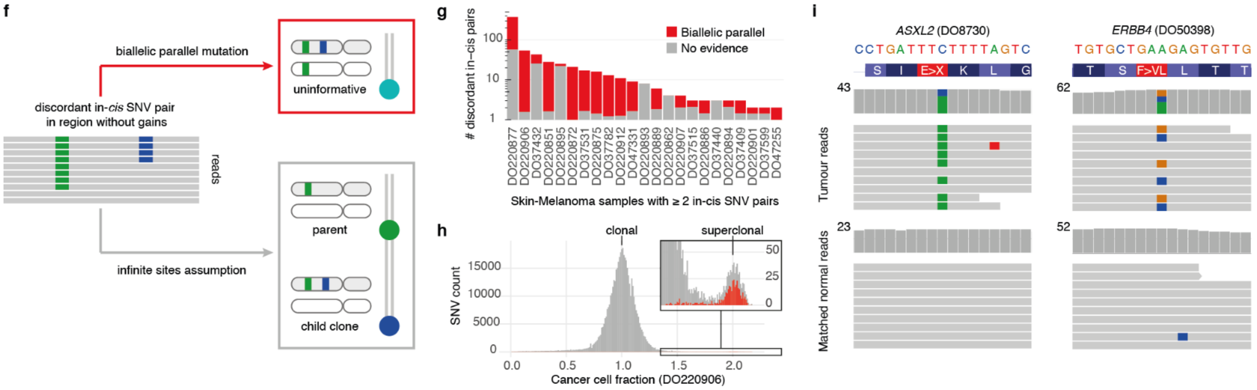

Figure 6 | Biallelic mutations reveal hotspot motifs and confound phylogenies
*… (**f**) Patterns of in-cis SNV pairs in a diploid region evidence linear phylogenies (parent-child) when the infinite sites assumption holds. (**g**) Bar plot showing the number of in-cis SNV pairs in PCAWG melanoma samples with at least two such pairs. Bar height reflects total numbers observed while the red portion indicates the fraction of all pairs with evidence for biallelic parallel mutation (beta-binomial p-value ≤ 0.05 or phasing to a heterozygous SNP). (**h**) Histogram of cancer cell fractions of SNVs in melanoma DO220906. The clonal cluster and a superclonal cluster containing mainly biallelic parallel mutations (red), are indicated. (**i**) IGV visualisation of two missed biallelic drivers in colorectal and oesophageal adenocarcinomas DO8730 and DO50398, respectively. Reads (horizontal bars) are downsampled for clarity and local base-wise coverage is indicated left of the histograms.*

One aspect of the simulations was somewhat unclear. Are the specific mutated positions resampled? Or do the mutated positions simply inform the sampling of a mutation burden and distribution of base contexts, and new sites are sampled at random on each chromosome? A figure describing the simulation, or a simple pseudocode may make help the reader to understand precisely what the simulation is doing.

We apologise for the confusion. We have in fact implemented both options mentioned by the reviewer as two distinct simulation approaches. In the first approach (uniform permutation model) we merely use the observed mutations in a tumour to inform the mutation burden and mutation spectrum and then randomly resample corresponding mutations across a callable diploid genome. In the second approach (neighbour resampling model), we resample the specific positions and their corresponding mutations: we randomly pick 50% of the observed mutations in the target tumour and resample, without replacement, the other 50% from similar PCAWG tumours (*i.e.*, same tumour type and a similar mutation spectrum). In the revised manuscript, we have expanded the relevant methods sections and have included a novel **Figure S1** to clarify these two models, and their distinction, for the readers.

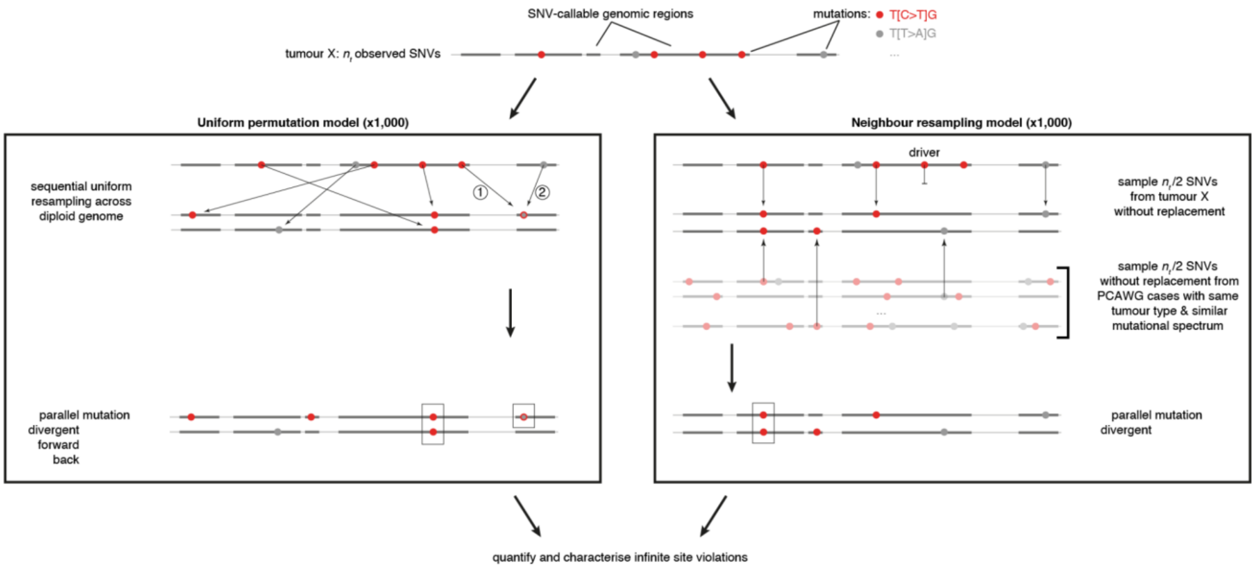

Figure S1 | Simulation approaches for infinite sites model violations
Schematic overview of the uniform permutation (left) and neighbour resampling (right) approaches to assess the number and type of infinite sites violations expected in a tumour. Numbers in the uniform permutation panel highlight the sequential nature of the sampling, which keeps track of mutated positions to consider them accordingly for biallelic, forward, and back mutation. Note that the neighbour resampling model excludes all PCAWG annotated driver mutations and allows simulation of biallelic events only.

The methods section on the neighbour resampling model now reads:

In a second simulation approach (termed neighbour resampling model), we resample without replacement the mutational landscape of a tumour from the empirical mutation distribution, minus the annotated driver SNVs (Synapse ID: syn11639581 and

https://dcc.icgc.org/releases/PCAWG/driver_mutations/). Specifically, in each simulation, we randomly pick 50% of the observed mutations in the original tumour and resample the other 50% from the pooled SNVs of representative PCAWG tumours. In this context, we define a tumour as representative for the simulation target when it is of the same tumour type (PCAWG histology) and has similar mutational signature exposures (cosine similarity of their mutation spectra ≥ 0.9)[11]. This can be viewed as sampling one allele from the original tumour and one allele from the corresponding empirical mutation distribution. Note that the approach allows to simulate biallelic events but not back and forward mutation and can be applied only to tumours with a representative SNV pool at least 0.5 times their total mutation burden. We further exclude all graylisted and non-preferred multi-sample tumours[13] as well as 21 prostate cancer cases from the PRAD-CA cohort which were suspected of contamination harbouring excess low VAF SNV calls in repetitive regions.

Following on this, my interpretation of the methods was that the actual mutations were resampled. In that case, is there a bias for violations to occur in tumors with a lower mutation burden in simulation? In practice, and ignoring positive selection, tumors with a higher mutation burden should be more likely to have such an event (indeed, the authors illustrated that the number of violations would scale with the square of the TMB by the birthday approximation). When simulated and observed violations are compared, do they have the same or opposite relation to TMB?

We hope our answer above has made the simulation approaches more clear. We assume the bias the reviewer refers to is the one that would arise in the neighbour resampling model when drawing the same mutation multiple times from tumours with a low mutation burden. To exclude this possibility, resampling is always performed without replacement.

In **Figure 5c**, we highlight the accuracy of the neighbour resampling approach in estimating the number of observed biallelic mutations across PCAWG, including cases with mutation burdens as low as 773 and as high as 2,434,624. In **Figure 5d** we subsequently use this confirmed model to simulate a range of mutation burdens for each tumour type, demonstrating that also the simulated number of biallelic mutations scales with the square of the tumour mutation burden across this range.

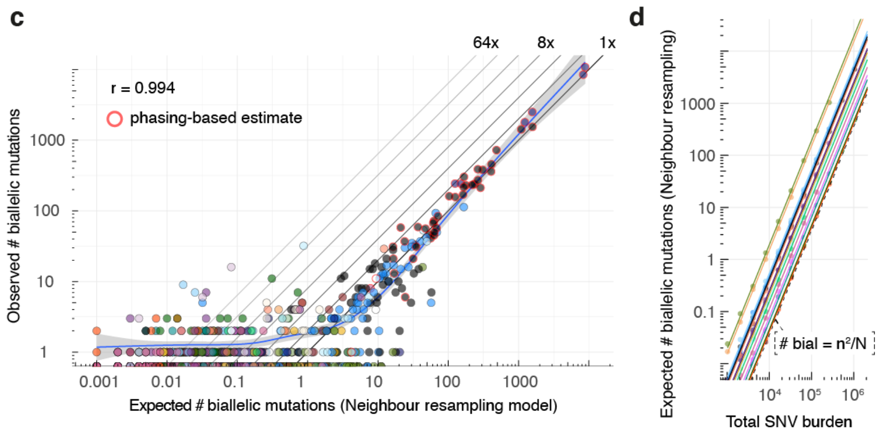

Figure 5 | Comparison between observed and simulated biallelic mutations
*… (**c**) Scatterplot of the observed vs. neighbour resampling model-expected number of biallelic mutations (parallel + divergent) for all PCAWG tumours. For cases with ≥10,000 phaseable SNVs (red borders), the phasing-based number is provided. Colours reflect tumour type as in **Figure 2**. A spline regression fit is shown together with the Pearson correlation. (**d**) Number of biallelic violations expected according to the neighbour resampling model for a range of mutation burdens and tumour types. The dashed line indicates the birthday problem estimate equal to the square of the mutation burden divided by the genome size (m^2/N). Full coloured lines are the linear fits per tumour type.*

The corresponding methods description (extending the neighbour resampling section) reads:

In a second simulation approach (termed neighbour resampling model), we resample without replacement the mutational landscape of a tumour from the empirical mutation distribution, minus the annotated driver SNVs (Synapse ID: syn11639581 and https://dcc.icgc.org/releases/PCAWG/driver_mutations/). Specifically, in each simulation, we randomly pick 50% of the observed mutations in the original tumour and resample the other 50% from the pooled SNVs of representative PCAWG tumours. In this context, we define a tumour as representative for the simulation target when it is of the same tumour type (PCAWG histology) and has similar mutational signature exposures (cosine similarity of their mutation spectra ≥ 0.9)[11]. This can be viewed as sampling one allele from the original tumour and one allele from the corresponding empirical mutation distribution.
…
When simulating a range of mutational burdens for a tumour type, we leverage the tumour with the highest number of representative tumours (≥ 10) and with a total number of mutations to sample from exceeding 100,000. In this case, all mutations are sampled from the pooled set of mutations, which in this case includes the original tumour.

Given that certain sites (e.g. gain of function mutations in certain oncogenes) are recurrently mutated across tumors, would the neighbour resampling simulation be expected to generate a higher number of parallel biallelic events?

We thank the reviewer for this insightful comment and have assessed the effect of driver SNVs on the neighbour resampling simulations. **Figure R1** shows the estimated number of biallelic mutations in PCAWG samples according to the original simulations *vs.* new simulations in which all annotated driver point mutations were excluded. Simulation results are highly similar between the two sets, with the exception of pancreatic adenocarcinoma where we consistently overestimated the number of biallelic mutations by a median ~0.1 per sample (0.133 *vs.* 0.037 after excluding drivers). This is explained by an extreme prevalence of *KRAS* G12 mutations in PCAWG pancreatic adenocarcinomas (57% and 20% of cases are mutated at chr12:25,398,284 and chr12:25,398,285, respectively) together with the relatively low total mutation burden of the cohort (median 5,017 SNVs per tumour). Note that no *KRAS* G12 mutations were picked up as true biallelic hits by the phasing or VAF-based pipelines.

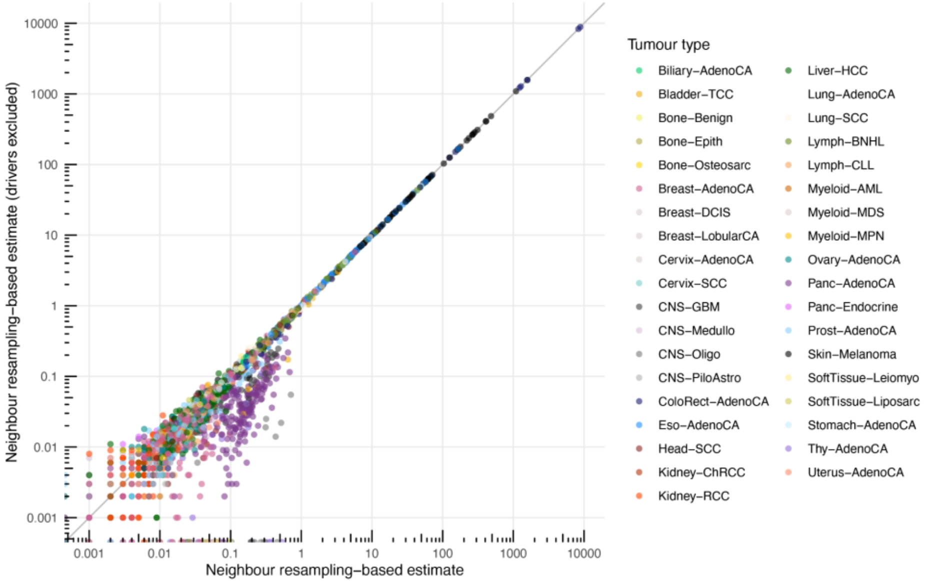

Figure R1 | Comparison of neighbour resampling results with and without driver SNVs. Scatter plot of the estimated number of biallelic mutations according to the neighbour resampling model before (x-axis) and after (y-axis) excluding all annotated driver SNVs.

To eliminate this bias, we now consistently exclude all PCAWG annotated driver point mutations from the neighbour resampling simulations throughout the manuscript and have amended the manuscript

accordingly in the following places. Note that this has not affected any of our findings and that the analyses in **Figure S7** (showing that loci with biallelic mutations have higher intrinsic mutability) had already excluded annotated driver loci.

The main text highlights the exclusion of driver mutations from the neighbour resampling model:

> *A second simulation approach, resampling (without replacement, non-driver) mutations from tumours of the same cancer type with similar mutational signature activities, confirms these observations (neighbour resampling model;* ***Figure 2b, S1, Table S2****).*

This is clarified in the legend of the novel **Figure S1** as well:

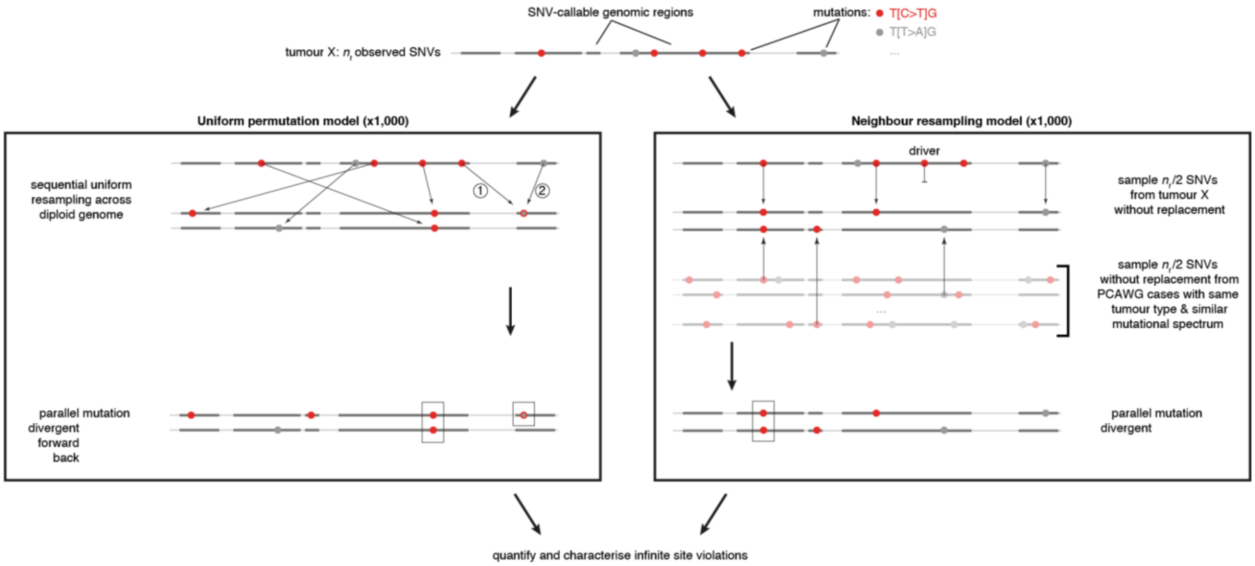

Figure S1 | Simulation approaches for infinite sites model violations
Schematic overview of the uniform permutation (left) and neighbour resampling (right) approaches to assess the number and type of infinite sites violations expected in a tumour. Numbers in the uniform permutation panel highlight the sequential nature of the sampling, which keeps track of mutated positions to consider them accordingly for biallelic, forward, and back mutation. Note that the neighbour resampling model excludes all PCAWG annotated driver mutations and allows simulation of biallelic events only.

The methods section further details the removal of the PCAWG annotated driver SNVs from the consensus variants:

In a second simulation approach (termed neighbour resampling model), we resample without replacement the mutational landscape of a tumour from the empirical mutation distribution, minus the annotated driver SNVs (Synapse ID: syn11639581 and https://dcc.icgc.org/releases/PCAWG/driver_mutations/).

Figures 2b–c, **5c–e** and **S6b** have all been updated (or represent novel panels) to reflect the new simulation results:

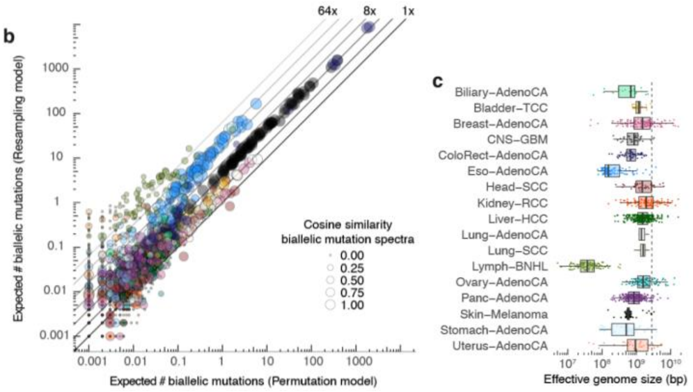

Figure 2 | Simulated landscape of infinite sites violations in the PCAWG cohort

*… (**b**) Comparison of the expected biallelic violations from the uniform permutation and neighbour resampling models. Every dot represents a tumour simulated 1,000x with each model. Colour and size reflect, respectively, tumour type and the cosine similarity of the predicted biallelic mutation spectra. (**c**) Box and scatterplot showing the effective genome size perceived by the mutational processes per cancer type, as estimated from the per-sample differences between simulation approaches. The dashed line indicates the callable genome size. The effective genome size is smallest in Lymph-BNHL (~37Mb), driven by recurrent focal hypermutation[13]. Centre line, median; box limits, upper and lower quartiles; whiskers, 1.5x interquartile range.*

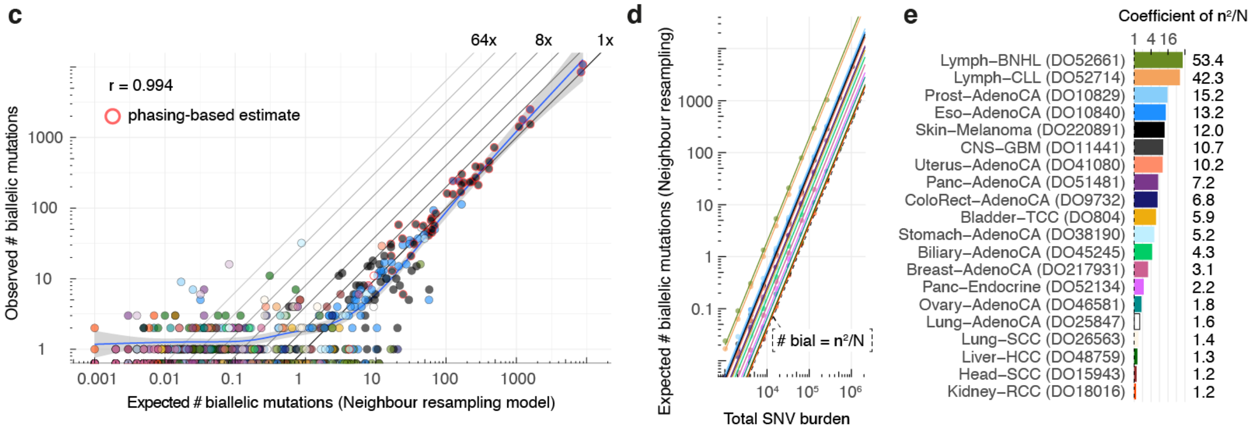

Figure 5 | Comparison between observed and simulated biallelic mutations

*… (**c**) Scatterplot of the observed vs. neighbour resampling model-expected number of biallelic mutations (parallel + divergent) for all PCAWG tumours. For cases with ≥10,000 phaseable SNVs (red borders), the phasing-based number is provided. Colours reflect tumour type as in **Figure 2**. A spline regression fit is shown together with the Pearson correlation. (**d**) Number of biallelic violations expected according to the neighbour resampling model for a range of mutation burdens and tumour types. The dashed line indicates the birthday problem estimate equal to the square of the mutation burden divided by the genome size (m^2/N). Full coloured lines are the linear fits per tumour type. (**e**) Bar plot of the fitted coefficients of m^2/N as derived in (d). For each tumour type, the ICGC donor ID indicates the representative tumour used.*

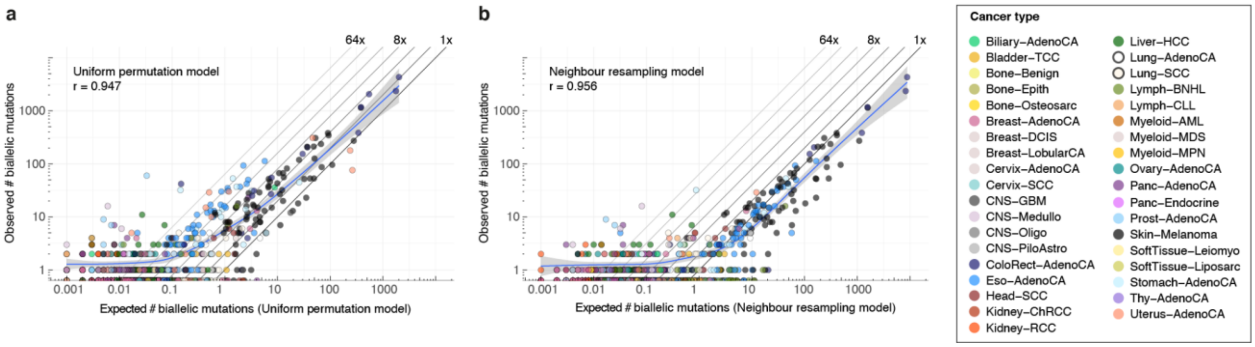

Figure S6 | Comparison between observed and simulated biallelic mutations

*(**a**,**b**) Scatterplots of the observed vs. expected number of biallelic mutations (parallel + divergent) for all PCAWG tumours using the uniform permutation (**a**) and neighbour resampling models (**b**). A spline regression fit is shown together with the Pearson correlation.*

It would be helpful to detail the abundance of violations in coding versus non-coding regions. It is clear that phylogenetic studies and studies of mutational signatures benefit from whole genome sequencing (WGS), but WGS remains the exception in clinical settings. What is the potential of the recommended framework for panel-based studies or WXS?

We have now assessed the frequency of biallelic mutations in or near coding regions. The results are included in a new paragraph together with our finding of missed candidate biallelic driver events, highlighting unique insights that can be gained when applying our framework to panel-based studies.

> *... Indeed, while only 2.8% of biallelic hits fall within or near exons, we identify 8 candidate biallelic driver events. Parallel nonsense mutations in tumour suppressors ASXL2 and CDKN2A, and divergent events in ERBB4, suggest that in rare cases, biallelic mutations are selected for (**Figure 6i**, **Table S7**).*

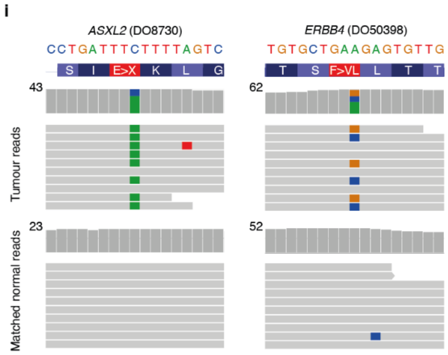

Figure 6 | Biallelic mutations reveal hotspot motifs and confound phylogenies
*... (**i**) IGV visualisation of two missed biallelic drivers in colorectal and oesophageal adenocarcinomas DO8730 and DO50398, respectively. Reads (horizontal bars) are downsampled for clarity and local base-wise coverage is indicated left of the histograms.*

Relatedly, is it possible to evaluate the approximate timing of biallelic events using VAF, BAF and phasing information?

Unfortunately, a general sparsity of phasing information and the effect of biallelic mutations on the VAF make it difficult to distinguish early and late clonal as well as sublonal biallelic events from a single tumour sample. For instance, biallelic parallel events affecting two copies of one parental allele would be indistinguishable from clonal early mutations, *i.e.*, those which occurred prior to the duplication of the allele.

The resolution of timing analyses is greatly enhanced however in multi-sample studies. We hence compiled biallelic parallel events in a cohort of metastatic prostate cancer with sequencing of matched primary and metastases included in PCAWG (Gundem et al., *Nature*, 2015). The analysis suggests ongoing accumulation of biallelic parallel events during tumour evolution, with several early clonal (preceding the evolution of the most recent common ancestor) as well as candidate late and subclonal events, which are absent from or likely monoallelic in one or multiple samples. These considerations and findings are included in our revised manuscript as follows.

> *Without phasing information, we can only detect parallel mutations on more copies than the major allele tumour copy number. Hence, no parallel mutations are called in regions with loss of heterozygosity and late or subclonal events are likely to be underrepresented. Insights into the latter can be glimpsed from multi-sample studies. In a cohort of metastatic prostate cancer with sequencing of matched primary and metastases[12,13], we discern early clonal (preceding the most recent common ancestor) as well as candidate late and subclonal events (**Figure S4**).*

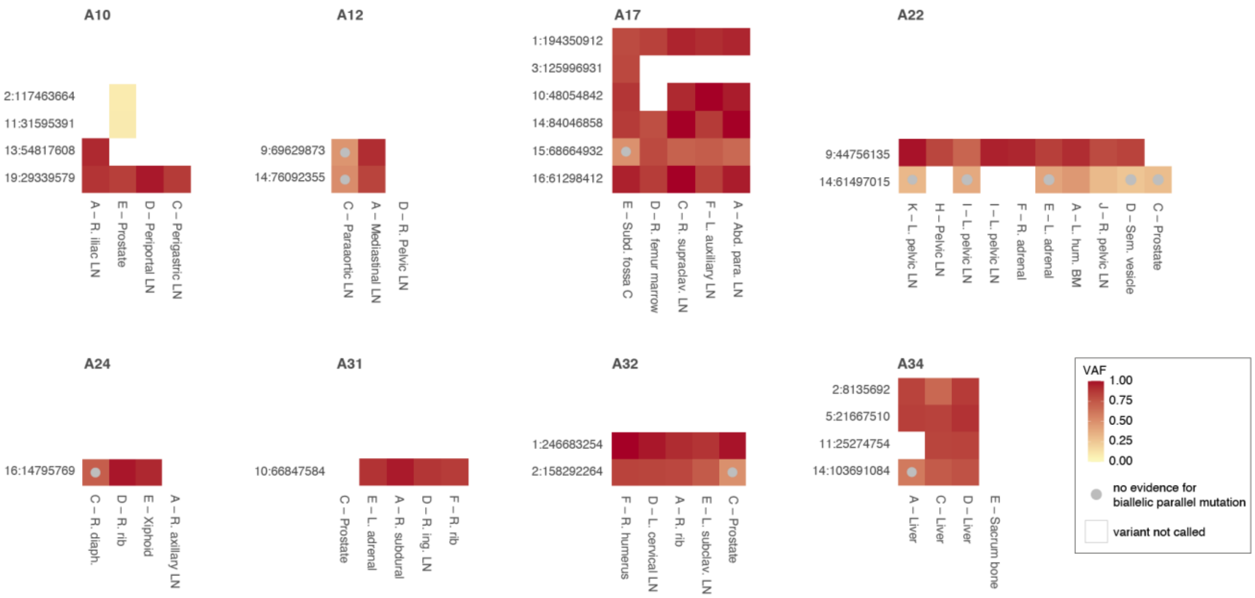

Figure S4 | Biallelic parallel mutation during metastatic prostate cancer evolution
Heatmap showing allele frequencies of variants found to be biallelic in at least one sample of eight prostate cancers with sequencing of matched primary and metastases (A10–A34, different sites indicated as in Gundem et al.[12]). Early clonal biallelic mutations are detected in all samples of a patient (e.g., A10 chr19:29,339,579), while late clonal and subclonal ones show no evidence of being biallelic in some samples (beta-binomial p-value > 0.05 and no discordant phasing to a heterozygous germline SNP) or are detected in only a subset of samples (e.g., A22 chr14:61,497,015 and A10 chr2:117,463,664, respectively).

Given the approximate random expectation of biallelic events estimated by the authors, can the authors estimate whether these events appear to follow random expectation or could point in some way to positive selection? Their motif enrichment analysis points to sites that are more vulnerable to mutations (more exposed due to frequent TF occupancy, better substrate for POLE or APOBEC3 enzymes) which point to a higher susceptibility of these sites to mutation. In the authors opinion, are biallelic sites informative for identifying functional drivers in cancer, or more helpful for ruling out selection (i.e. recurrence is due to mutability, not selective advantage).

While biallelic mutations may be selected for in rare cases, such as the 8 candidate drivers highlighted in our response above, the bulk of the signal is indeed more likely to be explained by mutability. As our data are all collated from tumours, it is difficult however to establish a realistic random model that does not carry a signature of selection. Nevertheless, the correlation with empirical locus mutability across cancer types (**Figure S7**), the accuracy of the simulations in recapitulating both the spectrum and number of biallelic events (**Figure 5a–c**), the absence of recurrent biallelic mutations in coding regions (**Figure 6a**), the motif enrichment results highlighted by the reviewer (**Figure 6a–d**), as well as new results showing no discernible transcriptional impact of mutations at the identified promoter loci in melanoma (**Figure 6a, S9**, included below), all support this notion. A take-home message from our manuscript should be that the, at times extreme (*e.g.*, ETS/NFAT/APOBEC recognition sites), local mutation rate determinants should be accounted for when making inferences (*e.g.*, driver calling, phylogenies) based on tumour mutation data.

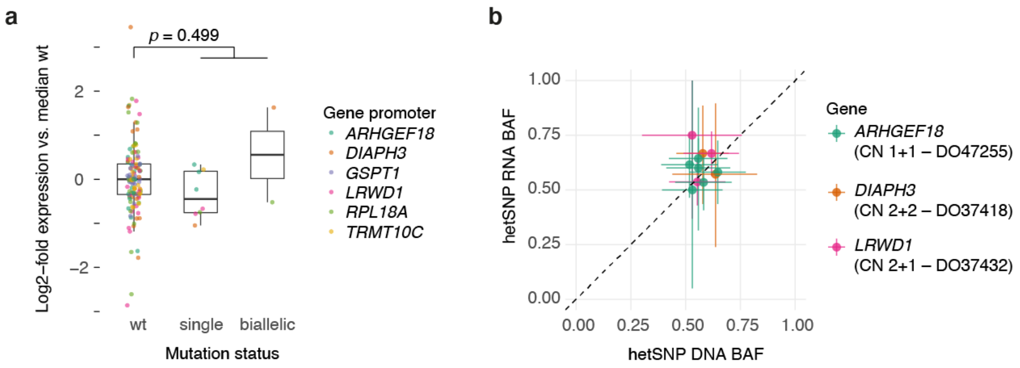

Figure S9* | *Effect of promoter mutation on gene expression for genes with biallelic hits
*(**a**) Box and scatter plot showing the log2-fold change in expression compared to the median wild type for promoter mutated genes in **Figure 6a**. Each dot represents the relative expression in a single PCAWG melanoma with RNA-Seq data, stratified by the mutation status of that gene's promoter. (**b**) Scatter plot with posterior 95% highest density intervals for the DNA and RNA B-allele frequencies of expressed germline heterozygous SNPs in the genes/samples with a single mutant promoter allele in (a). The ICGC donor ID and local consensus copy number are indicated.*

These results are summarised in the text as follows:

> *While we cannot formally exclude selection as a contributor to these recurrent mutations, no effects on total or allele-specific expression of genes with biallelic promoter mutations could be observed (**Figure S9**).*

The methods section has also been updated to describe these analyses:

> ***Gene expression analysis***
> *We obtained gene expression data from PCAWG (Synapse ID syn5553991; http://dcc.icgc.org/releases/PCAWG/transcriptome/gene_expression/)[28]. Briefly, reads were aligned with both TopHat2 (v.2.0.12) and STAR (v.2.4.0i, two-pass). Read counts for genes were calculated using htseq-count and the GENCODE v19 annotation. Counts were normalized using FPKM and upper quartile normalization: i.e. FPKM-UQ* $= (C \times 10^9)/(UQ \times L \times G)$ *where C is the fragment count, L the length of the gene, UQ the upper quartile of the fragment count in protein coding genes on autosomes and G the number of such protein coding genes. The final expression values are an average of the TopHat2 and STAR-based alignments. FPKM-UQ values for genes with recurrent (biallelic) promoter mutations in melanoma were extracted and stratified by promoter mutation status in the tumour (wild type, single SNV, biallelic mutation). Per gene, log2 fold change values were computed compared to the median expression from the wild type promoter. To assess whether single SNVs at these promoter sites induce allele-specific expression, we used RSamtools to pile up base counts from the STAR-aligned bam files at the locations of heterozygous germline SNPs in the patient as identified from the matching normal genome by Battenberg. Posterior 95% highest density intervals were computed for the DNA and RNA base counts assuming a uniform* $\mathtt{Beta}(1,1)$ *prior and a binomial likelihood. Non-overlapping intervals can be indicative of allele-specific expression.*

The authors only consider SNVs in their analysis. Would mutational processes and preferences in tumors have the potential to generate biallelic parallel or divergent indels as well? For example, in MSI tumors, recurrent indels and frameshift mutations have been documented.

We thank the reviewer for this intriguing question and have implemented a modified version of our neighbour resampling simulator to assess the number of biallelic parallel and divergent indels which may reasonably be expected in PCAWG tumours. As the reviewer hinted at, we identify four microsatellite unstable tumours which are likely to boast several hundreds to over one thousand, mostly parallel, biallelic indels. These mainly originate from indel signatures 1 and 2, reflecting slippage during DNA replication and subsequent 1bp T (or A) insertion and deletion in thymine (adenosine) mononucleotide repeats,

respectively. These novel simulations and results are now included in the revised manuscript as detailed below.

> *A second simulation approach, resampling (without replacement, non-driver) mutations from tumours of the same cancer type with similar mutational signature activities, confirms these observations (neighbour resampling model;* ***Figure 2b, S1, Table S2****). In addition, this approach indicates four microsatellite unstable tumours harbour hundreds of parallel biallelic indels (****Figure S2****).*

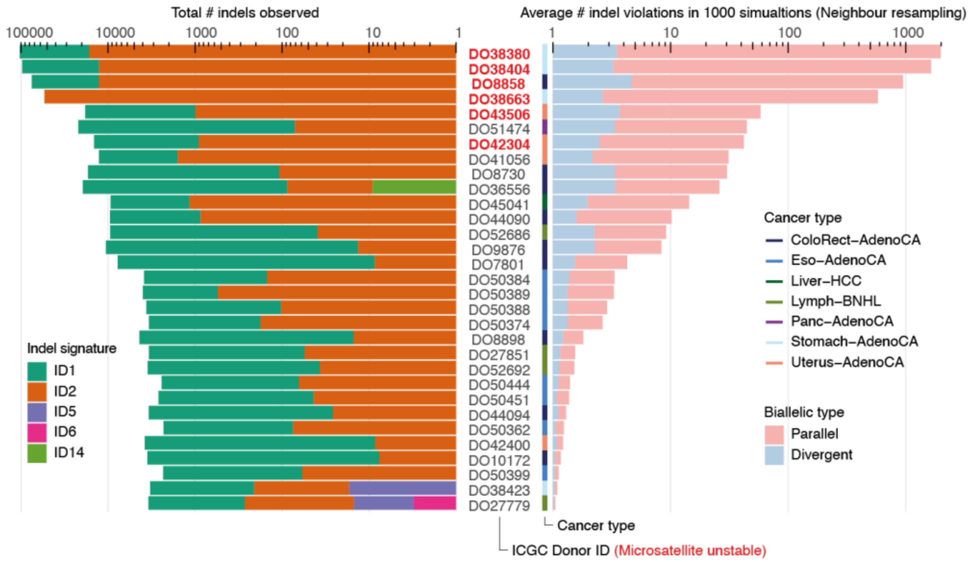

Figure S2 | Biallelic indels are expected in a subset of microsatellite unstable tumours
Bar plots of the observed indel burden and signature (left) and the expected biallelic indels according to the neighbour resampling model (right). Bar height indicates total numbers and coloured subdivisions represent fractions contributed by each indel signature (left) or biallelic indel type (right). Only PCAWG tumours with ≥1 expected biallelic indel are shown. Four microsatellite unstable tumours are predicted to boast several hundreds to over one thousand, mostly parallel, biallelic indels. These mainly originate from indel signatures 1 and 2, likely reflecting slippage during DNA replication and subsequent 1bp T (or A) insertion and deletion in thymine (adenosine) mononucleotide repeats, respectively.

With the corresponding methods section reading:

> *The neighbour resampling approach was also applied to indels, in which case the exact same pipeline described above could be followed, using indels instead of SNVs. To identify representative tumours, we used the PCAWG indel signatures (ID1–17) and their exposures in each of the*

samples[11]. Microsatellite instability classification of all PCAWG tumours was obtained from Fujimoto et al.[27].

It was unclear whether the authors plan to make their simulation and biallelic variant detection code available.

We apologise for this omission and have now included a link to the GitHub repository containing our code under Code availability in the Online methods section our manuscript:

Code availability
All R code used for simulation and biallelic variant detection is available at https://github.com/jdemeul/InfiniteSites.

Minor comments:

Please include more detailed descriptions of tools used in the methods. What copy number callers and versions were used? What parameter settings were used with each?

We have added further details to the Online methods section. Specifically, the "Consensus copy number, purity and ploidy" section has been extended and reports the copy number callers that were used, a comprehensive description of how the consensus copy number, purity and ploidy were constructed from the individual calls, and links to where the data can be obtained. The section now reads as follows:

Consensus copy number, purity and ploidy
PCAWG consensus copy number profiles, tumour purity and ploidy, were obtained from Dentro et al.[4,13], available from Synapse with accession number syn8042880 and the ICGC Data Portal at http://dcc.icgc.org/releases/PCAWG/consensus_cnv/. Briefly, each cancer's genome was first segmented into regions of constant copy number using six individual copy number callers: ABSOLUTE, ACEseq, Battenberg, cloneHD, JaBbA and Sclust, run as detailed in Dentro et al.[4]. Consensus segment breakpoints were determined from the PCAWG consensus structural variants (SVs; available via Synapse syn7596712 and at http://dcc.icgc.org/releases/PCAWG/consensus_sv/) complemented with high-confidence breakpoints identified by several of the copy number callers. The six callers were then re-run, enforcing this consensus segmentation as well as separately established consensus tumour ploidy, which was typically obtained by resolving disagreement on whether a whole genome duplication had occurred by an expert panel[4]. The allele-specific copy number calls were subsequently

combined into a consensus profile by looking, for each segment, at the agreement in major and minor allele copy number states between the output of the six callers. Lastly, consensus was obtained on tumour purity by combining the calls from the six copy number callers with those from subclonal architecture reconstruction methods that leverage SNV data: CliP, CTPsingle, PhyloWGS, cloneHD and Ccube, as detailed in Dentro et al.[4]. The whole multi-tiered approach yielded a purity estimate for every PCAWG tumour and a copy number call for every consensus segment, annotated with a confidence level between and a quality star. Levels a, b and c (3 stars) are the strictest and require that at most one method disagrees. Segments for which a majority agrees on the clonal copy number are assigned 2 stars (levels d, e, f). The remaining levels (g, h, i) are marked 1 star to denote the lowest confidence.

Could the authors evaluate whether fragile sites in the genome contribute to biallelic events? Such sites would seem to have a higher-than-average mutation rate, though they may be more relevant to recurrent copy number alterations than SNVs.

As fragile sites are often tissue-type specific, we used the PCAWG somatic structural variant (SV) breakpoints as a relevant proxy. For each tumour, we determined the distance from all biallelic and normal SNVs to the nearest breakpoint. We subsequently tested whether biallelic mutations were more closely associated with SVs than normal SNVs using a one-sided Mann-Whitney *U*-test. No tumours showed significant association after Benjamini–Hochberg correction for multiple testing (p-values ≥ 0.311), also when subsetting the analysis to cases with 5 or more biallelic events (p-values ≥ 0.088). This is reported in the manuscript as follows:

*Biallelic mutations are not associated with somatic rearrangements ($p_{adj} \geq 0.31$; Mann-Whitney U-test, Benjamini–Hochberg) but occur at loci with a higher mutation rate (**Figure S7**), some of which harbour recurrent biallelic events (**Figure 6a**).*

We would like to thank the reviewer for this extremely helpful comment, as it pointed us to an enrichment of likely false positive VAF-pipeline hits (few with phasing support) in regions with common germline structural variants – notably deletions. This was turned into a filter which has further increased the accuracy of our biallelic variant calls and improved cosine similarities and correlations in **Figure 5a,c**. The filter description is provided in the methods and its effect is illustrated in the novel **Figure S3a,b** also included below.

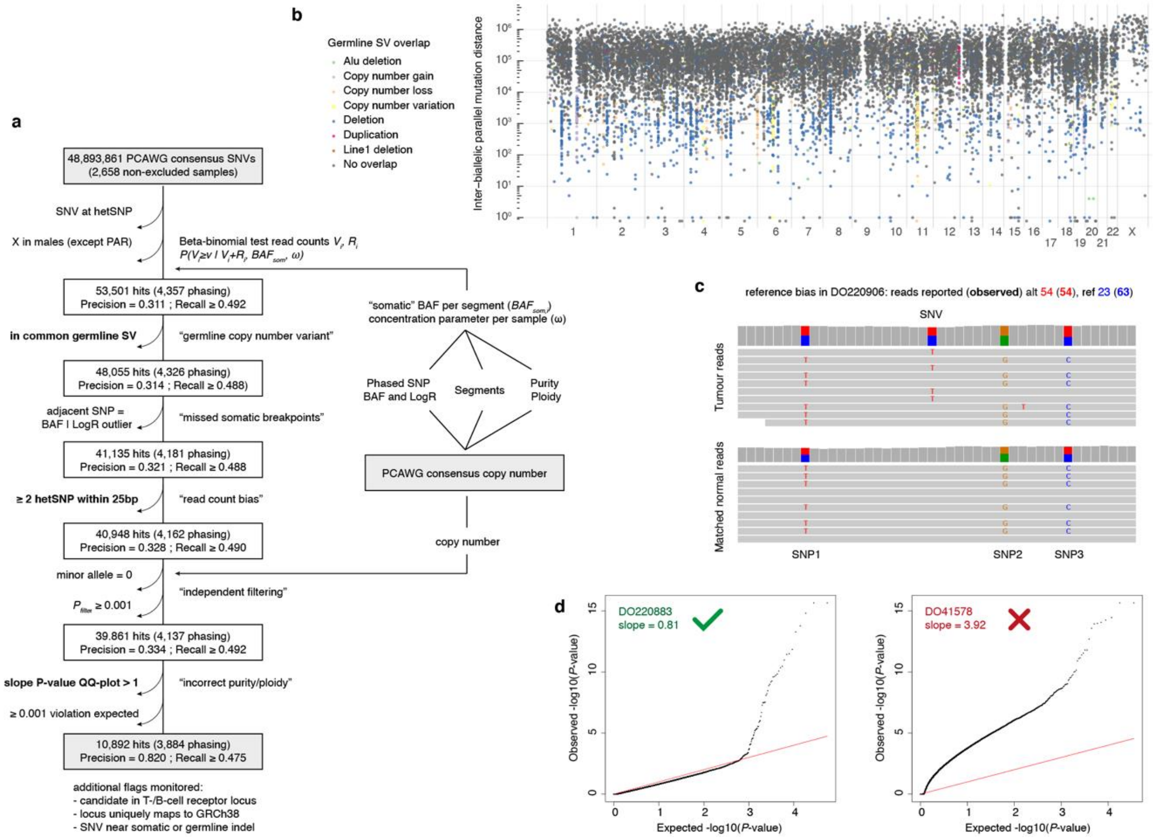

Figure S3 | Detection of biallelic parallel mutations by allele frequency

*(**a**) Flow chart showing the filtering steps, phasing-based estimates of precision and lower bound recall, as well as the input and output data for our pipeline to detect biallelic parallel mutations in PCAWG based on variant allele frequencies (VAF). Three filtering steps highlighted in bold are further illustrated in panels (b-d). (**b**) Rainfall plot of all biallelic parallel hits obtained after omitting the germline SV filter. Streaks of coloured dots indicate a clustering of hits in regions with common germline structural variants. While demonstrating the ability of the pipeline to detect VAF outliers, these hits are poorly supported by phasing data and likely represent single somatic SNVs in the context of a heterozygous germline deletion. (**c**) Example of reference bias in the PCAWG consensus SNV read counts. Reads carrying the somatic variant contain alternate germline alleles at three proximal positions, resulting in an underreporting of the number of wild type reads and an overestimation of the VAF. (**d**) Diagnostic QQ-plots of the one-sided beta-binomial read count test P-values for two samples. DO41578 P-values are overinflated (slope > 1), hinting at consensus purity/ploidy errors, and the sample is excluded as a result.*

With details in the methods section:

Several additional quality checks and filters are in place to mitigate effects of potential errors and biases in allele counts, consensus genome segmentation, purity and ploidy:

...

ii. Candidate variants are filtered when they reside in a region of common structural variation as listed in nstd186 (NCBI Curated Common SVs – all populations from 1000 Genomes; allele frequency ≥ 0.01).

Line 28: What is meant by "flagged up in"? Is this a typo?

This has been amended:

In addition, breakdown of the infinite sites assumption at the scale of individual single nucleotide variants (SNVs) was inferred from single-cell tumour sequencing data and flagged as a confounder during phylogenetic reconstruction[8].

Line 106: Please clarify "VAF outlying parallel mutations"

This has been simplified to:

*Parallel mutations confirmed by phasing are found in tumours with as few as 8,892 SNVs while divergent mutations are repeatedly identified in oesophageal adenocarcinomas with 20,000-30,000 SNVs (**Figure 4**).*

Reviewer #2:
Remarks to the Author:
Demeulemeester et al. used whole-genome sequencing data from PCAWG to explore the frequency of violations to the infinite sites model in human cancer. These biallelic mutations are typically filtered in standard variant analysis but the authors do a careful job of detecting them. Two simulation models are used to estimate expected frequencies. They find the biallelic mutation in a substantial fraction (26%) of PCAWG samples. To detect biallelic mutations, the authors use B-allele frequencies as well as phasing of candidate SNVs with adjacent heterozygous SNPs. This phasing idea has been explored in various applications recently and the present study is another clever application. The findings are interesting and the manuscript is well-written overall.

We are grateful to the reviewer for their appraisal of our work and have provided a point-by-point response to the comments raised below.

Major comments.
- The authors argue that the violation to the infinite sites model is under-appreciated in previous studies and that it is important to assess the validity of the model. But it is not a “model” in a traditional sense in that no one actually believes it to be true--it is just a simplifying assumption that one often has to make. Furthermore, the violation of this model has been shown beautifully using single cell data by Kuipers et al, Genome Research in 2017 (which the authors cite), so that the novelty of this paper is not so much in showing the violation of the model but in whether such violations can be detected in bulk sequencing data.

We wholeheartedly agree with the reviewer and have rephrased the first paragraph as follows to better reflect this point:

> *Recent studies have shown systematic variation in mutation rates across the genome, resulting in specific hotspots[5–7]. In addition, breakdown of the infinite sites assumption at the scale of individual single nucleotide variants (SNVs) was inferred from single-cell tumour sequencing data and flagged as a confounder during phylogenetic reconstruction[8]. In bulk tumour data, population averaging and limited long-range information make it difficult to assess mutational recurrence and its impact on analyses.*

In addition, we now use the phrasing “infinite sites assumption” rather than infinite sites model at various locations in the manuscript.

- These biallelic mutations are certainly interesting, but the importance of detecting such mutations beyond the theoretical is not clear to me. The authors state that the biallelic mutations could be confounding

phylogenetic reconstruction. Is it possible to demonstrating this impact through simulations and, if possible, real-life data? A clear example of how incorporation of these biallelic mutations would give more insights on clonal structure of a tumor would be helpful.

We have extended **Figure 6** and the corresponding text with analyses highlighting insights which can be gained and biases avoided by detecting biallelic mutations. Most notably, we demonstrate how biallelic parallel mutations confound phylogenetic inference in the PCAWG melanoma data. Briefly, the following items are discussed:

1) At-scale detection of biallelic variants increases our understanding of mutational processes. (This part was present in the original manuscript; **Figure 6a-e**.)
2) Biallelic hits are a direct proxy for parallel mutations between lineages (**Figure S10**) and set expectations for the infinite sites violations to allow during phylogenetic analysis.
3) SNV pair phasing patterns in regions without copy number gains are commonly used to establish evolutionary relationships between clones. In PCAWG melanomas 67% of pairs suggesting a linear relationship (parent-child) are in fact due to phylogenetically uninformative biallelic parallel mutations (**Figure 6f-g**).
4) Biallelic parallel mutations can contribute so-called "superclonal clusters" in SNV clustering approaches for subclonal reconstruction (**Figure 6h**).
5) Relevant cancer drivers may be missed when biallelic mutations are not considered or filtered during variant calling (**Figure 6i**).

These points are elaborated on in several novel paragraphs and figure panels as follows:

> *Biallelic hits provide insights beyond mutational processes. The rate of biallelic mutation is proportional to that of parallel mutation between clones and increases with both the number of lineages considered and total mutation burden (**Figure S10**). When constructing phylogenies from ever more exhaustive multi-sample or single-cell data[21,22], biallelic mutations provide an estimate for the number of parallel events.*
>
> *Using single-sample bulk sequencing to establish evolutionary relationships between subclones is challenging[4,23]. Under the infinite sites assumption, one can examine rare pairs of phaseable SNVs in regions without copy number gains[4,24]. Specifically, a pattern where one SNV is only found on a subset of the reads reporting the other evidences a linear relationship (**Figure 6f**). In PCAWG melanomas, however, a median 67% of these pairs in diploid regions reflect phylogenetically uninformative biallelic parallel mutations (**Figure 6g**). To avoid biasing phylogenies, biallelic SNVs should be filtered by restricting analyses to haploid regions or scrutinising the VAF and the likelihood of biallelic mutation in the sample[4]. SNV clustering approaches, which rely on the infinite sites assumption for subclonal reconstruction and assignment of each variant to a specific*

*lineage, may pick up "superclonal clusters" of biallelic parallel mutations, but are otherwise expected to remain robust at the levels identified here (**Figure 6h**)[23].*

*Phasing is also used to boost the accuracy of variant callers for single molecule sequencing data[25]. As with multi-allelic variants, relaxation of the set of allowed haplotypes will need to be considered to capture the full extent of somatic variation. Indeed, while only 2.8% of biallelic hits fall within or near exons, we identify 8 candidate biallelic driver events. Parallel nonsense mutations in tumour suppressors ASXL2 and CDKN2A, and divergent events in ERBB4, suggest that in rare cases, biallelic mutations are selected for (**Figure 6i**, **Table S7**).*

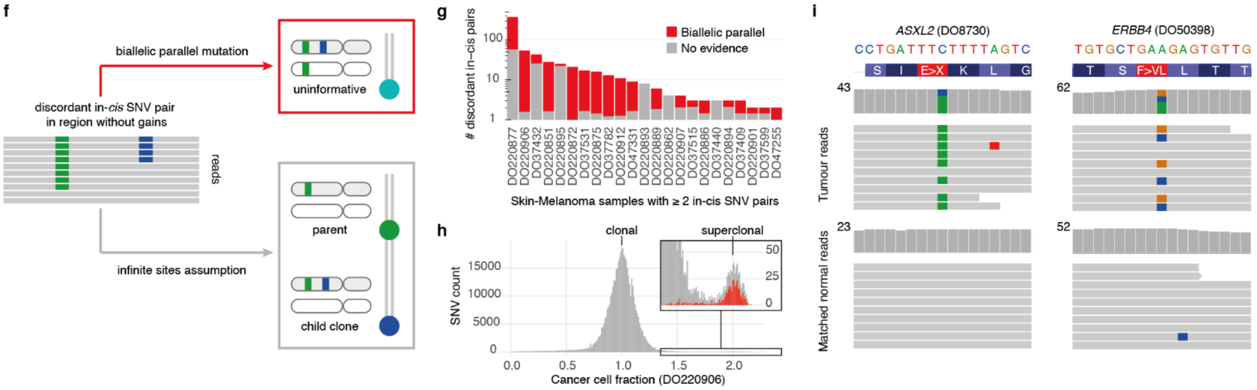

Figure 6 | Biallelic mutations reveal hotspot motifs and confound phylogenies
*… (**f**) Patterns of in-cis SNV pairs in a diploid region evidence linear phylogenies (parent-child) when the infinite sites assumption holds. (**g**) Bar plot showing the number of in-cis SNV pairs in PCAWG melanoma samples with at least two such pairs. Bar height reflects total numbers observed while the red portion indicates the fraction of all pairs with evidence for biallelic parallel mutation (beta-binomial p-value ≤ 0.05 or phasing to a heterozygous SNP). (**h**) Histogram of cancer cell fractions of SNVs in melanoma DO220906. The clonal cluster and a superclonal cluster containing mainly biallelic parallel mutations (red), are indicated. (**i**) IGV visualisation of two missed biallelic drivers in colorectal and oesophageal adenocarcinomas DO8730 and DO50398, respectively. Reads (horizontal bars) are downsampled for clarity and local base-wise coverage is indicated left of the histograms.*

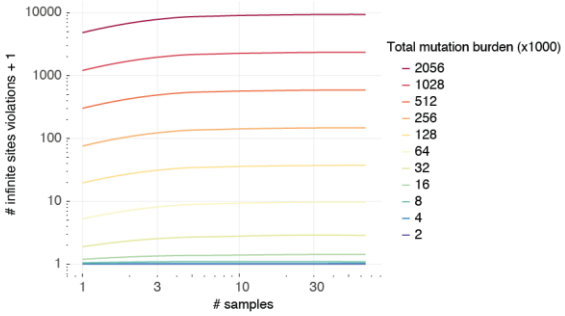

Figure S10 | Infinite sites violations in a multi-sample setting

Simulation results showing how the number of infinite sites violations increases when multiple samples are considered with the indicated total mutational load. Note that in an actual multi-sample setting, the total mutation burden generally increases with the number of samples.

- Parallel mutations are not called in regions with LOH because those mutations are indistinguishable from early mutations. Is it possible that some false positives calls are made due to inaccurate LOH calls? No matter how LOH is called, I would think that a non-trivial segment of the genome is not accurately identified as such.

We thank the reviewer for the opportunity to elaborate on this crucial point. Regions with loss of heterozygosity (and at least one gain) actually represent a special case of a more general pattern restricting the detection of biallelic parallel mutations. Specifically, absent phasing information, we can only confidently call a biallelic mutation when its variant allele frequency suggests it is present on more copies than the major allele tumour copy number, *i.e.*, the variant must be present on both parental alleles. As there are no remaining copies of the other/minor allele in regions with LOH, there is no point in testing SNVs in such regions. Rather, filtering of LOH regions is part of our strategy to alleviate the multiple testing burden, together with an independent filtering step removing sites with lower read coverage. This is now illustrated in the novel **Figure S3a** (included below), which also details the relatively small impact of this filtering step. Note that this is the only time at which the PCAWG copy is used, as the remainder of the pipeline leverages the more robust phased and segmented BAF and LogR, consensus purity and ploidy.

To further address this question, we have evaluated the effect of omitting the filter against regions with LOH without re-adjusting *P*-values for multiple testing. This represents a worst-case scenario of having missed all regions with clonal LOH in PCAWG and not correcting for the additional loss in statistical power due to the increased number of SNVs being tested. In total, this exercise adds 188 VAF hits (1.7% of new total 11,080) and 19 based on phasing (0.5% of new total 3,903). It is worth noting that the presence of phasing hits suggests that ~28% (0.5%/1.7%) of the hits are in fact true biallelic parallel mutations, likely having occurred in regions with subclonal LOH not reflected in the PCAWG clonal consensus copy number. In any case, the final precision and lower-bound recall estimates in this worst-case scenario are

0.816 and 0.473, respectively, which are only marginally lower than the 0.820 and 0.475 obtained with LOH filter (**Figure S3a**). In conclusion, inaccurate LOH calls are not expected to significantly affect our findings.

We realise now our original statement on the detection pipeline, LOH, and early mutations may cause confusion and have rephrased this as follows (also includes new multi-sample results):

> *Without phasing information, we can only detect parallel mutations on more copies than the major allele tumour copy number. Hence, no parallel mutations are called in regions with loss of heterozygosity and late or subclonal events are likely to be underrepresented. Insights into the latter can be glimpsed from multi-sample studies. In a cohort of metastatic prostate cancer with sequencing of matched primary and metastases[12,13], we discern early clonal (preceding the most recent common ancestor) as well as candidate late and subclonal events (**Figure S4**).*

a

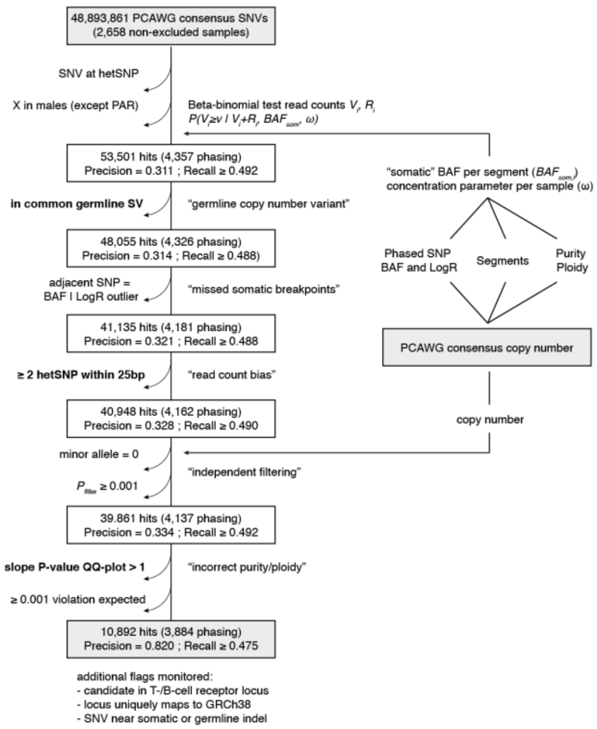

Figure S3 | Detection of biallelic parallel mutations by allele frequency

*(**a**) Flow chart showing the filtering steps, phasing-based estimates of precision and lower bound recall, as well as the input and output data for our pipeline to detect biallelic parallel mutations in*

PCAWG based on variant allele frequencies (VAF). Three filtering steps highlighted in bold are further illustrated in panels (b-d)

Minor comments.

- According to Supplemental Table 4, in cases with divergent mutations, they seem to comprise on average 0.01% of the total mutation burden. Do these cases share any clinicobiological traits or is it likely that these mutations constitute passenger events resulting from of a high mutation rate?

The reviewer is correct that in the set of tumours selected for variant re-calling using Mutect2 the biallelic parallel mutations make up on average 0.01% of the total mutation burden. However, there is considerable variability and this ratio is not expected to be constant. Indeed, as with the total biallelic mutation burden (novel **Figure 5d**), the number of divergent hits increases with the square of the total mutation burden and so this ratio increases rapidly with the total burden. Interestingly, as the reviewer hints at, the proportionality factor is dependent on the tumour type (novel **Figure 5e**). Looking at biallelic divergent mutations specifically, their proportion (of total biallelic hits) is determined by the active mutational processes, as some are much more likely to generate divergent mutations than others (e.g. SBS17 in oesophageal adenocarcinoma *vs.* SBS7, UV-damage, in skin melanoma; **Figure 2d**).

We include a figure for the reviewer below which shows the result of these effects for biallelic divergent mutations and plots their number as a percentage of the total mutation burden (**Figure R2**). Last, while we identify a candidate biallelic divergent driver in *ERBB4* in a case of oesophageal adenocarcinoma (novel **Figure 6i**), the vast majority of such mutations are likely to represent passenger events accumulating due to the ever increasing somatic burden.

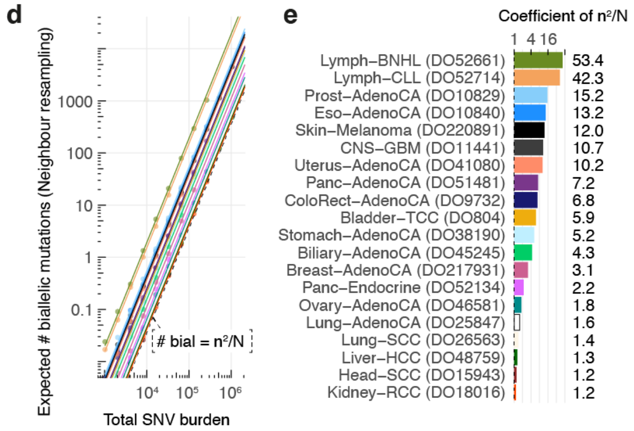

Figure 5 | Comparison between observed and simulated biallelic mutations

*… (**d**) Number of biallelic violations expected according to the neighbour resampling model for a range of mutation burdens and tumour types. The dashed line indicates the birthday problem*

*estimate equal to the square of the mutation burden divided by the genome size (m^2/N). Full coloured lines are the linear fits per tumour type. (**e**) Bar plot of the fitted coefficients of m^2/N as derived in (d). For each tumour type, the ICGC donor ID indicates the representative tumour used.*

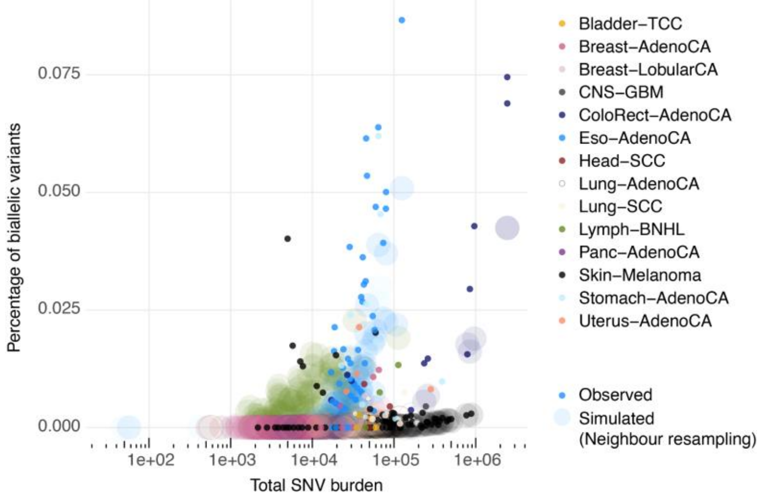

Figure R2 | Percentage of biallelic divergent mutations vs. total burden
Scatter plot showing the observed and simulated (neighbour resampling) percentage of SNVs which are biallelic divergent in tumours. Oesophageal and stomach adenocarcinomas exhibit high rates of biallelic divergent hits at a relatively low total mutation burden compared to other tumour types, most notably melanoma. Only tumour types of which at least one sample was included in the variant recalling are shown.

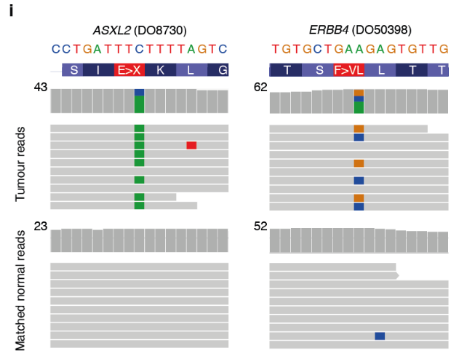

Figure 6 | Biallelic mutations reveal hotspot motifs and confound phylogenies
*… (**i**) IGV visualisation of two missed biallelic drivers in colorectal and oesophageal adenocarcinomas DO8730 and DO50398, respectively. Reads (horizontal bars) are downsampled for clarity and local base-wise coverage is indicated left of the histograms.*

- Row 131: The reference to figure 3C should likely point to figure 2C.

We thank the reviewer for their keen eye and have corrected this mistake.

- The contents of figure 6d needs to be clarified in some more detail. What do the individual data points represent and what does increased quantum yield mean in this context?

We have expanded the panel legend to include more details and have rephrased the label in the panel itself. We hope this clarifies matters and allows our results to be more easily understood by a wide audience. In addition, we have included a novel **Table S7** containing the structure IDs, distances and angles as extracted from the RCSB protein data bank structures.

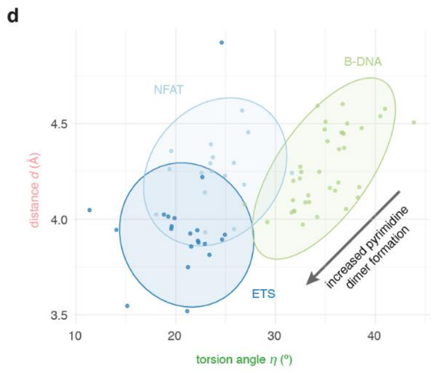

Figure 6 | Biallelic mutations reveal hypermutable motifs and can confound phylogenies
*… (**d**) Scatter plot showing the distance d and angle η indicated in (c) for TpC dinucleotides in structures of ETS-bound (dark blue), NFAT-bound (blue) or free B-form DNA (green) obtained from the RCSB protein data bank (**Table S7**). Ellipses represent the normal-probability contours of each group. Lower values of d and η increase the yield of UV-based pyrimidine dimer formation, as indicated by the arrow. …*

- Supplementary table 3 and 5 are missing data in the chr and position columns.

We apologise to the reviewer, these columns had been omitted to de-identify the data and avoid issues with providing controlled access PCAWG data, while still allowing detailed insights into the biallelic sites. We have raised the issue with data managers, GDC, dbGAP and TCGA senior staff and can provide full details for variants from the ICGC portion (70%) of PCAWG. Somatic variant calls for cases contributed by TCGA however (30%) are under controlled access. Unfortunately, our biallelic calls can not readily be hosted on

TCGA repositories or dbGAP since this project is not a TCGA project proper and is not supported by NIH funding. Instead, to ensure readers with the appropriate TCGA access agreements in place can reconstruct the full table, we have added an index column which refers to the position of the variant in the PCAWG consensus SNV calls for the corresponding tumour. We hope this solution is acceptable and have added the relevant guidance to the table legends (included below).

Table S3 | Biallelic parallel mutations in PCAWG
List of all identified biallelic parallel mutations in PCAWG with read counts and additional quality control metrics. Chromosome and position info for TCGA cases are under controlled access and have been omitted from the table. To reconstruct the full table, the index column refers to the position of the variant in the consensus SNV calls for the corresponding tumour. Somatic variant call details for ICGC cases are provided in full.

Table S5 | Biallelic divergent mutations in 195 PCAWG samples
List of identified biallelic divergent mutations with read counts and additional quality control metrics. Chromosome and position info for TCGA cases are under controlled access and have been omitted from the table. To reconstruct the full table, the index column refers to the position of the variant in the consensus SNV calls for the corresponding tumour, where available. Somatic variant call details for ICGC cases are provided in full.

Reviewer #3:
Remarks to the Author:
Demeulemeester and colleagues report biallelic mutations in cancer whole genome sequencing data which have implications for the validity of some assumptions often used in evolutionary and cancer genome analysis. With simulation and re-sampling studies they demonstrate that despite the typically small number of substitution mutations found in cancers relative to the size of the genome, their sequence context preferences within a cancer make it reasonably likely that both (diploid) copies of a site could be mutated independently in the same tumour. They go on to show that such sites can be found in publicly available cancer genome sequences based on variant allele frequency (VAF) and careful filtering by several methods. Cases of both parallel mutation (both copies change in the same way) and divergent mutation (the two copies change in different ways) are observed, as would logically be expected, their relative ratio depends on the mutation spectrum of the corresponding tumour.
Observed biallelic mutations are typically far more frequent than would be expected based on sampling mutations from similar tumours with closely matched mutation spectra indicating that the analysis of biallelic mutations captures aspects of tumour specific biology and mutation processes that are often not common between “matched” tumours. The authors go on to give examples of extreme hot-spot mutagenesis that correspond to biallelic mutation sites and provide some mechanistic explanations including validation of UV+ETS factor and hairpin+APOBEC hotspots and indicate that the transcription factor NFAT could have similar properties to ETS factors in UV mutagenesis.

This work is important because it highlights that some intuitive expectations about the randomness and chance co-occurrence of mutations at a specific site will often lead to under-estimating the expected frequency of coincidental mutations. It is likely an important part of the explanation for why some genomic sites appear to be mutational hotspots in cancer, and in genomes in general. The manuscript will be of interest to researchers with an interest in mutational processes, mutation spectra, selection under clonal expansion and the broad field of cancer genomics. In terms of scope it would seem a good fit with Nature Genetics.

The study appears well designed and executed. The manuscript is very clearly written with a good narrative and high quality figures. Methods are appropriately detailed though important information is missing from the supplementary files as noted below.

We thank the reviewer for their rigorous assessment of our work and have provided a response to the issues raised below.

Have a small number of concerns that I would like addressed or clarified before recommending publication.

#1. Framing the work in the context of the infinite sites model is slightly misleading. The two modes of biallelic mutation (parallel and divergent) are not violations of the infinite sites model as there are two copies of each allelic site, each being mutated once, which conforms to infinite sites. Violations in cancer genome analysis under this scenario arise through the artificial treatment of the (approximately) diploid genome as haploid. In contrast the monoallelic forward and backward mutations, which are not the focus of this work, are violations of infinite sites as they mutate the same site multiple times.

The reviewer is entirely correct. It is important to note however, that this treatment of a genome as haploid does not reflect an actual choice: short reads can generally not be traced back to their allele of origin. This is likely why previous studies probing into the infinite sites assumption (e.g. Kuipers et al., *Genome Biol.*, 2017) have similarly not made the distinction. We hope that, by now explicitly raising this issue in the text, the reviewer will agree that retaining the current terminology and context will aid the recognition and dissemination of our findings.

Our revised manuscript now makes the following notes of the issue:

> *In addition, breakdown of the infinite sites assumption at the scale of individual single nucleotide variants (SNVs) was inferred from single-cell tumour sequencing data and flagged as a confounder during phylogenetic reconstruction[8]. In bulk tumour data, population averaging and limited long-range information make it difficult to assess mutational recurrence and its impact on analyses.*
>
> *In a single diploid lineage, four classes of infinite sites violations may be considered (**Figure 1**): (i) biallelic parallel and (ii) biallelic divergent, where two alleles independently mutate to the same or different alternate bases, respectively; (iii) monoallelic forward and (iv) monoallelic back, where one variant is mutated to another or back to wild type, respectively. We focus on biallelic mutations, which become problematic when artificially treating genomes as haploid, hypothesising these may be observed directly in bulk tumour genome sequencing data. Loss of variants owing to large-scale genomic deletion does not strictly contradict the infinite sites assumption, yet should be accounted for in cancer genomes[2,8,9].*

#2. The role of selection is not considered in the manuscript. While I accept that the signals reported are almost certainly dominated by mutability, the data are collated from tumours which are subject to positive selection for oncogenic driver mutations. As selection is not excluded as a contributor to biallelic mutations (e.g. additive benefit from biallelic promoter TFBS mutations) it should be at least considered even if ultimately dismissed.

We thank the reviewer for the suggestion to explore this aspect further. As indicated, leveraging only tumour data, it is difficult to exclude any contributions of selection to biallelic mutations. In the revised

manuscript we highlight several cases where selection is likely to play a key role as well as cases where we have been able to show that selection is unlikely to drive mutational recurrence. Specifically, we have included a novel **Figure 6i** illustrating 2 of the 8 candidate biallelic driver events we have identified. Parallel nonsense mutations in tumour suppressors *ASXL2* and *CDKN2A* and a biallelic divergent hit to *ERBB4* are expected to affect tumour cell fitness and hence be subject to selection. We have also assessed transcriptional effects of the recurrent (biallelic) promoter mutations observed in melanoma (**Figure 6a**), using the available PCAWG RNA-Seq data. A novel **Figure S9** shows no discernable impact of mutations at the identified promoter loci on total or allele-specific expression, let alone additive effects of the biallelic hits. This latter result, together with the correlation of biallelic mutations with pan-cancer locus mutability (**Figure S7**), the accuracy of the neighbour resampling simulations in recapitulating both the spectrum and number of biallelic events (**Figure 5a–c**), the absence of recurrent biallelic mutations in coding regions (**Figure 6a**) and the motif enrichment results (**Figure 6a–d**), all support the notion that the bulk of biallelic mutations represent passenger events.

These new analyses are included in the manuscript as follows:

> *Biallelic mutations are not associated with somatic rearrangements ($p_{adj} \geq 0.31$; Mann-Whitney U-test, Benjamini–Hochberg) but occur at loci with a higher mutation rate (**Figure S7**), some of which harbour recurrent biallelic events (**Figure 6a**). The promoter of RPL18A shows three parallel, one divergent, and nine single mutations at chr19:17,970,682, all in melanoma (12% total, **Figure S8**)*[14]*. Motif enrichment at loci with biallelic vs. trinucleotide-matched monoallelic hits in melanoma reveals enrichment of YCTTCCGG and WTTTCC motifs (**Figure 6a–b**)*[15]*.*
>
> …
>
> *While we cannot formally exclude selection as a contributor to these recurrent mutations, no effects on total or allele-specific expression of genes with biallelic promoter mutations could be observed (**Figure S9**).*

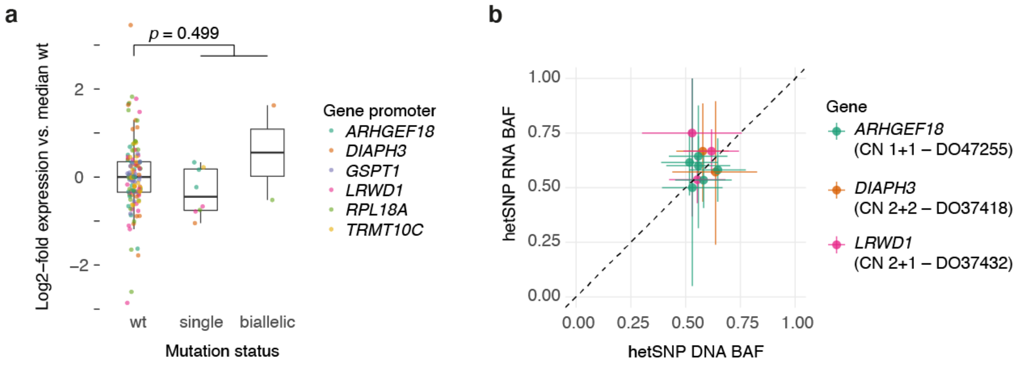

Figure S9 | Effect of promoter mutation on gene expression for genes with biallelic hits

*(**a**) Box and scatter plot showing the log2-fold change in expression compared to the median wild type for promoter mutated genes in **Figure 6a**. Each dot represents the relative expression in a single PCAWG melanoma with RNA-Seq data, stratified by the mutation status of that gene's promoter. (**b**) Scatter plot with posterior 95% highest density intervals for the DNA and RNA B-allele frequencies of expressed germline heterozygous SNPs in the genes/samples with a single mutant promoter allele in (a). The ICGC donor ID and local consensus copy number are indicated.*

The penultimate paragraph now includes the following:

*Indeed, while only 2.8% of biallelic hits fall within or near exons, we identify 8 candidate biallelic driver events. Parallel nonsense mutations in tumour suppressors ASXL2 and CDKN2A, and divergent events in ERBB4, suggest that in rare cases, biallelic mutations are selected for (**Figure 6i**, **Table S7**).*

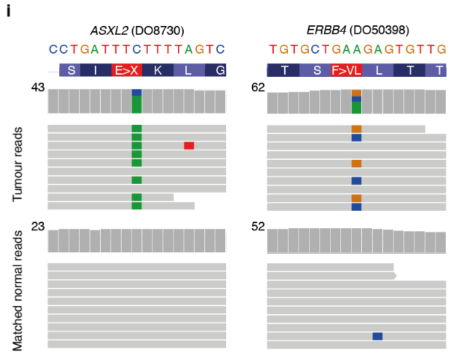

Figure 6 | Biallelic mutations reveal hotspot motifs and confound phylogenies
*… (**i**) IGV visualisation of two missed biallelic drivers in colorectal and oesophageal adenocarcinomas DO8730 and DO50398, respectively. Reads (horizontal bars) are downsampled for clarity and local base-wise coverage is indicated left of the histograms.*

Last, the methods section has also been updated to describe these analyses:

Gene expression analysis
We obtained gene expression data from PCAWG (Synapse ID syn5553991; http://dcc.icgc.org/releases/PCAWG/transcriptome/gene_expression/)[28]. Briefly, reads were aligned with both TopHat2 (v.2.0.12) and STAR (v.2.4.0i, two-pass). Read counts for genes were calculated using htseq-count and the GENCODE v19 annotation. Counts were normalized using FPKM and upper quartile normalization: i.e. FPKM-UQ $= (C \times 10^9)/(UQ \times L \times G)$ *where C is the fragment count, L the length of the gene, UQ the upper quartile of the fragment count in protein coding genes on autosomes and G the number of such protein coding genes. The final expression*

values are an average of the TopHat2 and STAR-based alignments. FPKM-UQ values for genes with recurrent (biallelic) promoter mutations in melanoma were extracted and stratified by promoter mutation status in the tumour (wild type, single SNV, biallelic mutation). Per gene, log2 fold change values were computed compared to the median expression from the wild type promoter. To assess whether single SNVs at these promoter sites induce allele-specific expression, we used RSamtools to pile up base counts from the STAR-aligned bam files at the locations of heterozygous germline SNPs in the patient as identified from the matching normal genome by Battenberg. Posterior 95% highest density intervals were computed for the DNA and RNA base counts assuming a uniform $Beta(1,1)$ *prior and a binomial likelihood. Non-overlapping intervals can be indicative of allele-specific expression.*

#3. The most striking results for me are those presented in Figure 5c,d. I can envisage wanting to cite this work but would want to give examples of specific cancer types and their corresponding fold-excess of biallelic mutations over expectation from between tumour sampling. However, only two such figures are vaguely given ("over 8 to 32-fold excess biallelic mutations") and the relate to what is defined as the less accurate method. I'd really like to see the data in these to figure panels plotted as frequency distributions for each cancer type, and a median fold value associated. This value then essentially captures some aspect of the mutational process uniqueness of each tumour in the cancer type.

We thank the reviewer for this insightful comment, allowing us to highlight what is indeed a take-home message of our manuscript. In the revised manuscript, we have extended these analyses comparing observed *vs*. expected biallelic hits. In addition, as there may have been some confusion regarding which simulation approach provides which results, we focus this part on the more accurate neighbour resampling model and –also in response to reviewer #1 point #2– provide a schematic overview of both approaches early on in the revised text (**Figure S1**).

Briefly, the original panels **Figure 5c,d** have been updated with the latest calls and neighbour resampling simulations, resulting in improved correlations, and are moved to the supplement (**Figure S6a,b**). In the new main **Figure 5c**, we focus on the neighbour resampling model only, showing how the ratio between observed and expected counts even more closely approximates 1:1 when the phasing-based biallelic parallel mutation estimates are leveraged in cases with a high mutation (and multiple testing) burden.
We subsequently adapt this confirmed neighbour resampling model to simulate a range of mutation burdens across tumour types. This demonstrates how the number of biallelic mutations is indeed proportional to the square of the total mutation burden as suggested by the birthday problem approximation (**Figure 5d**). We then quantify the corresponding tumour-type specific coefficient which scales the callable genome size and provides the fold-excess of biallelic mutations over what is naively expected. Notably, this provides straightforward estimation of the number of violations in any tumour of a given type with a given mutation burden (**Figure 5e**).

These points and figures are included in the revised manuscript as follows:

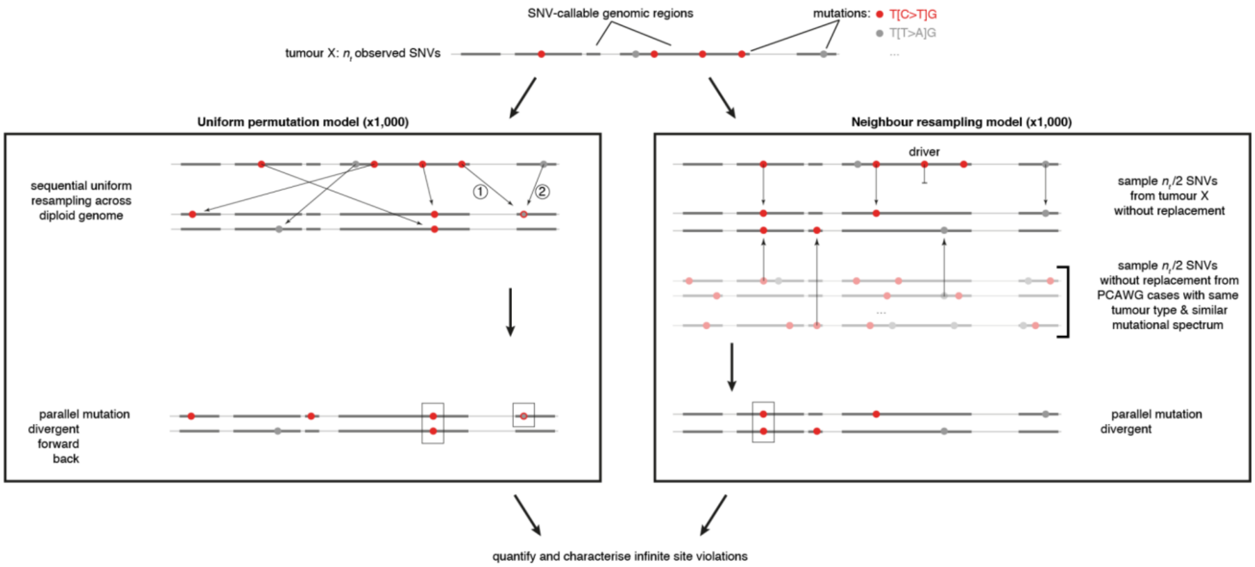

Figure S1 | Simulation approaches for infinite sites model violations
Schematic overview of the uniform permutation (left) and neighbour resampling (right) approaches to assess the number and type of infinite sites violations expected in a tumour. Numbers in the uniform permutation panel highlight the sequential nature of the sampling, which keeps track of mutated positions to consider them accordingly for biallelic, forward, and back mutation. Note that the neighbour resampling model excludes all PCAWG annotated driver mutations and allows simulation of biallelic events only.

The relevant portion of the main text now states:

*While the uniform permutation model underestimates, neighbour resampling accurately predicts the number of biallelic mutations (**Figure 5c, S6a–b**). Resampling mutation burdens and tumour types with the confirmed model demonstrates how biallelic mutations are proportional to the square of the mutation burden (m^2, **Figure 5d**). The coefficient per tumour type (C_{type}) scales the callable genome size (N) and provides straightforward estimation of the number of violations as $C_{type}m^2/N$ (**Figure 5d–e**).*

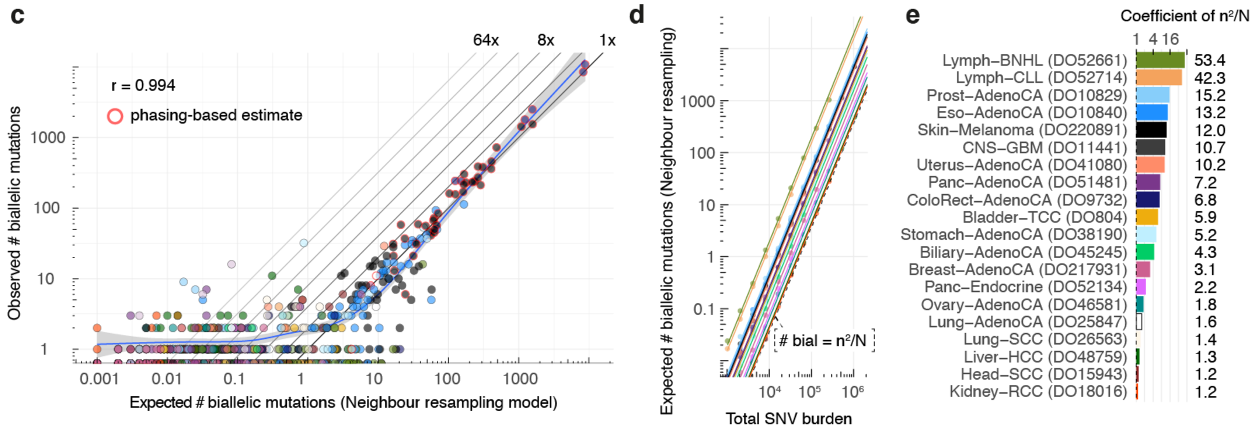

Figure 5 | Comparison between observed and simulated biallelic mutations

*… (**c**) Scatterplot of the observed vs. neighbour resampling model-expected number of biallelic mutations (parallel + divergent) for all PCAWG tumours. For cases with ≥10,000 phaseable SNVs (red borders), the phasing-based number is provided. Colours reflect tumour type as in **Figure 2**. A spline regression fit is shown together with the Pearson correlation. (**d**) Number of biallelic violations expected according to the neighbour resampling model for a range of mutation burdens and tumour types. The dashed line indicates the birthday problem estimate equal to the square of the mutation burden divided by the genome size (m^2/N). Full coloured lines are the linear fits per tumour type. (**e**) Bar plot of the fitted coefficients of m^2/N as derived in (d). For each tumour type, the ICGC donor ID indicates the representative tumour used.*

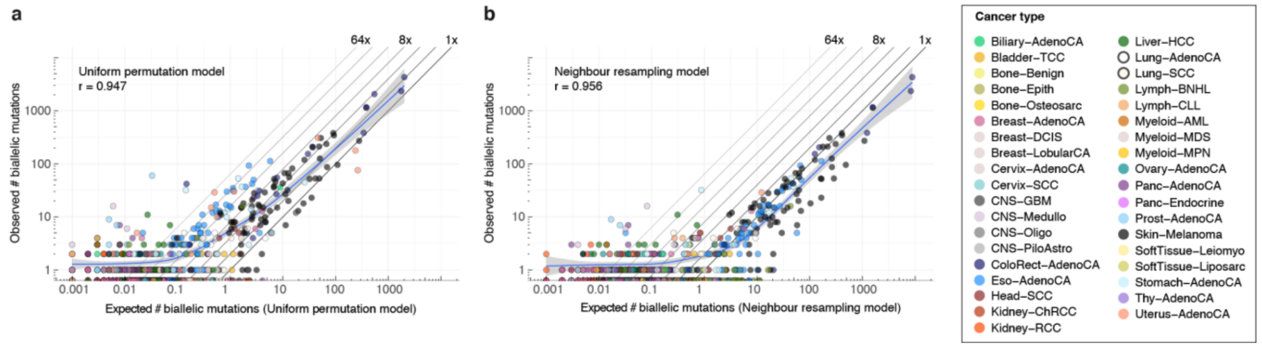

Figure S6 | Comparison between observed and simulated biallelic mutations

*(**a**,**b**) Scatterplots of the observed vs. expected number of biallelic mutations (parallel + divergent) for all PCAWG tumours using the uniform permutation (**a**) and neighbour resampling models (**b**). A spline regression fit is shown together with the Pearson correlation.*

The corresponding methods description, extending the neighbour resampling section, reads:

In a second simulation approach (termed neighbour resampling model), we resample without replacement the mutational landscape of a tumour from the empirical mutation distribution, minus the annotated driver SNVs (Synapse ID: syn11639581 and https://dcc.icgc.org/releases/PCAWG/driver_mutations/). Specifically, in each simulation, we randomly pick 50% of the observed mutations in the original tumour and resample the other 50% from the pooled SNVs of representative PCAWG tumours. In this context, we define a tumour as representative for the simulation target when it is of the same tumour type (PCAWG histology) and has similar mutational signature exposures (cosine similarity of their mutation spectra ≥ 0.9)[11]. This can be viewed as sampling one allele from the original tumour and one allele from the corresponding empirical mutation distribution.

...

When simulating a range of mutational burdens for a tumour type, we leverage the tumour with the highest number of representative tumours (≥ 10) and with a total number of mutations to sample from exceeding 100,000. In this case, all mutations are sampled from the pooled set of mutations, which in this case includes the original tumour.

#4. The study design is essentially a trawl for variants with unusually high VAF that are not present in the non-tumour sample from the same patient, using PCAWG cancer genome sequencing data. Inevitably this will find many alignment problems, copy number alterations, sequence errors and contamination issues. These "noise" signals likely dominate an initial naive screen but the authors have detailed multiple procedures to clean the noise and reveal the signal reported. The correspondence of the biallelic mutations to the predominant mutation signatures for the tumours, and more so to the "simulated violation spectra", along with the SNV-SNP phasing give good confidence that the cleaning has been somewhat successful and there is signal, but it is difficult from the presented results to judge how successful and what fraction of the reported signal remains contaminating noise. Supplementary plots illustrating how such confirmatory metrics change with progressive filtering (or
comparisons to simulated data doped with known levels of noise) may help the reader understand this largely hidden but important aspect of the data processing.

We thank the reviewer for their appreciation of what has indeed been a crucial aspect of our study and the opportunity to highlight the work that has gone into it. To this end we have put together the novel **Figure S3**, which includes a detailed flow chart of our entire VAF-based pipeline for detection of biallelic parallel mutations together with the number of hits and phasing-based estimates of precision and lower bound recall at each step (panel **a**). Figure panels **b-d** further illustrate three of the filtering steps (common germline SVs, read count reference biases and purity/ploidy errors), which we hope will give the reader further insight into their effects and the pitfalls which we have worked to avoid. We hope the addition (included below) will prove to be a valued part of the manuscript for a broad readership.

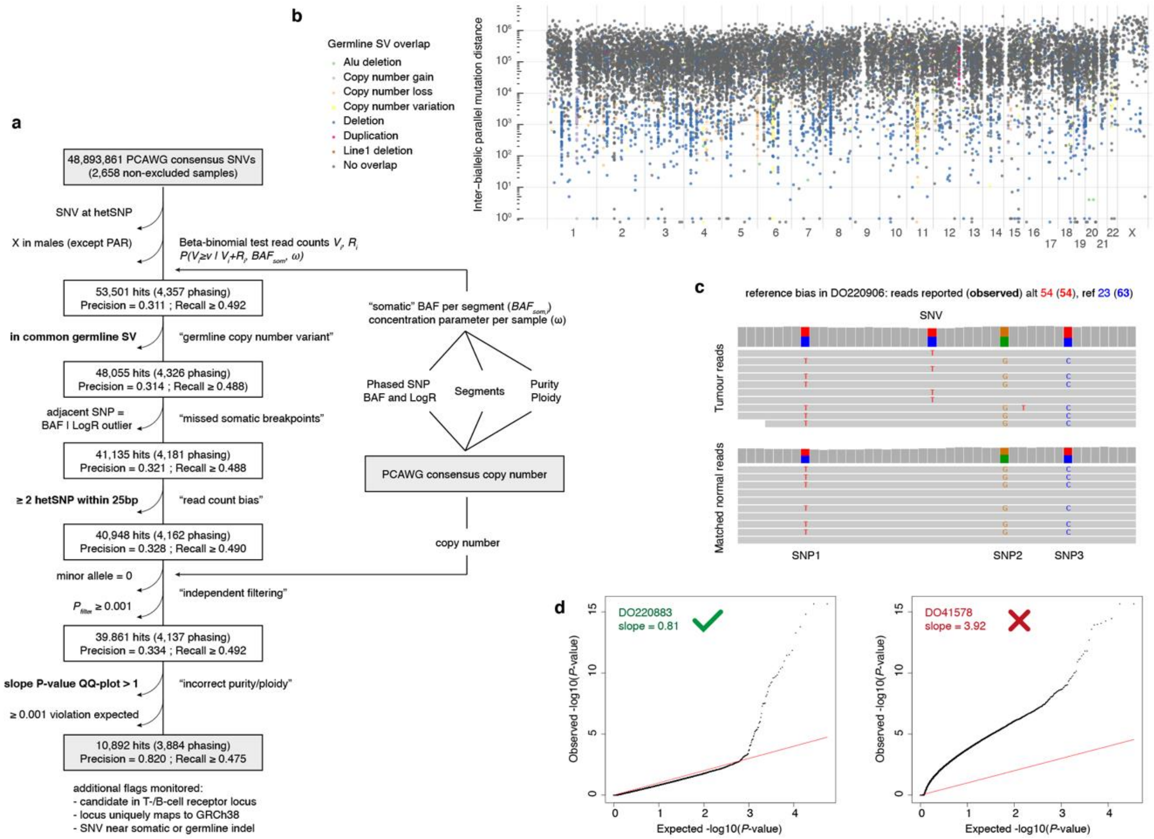

Figure S3 | Detection of biallelic parallel mutations by allele frequency

*(**a**) Flow chart showing the filtering steps, phasing-based estimates of precision and lower bound recall, as well as the input and output data for our pipeline to detect biallelic parallel mutations in PCAWG based on variant allele frequencies (VAF). Three filtering steps highlighted in bold are further illustrated in panels (b-d). (**b**) Rainfall plot of all biallelic parallel hits obtained after omitting the germline SV filter. Streaks of coloured dots indicate a clustering of hits in regions with common germline structural variants. While demonstrating the ability of the pipeline to detect VAF outliers, these hits are poorly supported by phasing data and likely represent single somatic SNVs in the context of a heterozygous germline deletion. (**c**) Example of reference bias in the PCAWG consensus SNV read counts. Reads carrying the somatic variant contain alternate germline alleles at three proximal positions, resulting in an underreporting of the number of wild type reads and an overestimation of the VAF. (**d**) Diagnostic QQ-plots of the one-sided beta-binomial read count test P-values for two samples. DO41578 P-values are overinflated (slope > 1), hinting at consensus purity/ploidy errors, and the sample is excluded as a result.*

#5. Supplementary files. Chromosome identifier and position information are systematically missing from all of the supplementary files. This has precluded reviewer evaluation of data quality for the mutation calls and checking for obvious artefacts in the data. For example checking for pronounced spatial clustering of biallelic mutations or to see if called mutations correspond to known polymorphic sites. The methods report that such human polymorphisms have been filtered but it's a reviewer's prerogative to check.

This issue has been raised by reviewer #2 as well, and we apologise for not clarifying the intended de-identification beforehand. These columns had been omitted to avoid issues with providing controlled access PCAWG data, while still allowing detailed insights into the biallelic sites. We have since raised the issue with data managers, dbGAP and TCGA senior staff and can provide full details for variants from the ICGC portion (70%) of PCAWG. Somatic variant calls for cases contributed by TCGA however (30%) are under controlled access. Unfortunately, our biallelic calls can not readily be hosted on TCGA repositories or dbGAP since this project is not a TCGA project proper and is not supported by NIH funding. Instead, to ensure readers with the appropriate TCGA access agreements in place can reconstruct the full table, we have added an index column which refers to the position of the variant in the PCAWG consensus SNV calls for the corresponding tumour. We hope this solution is acceptable and have added the relevant guidance to the table legends (included below).

> ***Table S3 | Biallelic parallel mutations in PCAWG***
> *List of all identified biallelic parallel mutations in PCAWG with read counts and additional quality control metrics. Chromosome and position info for TCGA cases are under controlled access and have been omitted from the table. To reconstruct the full table, the index column refers to the position of the variant in the consensus SNV calls for the corresponding tumour. Somatic variant call details for ICGC cases are provided in full.*
>
> ***Table S5 | Biallelic divergent mutations in 195 PCAWG samples***
> *List of identified biallelic divergent mutations with read counts and additional quality control metrics. Chromosome and position info for TCGA cases are under controlled access and have been omitted from the table. To reconstruct the full table, the index column refers to the position of the variant in the consensus SNV calls for the corresponding tumour, where available. Somatic variant call details for ICGC cases are provided in full.*

#6. Figure 2 legend. "Tumour histology of the samples" better described as tumour type.

This has been amended.

Decision Letter, first revision:

natureresearch

3rd Sep 2021

Dear Peter,

I hope you're well - have you completed your move yet?

Your Letter, "Biallelic mutations in cancer genomes reveal local mutational determinants" has now been seen by 3 referees. As you know, Reviewer #3 (who has signed their report) had some minor comments which we'd like you to address and I thought that the easiest way to do this would be in the context of a light-touch revision. After that, our plan would be to assess the revisions in house, and move forward.

We therefore invite you to revise your manuscript taking into account all reviewer and editor comments. Please highlight all changes in the manuscript text file. At this stage we will need you to upload a copy of the manuscript in MS Word .docx or similar editable format.

We are committed to providing a fair and constructive peer-review process. Do not hesitate to contact us if there are specific requests from the reviewers that you believe are technically impossible or unlikely to yield a meaningful outcome.

When revising your manuscript:

*1) Include a “Response to referees” document detailing, point-by-point, how you addressed each referee comment. If no action was taken to address a point, you must provide a compelling argument. This response will be sent back to the referees along with the revised manuscript.

*2) If you have not done so already please begin to revise your manuscript so that it conforms to our Letter format instructions, available
here.
Refer also to any guidelines provided in this letter.

*3) Include a revised version of any required Reporting Summary:
https://www.nature.com/documents/nr-reporting-summary.pdf
It will be available to referees (and, potentially, statisticians) to aid in their evaluation if the manuscript goes back for peer review.
A revised checklist is essential for re-review of the paper.

Please be aware of our guidelines on digital image standards.

Please use the link below to submit your revised manuscript and related files:

[REDACTED]

Note: This URL links to your confidential home page and associated information about manuscripts you may have submitted, or that you are reviewing for us. If you wish to forward

this email to co-authors, please delete the link to your homepage.

We hope to receive your revised manuscript within four to eight weeks. If you cannot send it within this time, please let us know.

Please do not hesitate to contact me if you have any questions or would like to discuss these revisions further.

Nature Genetics is committed to improving transparency in authorship. As part of our efforts in this direction, we are now requesting that all authors identified as 'corresponding author' on published papers create and link their Open Researcher and Contributor Identifier (ORCID) with their account on the Manuscript Tracking System (MTS), prior to acceptance. ORCID helps the scientific community achieve unambiguous attribution of all scholarly contributions. You can create and link your ORCID from the home page of the MTS by clicking on 'Modify my Springer Nature account'. For more information please visit please visit www.springernature.com/orcid.

We look forward to seeing the revised manuscript and thank you for the opportunity to review your work.

Sincerely,

Safia

Safia Danovi
Editor
Nature Genetics

Referee expertise:

Reviewers' Comments:

Reviewer #1:
Remarks to the Author:
I thank the authors for their detailed responses to my comments and recognize that a lot of work went into this. I have no further suggestions.

Reviewer #2:
Remarks to the Author:
The authors have addressed our comments carefully. I have no additional comments.

Reviewer #3:
Remarks to the Author:
The authors have done a good job of addressing most of the points I have previously raised and this appears to be the case for constructive comments from other reviewers. I do have some outstanding queries regarding my previous point #3 and a few minor issues that arise from the manuscript edits.

Previous point #1 – Infinite sites pedantry. I am satisfied that the the authors have now sufficiently qualified and justified their terminology. Resolved.

Previous point #2 – The role of selection. I'm very pleased to see the consideration of selection and additional analyses on this theme (e.g. Figure 6i, Figure S9). Evidence for the rare selection of biallelic driver mutations (Table S7) is an interesting and citable additional result. Resolved.

Previous point #3 – Observed versus expected biallelic hits. Thank you, the graphical summary of simulation approaches is a useful addition. The revised and additional analysis quantifying observed versus expected mutations (Figure 5d-e; Figure S6a,b) is very welcome. The Figure 5e analysis showing the coefficients of mutation burden divided by genome size does go some way to addressing my original point. However, it only shows values for a "representative tumour" rather than illustrating the distribution of values for each tumour type. I suspect the reason the authors have chosen this presentation is because many of the tumours exhibit zero observed biallelic mutations. I still think presenting the coeficient metric for each tumour as a distribution per cancer type and a mean or median value shown per cancer type would be an informative and useful addition to the manuscript. For example plots in the style of Alexandrov et al, Nature 2013 Figure 1, again with y-axis (coeficient) plotted on a log-scale. As it currently stands the reader does not know how "representative" the representative samples are.

Previous point #4 – Variant filtering. Thank you, the addition of Figure S3 is a useful addition that provides both a good overview of the filtering pipeline and the key numbers with which one can understand the effect and value of each filtering step. Resolved.

Previous point #5 – Missing chromosomal coordinates. The authors clarification on chromosome coordinate omission and their innovative solution of providing SNV call index position fully satisfies my concern. Resolved.

Previous point #6 – Figure legend. Resolved.

Additional minor points arising from manuscript edits:

Figure 5d,e. Manuscript text and figure legend refer to a coefficient of mutation burden divided by genome size. Mutation burden appears to be shown as n^2 in the figure but m^2 in the legend and main text.

Figure 6, panel a. There has been some editing of the "fifty most frequently mutated loci" since the previous version of the manuscript. A consequence is that the sole annotated lncRNA has been removed so lncRNA can be removed from the figure key. Also the cancer type annotation on the heat-map x-axis is now missing – should be replaced.

Figure 6. panel e. All motif enrichment p-values have been updated but motif-logos appear identical. Do the motif-logos need to be updated?

Martin Taylor

Author Rebuttal, first revision:

Point-by-point response to referees

We thank all reviewers for their careful assessment of our revised manuscript and constructive comments. We address below, in a point-by-point response, the outstanding point #3 of reviewer #3 as well as the minor points that arose during revision.

Reviewers' Comments:

Reviewer #1:
Remarks to the Author:
I thank the authors for their detailed responses to my comments and recognize that a lot of work went into this. I have no further suggestions.

Reviewer #2:
Remarks to the Author:
The authors have addressed our comments carefully. I have no additional comments.

Reviewer #3:
Remarks to the Author:
The authors have done a good job of addressing most of the points I have previously raised and this appears to be the case for constructive comments from other reviewers. I do have some outstanding queries regarding my previous point #3 and a few minor issues that arise from the manuscript edits.

Previous point #1 – Infinite sites pedantry. I am satisfied that the the authors have now sufficiently qualified and justified their terminology. Resolved.

Previous point #2 – The role of selection. I'm very pleased to see the consideration of selection and additional analyses on this theme (e.g. Figure 6i, Figure S9). Evidence for the rare selection of biallelic driver mutations (Table S7) is an interesting and citable additional result. Resolved.

Previous point #3 – Observed versus expected biallelic hits. Thank you, the graphical summary of simulation approaches is a useful addition. The revised and additional analysis quantifying observed versus expected mutations (Figure 5d-e; Figure S6a,b) is very welcome. The Figure 5e analysis showing the coefficients of mutation burden divided by genome size does go some way to addressing my original

point. However, it only shows values for a “representative tumour” rather than illustrating the distribution of values for each tumour type. I suspect the reason the authors have chosen this presentation is because many of the tumours exhibit zero observed biallelic mutations. I still think presenting the coeficient metric for each tumour as a distribution per cancer type and a mean or median value shown per cancer type would be an informative and useful addition to the manuscript. For example plots in the style of Alexandrov et al, Nature 2013 Figure 1, again with y-axis (coeficient) plotted on a log-scale. As it currently stands the reader does not know how “representative” the representative samples are.

We apologise for missing this point during the earlier revision and have now derived estimates for the fold excess biallelic mutations of all PCAWG tumours given their specific mutation spectrum and burden. The new **Figure 5e** shows these coefficients in the fashion suggested by the reviewer, revealing the full distribution per tumour type together with the median. The new figure panel, as well as the updated paragraph, figure legend, and methods section are included below for the reviewers’ convenience.

> *While the uniform permutation model underestimates, neighbour resampling accurately predicts the number of biallelic mutations (**Figure 5c, S6a–b**). Resampling a range of mutation burdens per tumour with the confirmed model demonstrates biallelic mutations are proportional to the square of the mutation burden (n^2, **Figure 5d**). A tumour type-specific coefficient (C_{type}) scales the callable genome size (N) and provides straightforward estimation of the number of violations as $C_{type}n^2/N$ (**Figure 5d–e**).*

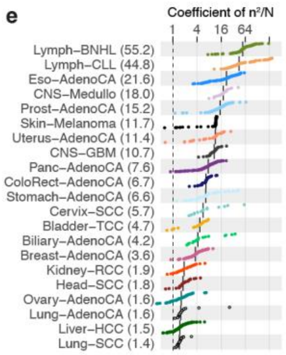

Figure 5 | Comparison between observed and simulated biallelic mutations
*… (**e**) Dot plot of the coefficients of n^2/N as derived in (d). Every dot represents a tumour and black vertical lines represent the median tumour type-specific coefficient C_{type}.*

The relevant methods section reads as follows:

In a second simulation approach (termed neighbour resampling model), we resample without replacement the mutational landscape of a tumour from the empirical mutation distribution, minus the annotated driver SNVs (Synapse ID: syn11639581 and https://dcc.icgc.org/releases/PCAWG/driver_mutations/). Specifically, in each simulation, we randomly pick 50% of the observed mutations in the original tumour and resample the other 50% from the pooled SNVs of representative PCAWG tumours.
…
When simulating a range of mutational burdens for a tumour or estimating its coefficient C (which scales the number of biallelic hits predicted by the birthday problem approximation; see below), all mutations are sampled from the pooled set of mutations, including the original tumour. To limit biases, we require that this pooled set contains at least twice the number of mutations of the sample to be assessed and that ≥3 biallelic hits are observed across 100 simulations. A median coefficient (C_{type}) is computed for tumour types with ≥10 cases meeting these criteria.

Previous point #4 – Variant filtering. Thank you, the addition of Figure S3 is a useful addition that provides both a good overview of the filtering pipeline and the key numbers with which one can understand the effect and value of each filtering step. Resolved.

Previous point #5 – Missing chromosomal coordinates. The authors clarification on chromosome coordinate omission and their innovative solution of providing SNV call index position fully satisfies my concern. Resolved.

Previous point #6 – Figure legend. Resolved.

Additional minor points arising from manuscript edits:

Figure 5d,e. Manuscript text and figure legend refer to a coefficient of mutation burden divided by genome size. Mutation burden appears to be shown as n^2 in the figure but m^2 in the legend and main text.

We thank the reviewer for spotting this error and have made sure the mutation burden is now consistently referred to as *n* across the manuscript.

Figure 6, panel a. There has been some editing of the “fifty most frequently mutated loci” since the previous version of the manuscript. A consequence is that the sole annotated lncRNA has been removed so lncRNA can be removed from the figure key. Also the cancer type annotation on the heat-map x-axis is now missing – should be replaced.

These mistakes have now been fixed and we are grateful to the reviewer for catching them.

Figure 6. panel e. All motif enrichment p-values have been updated but motif-logos appear identical. Do the motif-logos need to be updated?

We apologise for the omission and have updated the sequence logos to the latest version. The final figure is included below for the reviewers' convenience.

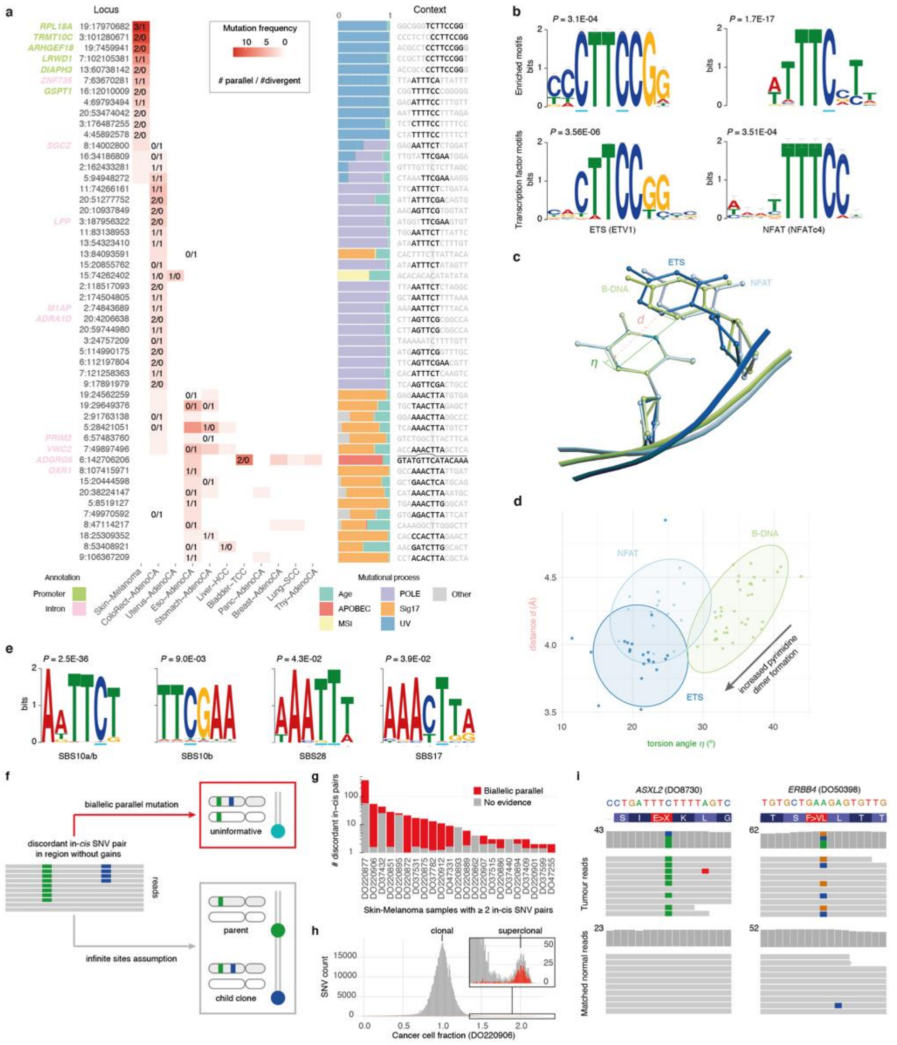

Decision Letter, second revision:

Our ref: NG-LE57231R1

13th Sep 2021

Dear Peter,

Thank you for submitting your revised manuscript "Biallelic mutations in cancer genomes reveal local mutational determinants" (NG-LE57231R1). We have assessed your revisions in-house and we'll be happy in principle to publish it in Nature Genetics, pending minor revisions to satisfy the referees' final requests and to comply with our editorial and formatting guidelines.

If the current version of your manuscript is in a PDF format, please email us a copy of the file in an editable format (Microsoft Word or LaTex)-- we can not proceed with PDFs at this stage.

We are now performing detailed checks on your paper and will send you a checklist detailing our editorial and formatting requirements soon (we're in a very period at the moment so it might take a couple of weeks or so). Please do not upload the final materials and make any revisions until you receive this additional information from us.

Thank you again for your interest in Nature Genetics Please do not hesitate to contact me if you have any questions.

Sincerely,

Safia

Safia Danovi
Editor
Nature Genetics

Final Decision Letter:

In reply please quote: NG-LE57231R2 Van Loo

14th Dec 2021

Dear Dr. Van Loo,

I am delighted to say that your manuscript "Biallelic mutations in cancer genomes reveal local mutational determinants" has been accepted for publication in an upcoming issue of Nature Genetics.

Over the next few weeks, your paper will be copyedited to ensure that it conforms to Nature Genetics style. Once your paper is typeset, you will receive an email with a link to choose the appropriate publishing options for your paper and our Author Services team will be in touch regarding any additional information that may be required.

After the grant of rights is completed, you will receive a link to your electronic proof via email with a request to make any corrections within 48 hours. If, when you receive your proof, you cannot meet this deadline, please inform us at rjsproduction@springernature.com immediately.

You will not receive your proofs until the publishing agreement has been received through our system.

Due to the importance of these deadlines, we ask that you please let us know now whether you will be difficult to contact over the next month. If this is the case, we ask you provide us with the contact information (email, phone and fax) of someone who will be able to check the proofs on your behalf, and who will be available to address any last-minute problems.

Your paper will be published online after we receive your corrections and will appear in print in the next available issue. You can find out your date of online publication by contacting the Nature Press Office (press@nature.com) after sending your e-proof corrections. Now is the time to inform your Public Relations or Press Office about your paper, as they might be interested in promoting its publication. This will allow them time to prepare an accurate and satisfactory press release. Include your manuscript tracking number (NG-LE57231R2) and the name of the journal, which they will need when they contact our Press Office.

Before your paper is published online, we shall be distributing a press release to news organizations worldwide, which may very well include details of your work. We are happy for your institution or funding agency to prepare its own press release, but it must mention the embargo date and Nature Genetics. Our Press Office may contact you closer to the time of publication, but if you or your Press Office have any enquiries in the meantime, please contact press@nature.com.

Acceptance is conditional on the data in the manuscript not being published elsewhere, or announced in the print or electronic media, until the embargo/publication date. These restrictions are not intended to deter you from presenting your data at academic meetings and conferences, but any enquiries from the media about papers not yet scheduled for publication should be referred to us.

Please note that <i>Nature Genetics</i> is a Transformative Journal (TJ). Authors may publish their research with us through the traditional subscription access route or make their paper immediately open access through payment of an article-processing charge (APC). Authors will not be required to make a final decision about access to their article until it has been accepted. Find out more about Transformative Journals

<B>Authors may need to take specific actions to achieve compliance with funder and institutional open access mandates. For submissions from January 2021, if your research is supported by a funder that requires immediate open access (e.g. according to Plan S principles) then you should select the gold OA route, and we will direct you to the compliant route where possible. For authors selecting the subscription publication route our standard licensing terms will need to be accepted, including our self-archiving policies. Those standard licensing terms will supersede any other terms that the author or any third party may assert apply to any version of the

manuscript.

Please note that Nature Research offers an immediate open access option only for papers that were first submitted after 1 January, 2021.

If you have any questions about our publishing options, costs, Open Access requirements, or our legal forms, please contact ASJournals@springernature.com

If you have posted a preprint on any preprint server, please ensure that the preprint details are updated with a publication reference, including the DOI and a URL to the published version of the article on the journal website.

To assist our authors in disseminating their research to the broader community, our SharedIt initiative provides you with a unique shareable link that will allow anyone (with or without a subscription) to read the published article. Recipients of the link with a subscription will also be able to download and print the PDF.

As soon as your article is published, you will receive an automated email with your shareable link.

You can now use a single sign-on for all your accounts, view the status of all your manuscript submissions and reviews, access usage statistics for your published articles and download a record of your refereeing activity for the Nature journals.

An online order form for reprints of your paper is available at https://www.nature.com/reprints/author-reprints.html. Please let your coauthors and your institutions' public affairs office know that they are also welcome to order reprints by this method.

If you have not already done so, we invite you to upload the step-by-step protocols used in this manuscript to the Protocols Exchange, part of our on-line web resource, natureprotocols.com. If you complete the upload by the time you receive your manuscript proofs, we can insert links in your article that lead directly to the protocol details. Your protocol will be made freely available upon publication of your paper. By participating in natureprotocols.com, you are enabling researchers to more readily reproduce or adapt the methodology you use. Natureprotocols.com is fully searchable, providing your protocols and paper with increased utility and visibility. Please submit your protocol to https://protocolexchange.researchsquare.com/. After entering your nature.com username and password you will need to enter your manuscript number (NG-LE57231R2). Further information can be found at https://www.nature.com/nprot/.

Sincerely,

Safia Danovi
Editor
Nature Genetics
